# Multivariate Modulation of the Zr MOF UiO‐66 for Defect‐Controlled Combination Anticancer Drug Delivery

**DOI:** 10.1002/anie.201915848

**Published:** 2020-02-04

**Authors:** Isabel Abánades Lázaro, Connor J. R. Wells, Ross S. Forgan

**Affiliations:** ^1^ WestCHEM School of Chemistry University of Glasgow Joseph Black Building University Avenue Glasgow G12 8QQ UK

**Keywords:** anticancer, cytotoxicity, drug delivery, metal–organic frameworks, microporous materials

## Abstract

Metal–organic frameworks (MOFs) are emerging as leading candidates for nanoscale drug delivery, as a consequence of their high drug capacities, ease of functionality, and the ability to carefully engineer key physical properties. Despite many anticancer treatment regimens consisting of a cocktail of different drugs, examples of delivery of multiple drugs from one MOF are rare, potentially hampered by difficulties in postsynthetic loading of more than one cargo molecule. Herein, we report a new strategy, multivariate modulation, which allows incorporation of up to three drugs in the Zr MOF UiO‐66 by defect‐loading. The drugs are added to one‐pot solvothermal synthesis and are distributed throughout the MOF at defect sites by coordination to the metal clusters. This tight binding comes with retention of crystallinity and porosity, allowing a fourth drug to be postsynthetically loaded into the MOFs to yield nanoparticles loaded with cocktails of drugs that show enhancements in selective anticancer cytotoxicity against MCF‐7 breast cancer cells in vitro. We believe that multivariate modulation is a significant advance in the application of MOFs in biomedicine, and anticipate the protocol will also be adopted in other areas of MOF chemistry, to easily produce defective MOFs with arrays of highly functionalised pores for potential application in gas separations and catalysis.

## Graphical abstract

**Multivariate modulation**: Inclusion of multiple modulators into one‐pot self‐assembly of MOFs yields UiO‐66 nanoparticles with three drugs loaded at defect sites throughout the structure, yet still sufficient porosity to postsynthetically store a fourth, selectivity‐enhancing, anticancer drug.

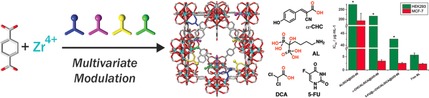

## Introduction

**Figure 1:**
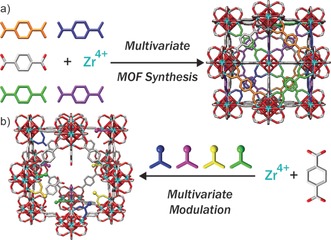
Concepts for synthesis of UiO‐66 as a) a multivariate, or MTV MOF with multiple mixed linkers, or b) using multivariate modulation, which we have termed MTVM, to install multiple different modulators as defects within one MOF.

Metal–organic frameworks (MOFs)^1^ are a new generation of highly porous macromolecular structures composed of metal ions or clusters linked by multidentate organic bridging ligands, which, owing to their attractive properties, have notable potential for applications in different contexts, including gas capture, storage and separation,^2^ catalysis,^3^ water treatment^4^ and drug delivery.^5^ MOFs have almost infinite tunability due to the effectively unlimited range of metal ions and ligands available to form their structures.^6^ Additionally, a class of mixed‐linker materials—so‐called multivariate (MTV) MOFs—have been synthesised, tuning physicochemical properties and their performance in different applications. By introducing multiple analogous linkers into one framework and effectively forming a solid solution of organic functionality within the pores (Figure 1 a), cooperative effects for gas adsorption and heterogeneous catalysis have been found,^7^ for example, Yaghi et al. synthesised MTV‐MOF‐5 type structures that contain up to eight distinct functionalities (by introducing eight functionalised terephthalate linkers during synthesis) in one phase, resulting, in the case of a MOF linked by three different terephthalate derivatives, in up to 400 % better CO_2_ selectivity (vs. CO) than their separate MOF‐5 counterparts.7c

MOFs have been proposed as an attractive alternative to mitigate drawbacks that other drug delivery systems (DDSs) face,^5^, ^8^ as they can exhibit low toxicity, good clearance, high drug loadings, and are easy to functionalise, yet examples of MTV‐MOFs for drug delivery applications are still limited.^9^ The coordination modulation (CM) protocol^10^—in which monotopic ligands (modulators) compete with the MOF linkers for metal cluster coordination sites during synthesis—has been widely studied to control physical properties such as size,^11^ crystallinity, colloidal dispersion,^12^ stability and porosity (through defect chemistry),^13^ while we have recently shown that it can be used to control MOF surface chemistry and functionality.^14^ Additionally, we have introduced the concept of in situ defect drug loading in MOFs, showing that using dichloroacetic acid as a modulator during assembly of UiO‐66‐type MOFs of ideal formula [Zr_6_O_4_(OH)_4_(L)_6_]_*n*_, results in highly porous, well‐dispersed nanoparticles with a high incorporation of dichloroacetate (**DCA**) as a defect‐compensating ligand.^15^ The protocol is amenable to surface functionalisation, both during synthesis by incorporating a second modulator and through postsynthetic coating, and also allows further postsynthetic drug loading of 5‐fluorouracil (**5‐FU**) into the MOF pores,14c in both cases without major **DCA** leakage. Many clinical anticancer treatments involve a cocktail of drugs (e.g. FOLFORINOX combines **5‐FU**, leucovorin, irinotecan and oxaliplatin against metastatic pancreatic adenocarcinoma)^16^ yet examples of MOFs capable of delivering multiple drugs are relatively scarce,8d, 17 suggesting defect‐loading could be an attractive strategy for preparing multimodal chemotherapeutic formulations.

**Scheme 1:**
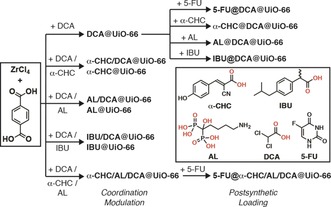
Syntheses of drug modulated MOFs and their postsynthetic drug loading. The chemical structures (coordinating groups in red) and abbreviations of the drugs are shown in the inset.

Despite the fact that multiple drugs contain metal‐binding units such as carboxylates, and the easier industrial manufacturing of producing drug‐loaded MOFs in one‐pot syntheses, examples of drugs used as modulators are still uncommon in the literature.^18^ Based on our previous work14c, 15 we sought to examine the potential for incorporating multiple drugs as modulators in one‐pot syntheses in a process we call MTV modulation (MTVM, Figure 1 b). Herein we show it is possible to introduce up to three drugs into the Zr benzene‐1,4‐dicarboxylate (BDC) MOF UiO‐66 with versatility based on the simultaneous introduction of carboxylate and phosphonate‐containing moieties. Our MTVM protocol provides particle size control (ca. 100 nm), while the resultant multi‐drug MTVM MOFs retain their porosity, which is used to postsynthetically store a fourth drug (Scheme 1 and SI, Section S2). We believe that the MTVM protocols could be applied to a wide range of modulators for alternative enhanced applications such as catalysis and gas adsorption/separation.

## Results and Discussion

Previously, we and others have shown that UiO‐66 is biocompatible,8c, 19 while the cytotoxicity of **DCA**‐loaded MOFs is dependent on surface chemistry,14c, 14d and incorporation of a second drug can dramatically enhance overall cytotoxicity.^15^ As well as **DCA**, we have chosen α‐cyano‐4‐hydroxycinnamic acid (**α‐CHC**), a molecule recently proposed as an anticancer agent, due to the relatively low p*K*_a_ of its carboxylate unit (2.2), comparable with the one of **DCA** (1.4). As a proof‐of‐concept of the effect of the p*K*_a_ in the drug‐modulated synthesis, we have also studied modulation with ibuprofen (**IBU**), due to its similar structure to **α‐CHC** and the higher p*K*_a_ of its carboxylate (4.9). Alendronate (**AL**)^20^ is an anticancer drug which contains two phosphonates—which are expected to show higher affinity for Zr than carboxylates—with a first p*K*_a_ of 2.4. **5‐FU** has been chosen as a drug to be loaded postsynthetically, as it does not contain a metal‐binding unit.

At first, we postsynthetically loaded our previously reported **DCA@UiO‐66**,^15^ prepared by coordination modulation, separately with **5‐FU**, **α‐CHC**, **IBU**, and **AL** (Scheme 1). While **5‐FU** loading resulted in ca. 1.2 % (w/w) incorporation with minimal **DCA** leakage, as determined by ICP‐OES, postsynthetic loading of drugs containing metal‐binding units resulted in partial or total displacement of **DCA** (Table S1), as determined by ^1^H NMR spectroscopic analysis of acid‐digested samples, suggesting their loading occurs through attachment to the Zr positions (subsequently detaching **DCA**) rather than pore storage (see SI, Section S3.1 for full characterisation). **AL** loading resulted in a complete structural change, likely due to the affinity of its phosphonate groups for Zr inducing breakdown,^21^ again confirming the competing coordination of the drugs. As such, this postsynthetic process was not considered viable for multiple drug loading.

Modulation with a single drug, either **α‐CHC**, **IBU** or **AL**, results in crystalline MOFs with the UiO topology,^22^ as confirmed by PXRD (see SI, Section S3.2 for full characterisation), although in the case of **AL@UiO‐66**, new Bragg peaks could be observed in the PXRD pattern, suggesting some incorporation of the bis‐phosphonate **AL** as a linker and subsequent minor structural alteration. ^1^H NMR spectra of the acid digested samples confirmed the presence of modulators, and their content is tabulated in Table S2. However, scanning electron microscopy (SEM) showed that all the individual drug‐modulated MOFs consisted of aggregates of various sizes and shapes, and hence could not be used for biomedical application, as monodisperse colloidally stable samples are imperative for drug delivery.^23^

**Figure 2:**
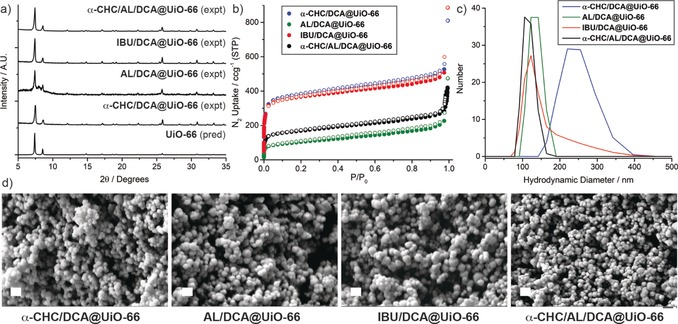
Characterisation of the dual and triple drug‐loaded MTVM UiO‐66 samples. a) Stacked partial PXRD patterns compared to that predicted for UiO‐66, confirming formation of the UiO‐66 topology. b) N_2_ uptake isotherms (77 K, filled symbols adsorption, empty symbols desorption) confirming retention of porosity to varying degrees. c) Dynamic light scattering (0.1 mg mL^−1^ in PBS 10X, pH 7.4) showing minimal aggregation. d) SEM images confirming formation of approximately spherical nanoparticles around 100–150 nm in diameter. All scale bars are 200 nm.

Adding **DCA** as a co‐modulator of each MTVM synthesis overcomes the sample aggregation issue, resulting in crystalline MOFs with Bragg reflection peaks characteristic of the UiO‐66 topology in all cases (Figure 2 a), although **AL/DCA@UiO‐66** had poorer crystallinity with some additional reflections again suggestive of structural change. The modulator content (see SI, Table S3) can be measured in mol % compared to the linker BDC by ^1^H NMR spectroscopic digestion; while this allows an assessment of relative incorporation of drugs (and thus defectivity) it cannot yield weight percentage loading due to the complexity of defect formation. *Molar* loading values are in concordance with the modulators’ expected affinities for Zr. **α‐CHC/DCA@UiO‐66** contained 6.7 mol % of **α‐CHC** (ca. 1 **α‐CHC** per 12 BDC), and ≈35 mol % of **DCA** (ca. 1 **DCA** per 3 BDC) whilst **IBU/DCA@UiO‐66** showed a smaller degree of incorporation of **IBU** (2.9 mol % compared to BDC) due to its higher p*K*_a_,13a and similarly ≈37 mol % of **DCA**. Alendronate had the highest incorporation, again likely representative of its ditopicity and the Zr‐phosphonate affinity,^21^ as **AL/DCA@UiO‐66** contained 38.1 mol % (ca. 1 **AL** per 2.5 BDC), suggesting its possible role as a linker, and 18.8 mol % of **DCA** (ca. 1 **DCA** per 3.5 BDC). Thermogravimetric analysis of samples showed complex overlapping thermal degradation processes, often with lowered thermal stabilities compared to pristine UiO‐66, confirming that the drugs are anchored to the MOF structures but not allowing quantification of drug loading using this technique.

The samples were highly porous (Figure 2 b), confirming, together with TGA and FT‐IR spectroscopy (See S3.3 for full characterisation) the attachment of the modulators to the Zr positions. In fact, as a general trend for the **DCA**‐modulated **IBU** and **α‐CHC** samples, porosity was notably enhanced (S_BET_ increased from ≈1000–1100 m^2^ g^−1^ to ≈1500 m^2^ g^−1^) compared to the single‐drug modulated analogues, whereas in the case of **AL/DCA@UiO‐66** the porosity of the sample is reduced (S_BET_ decreased from 1245 m^2^ g^−1^ to 369 m^2^ g^−1^) upon **DCA** addition, likely as a consequence of the much higher incorporation of alendronate and poorer overall crystallinity indicated by PXRD. Unfortunately, due to the defective nature of the samples, in which modulators replace the linkers in the structure, exact structural determination is troublesome. The MOFs were easily dispersed in phosphate‐buffered saline (PBS 10X, pH 7.4), with dynamic light scattering (DLS) measurements (Figure 2 c) in great agreement with the particle size determined by SEM (*ca*. 100 nm, Figure 2 d), confirming, together with our previous results,14c, 15 that **DCA** co‐modulation serves as a size control protocol, that also enhances the colloidal dispersion of the samples.^12^

This versatile synthetic protocol to introduce drugs during synthesis using **DCA** as a co‐modulator was further explored to introduce three drugs (**α‐CHC**, **AL** and **DCA**; **IBU** was not further investigated as it is not an anticancer drug) during synthesis into a single MTVM MOF structure to give **α‐CHC/AL/DCA@UiO‐66**. As the Cl (**DCA**) and P (**AL**) content of the samples can be determined by ICP‐OES, and the **α‐CHC** content by UV/Vis spectroscopy of digested samples, the corresponding drug loadings by *mass* are tabulated in Table 1, as these values are more relevant for subsequent cytotoxicity experiments.

**Table 1:** Drug loadings by mass of the MTVM MOFs examined for cytoxicity.

MOF	Drug loading [%] (w/w)			
	DCA^[a]^	α‐CHC^[b]^	AL^[c]^	5‐FU^[d]^
α‐CHC/DCA@UiO‐66	11.0 %	2.0 %	–	–
AL/DCA@UiO‐66	3.7 %	–	27.5 %	–
5‐FU@DCA@UiO‐66	8.0 %	–	–	1.2 %
α‐CHC/AL/DCA@UiO‐66	3.2 %	2.0 %	21.6 %	–
5‐FU@α‐CHC/AL/DCA@UiO‐66	3.1 %	1.6 %	21.3 %	1.6 %

[a] Determined by ICP‐OES (Cl content). [b] Determined by UV/Vis titration. [c] Determined by ICP‐OES (P content). [d] Determined by ICP‐OES (F content).

Despite the significant incorporation of the multiple drugs into UiO‐66 structure (Table 1), **α‐CHC/AL/DCA@UiO‐66** was found to be highly crystalline (Figure 2 a, see SI, Section S3.4 for full characterisation). The sample again maintained a high **AL** loading of 21.6 % (w/w), as well as 3.2 % (w/w) loading of **DCA**, and 2.0 % (w/w) loading of **α‐CHC**. Assuming binding of monoanions at defects, this corresponds to a **α‐CHC**:**DCA:AL** molecular ratio of 1:2.3:8.1, in great agreement with reports showing the high affinity of **AL** for UiO‐66 Zr clusters^20^ and of the role of p*K*_a_ in defect binding. TGA, FT‐IR spectroscopy and N_2_ adsorption and desorption measurements confirmed the drugs to be attached to the Zr positions, as **α‐CHC/AL/DCA@UiO‐66** has a surface area of 634 m^2^ g^−1^ despite containing over 25 % (w/w) of drugs in its structure (Figure 2 b). The lower porosity could also be attributed to the high incorporation of **AL**, as seen with the analogous **AL/DCA@UiO‐66** sample. A comparison of the drug incorporation ratios with the surface areas and pore volumes, indicative of the level of defectivity and/or structural change, is given in the Supporting Information, Table S5.

Once again, size control was achieved resulting in nanoparticles of ca. 125–150 nm (Figure 2 d) and the material was well‐dispersed in PBS (Figure 2 c). The porosity was further used to postsynthetically load **5‐FU**, resulting in a DDS, **5‐FU@α‐CHC/AL/DCA@UiO‐66**, containing a cocktail of four anticancer drugs. ICP‐OES determined 1.6 % (w/w) **5‐FU** content with negligible leakage of the modulator drugs.

**Figure 3:**
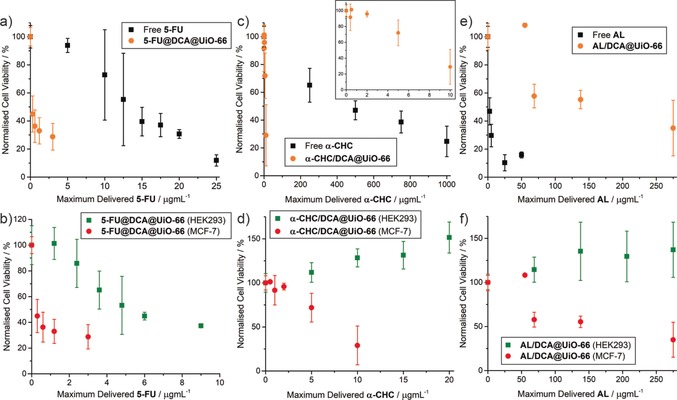
MTS proliferation assays of the dual‐loaded MOFs towards MCF‐7 cancer cells and HEK293 kidney cells compared to the free drug. Values are normalised to the maximum possible dose of the most cytotoxic drug. a) Comparison of MCF‐7 cytotoxicity of **5‐FU@DCA@UiO‐66** with free **5‐FU**. b) Comparison of cytotoxicity of **5‐FU@DCA@UiO‐66** towards MCF‐7 *vs*. HEK293. c) Comparison of MCF‐7 cytotoxicity of **α‐CHC/DCA@UiO‐66** with free **α‐CHC**. d) Comparison of cytotoxicity of **α‐CHC/DCA@UiO‐66** towards MCF‐7 *vs*. HEK293. e) Comparison of MCF‐7 cytotoxicity of **AL/DCA@UiO‐66** with free **AL**. f) Comparison of cytotoxicity of **AL/DCA@UiO‐66** towards MCF‐7 vs. HEK293. Error bars represent the standard deviation of the mean value of three independent assays.

The MTVM drug‐loaded MOFs were tested for anticancer selectivity against MCF‐7 breast cancer and HEK293 kidney cells by the MTS assay (See SI, Section S4) after 72 hours of incubation and compared to the effect of the free drugs, which allows comparison with our previous work.14c The similar size of all MOFs (ca. 90–150 nm) allows comparison of therapeutic efficacy without concerns over major particle size influence. For both cell lines, there is approximately an order of magnitude difference in the cytotoxicities of the free drugs, with IC_50_ values in the order **AL** < **5‐FU** < **α‐CHC** < **DCA** (Table S6), and each drug shows a small selectivity for cytotoxicity towards the MCF‐7 cancer cell line compared to HEK293. In order to delineate the effects of the MOF delivery vehicle on the efficacies of the different drugs individually and in tandem, cytotoxicities of the MOFs containing either **AL**, **5‐FU** or **α‐CHC** alongside **DCA** are first assessed (Figure 3).

In great agreement with our previous studies of ultra‐small UiO MOFs for dual delivery of **5‐FU** and **DCA**,^15^ **5‐FU@DCA@UiO‐66** was profoundly more cytotoxic than free **5‐FU** towards MCF‐7 cells (Figure 3 a), with an IC_50_ dose normalised to **5‐FU** over 35 times lower than the free drug, decreasing cell proliferation to values to ca. 45 % after treatment with 25 μg mL^−1^ of MOF for 72 hours (IC_50_ values for free drugs are listed in Table S6, and are tabulated for all MOF samples, normalised to the varying components, in Tables S7 and S8). Its cytotoxic effect towards HEK293 was ca. 21 times that of the free drug, so whilst toxicity also increased towards non‐cancerous cells, the overall selectivity compared to the free drug improved nearly two‐fold (Figure 3 b). It has previously been reported that **DCA** enhances the anticancer activity and selectivity of certain drugs, including **5‐FU**,^24^ however, doses at least ten times higher than those used here are usually needed to generate a synergistic effect when the drugs are not loaded into a DDS.^15^

A pronounced enhancement of the therapeutic effect of **α‐CHC** towards MCF‐7 cancer cells was found for **α‐CHC/DCA@UiO‐66** (Figure 3 c). Despite both **α‐CHC** and **DCA** having IC_50_ doses against MCF‐7 cells in the millimolar range, the IC_50_ of the drug‐loaded MOF towards MCF‐7 corresponds to a maximum delivered dose of **α‐CHC** that is 27 times lower than that for the free drug and to a delivered dose of **DCA** 111 times lower than the IC_50_ of the free drug. This dramatic enhancement is not observed for the HEK293 cells, where the MOF is biocompatible up to 1 mg mL^−1^ of MOF (Figure 3 d). Taken together, this again corresponds to an increase in cytotoxicity and selectivity towards MCF‐7 versus HEK293.

In contrast, a decrease in the therapeutic effect of alendronate towards MCF‐7 breast cancer cells was found for **AL/DCA@UiO‐66** (Figure 3 e). The increase of the IC_50_ of **AL** upon delivery from **AL/DCA@UiO‐66** corresponds to a maximum delivered concentration 7 times higher than that of the free drug. The bis‐phosphonate structure of **AL** is likely to be strongly adhered to the Zr positions of the MOF, and so incomplete release may attenuate cytotoxicity and raise the possibility of slower, controlled release to mitigate side effects in vivo. It is important to note that the MOF was not cytotoxic towards HEK293 cells up to 1 mg mL^−1^ of MOF (Figure 3 f), a formulation loaded with an **AL** content 49 times higher than the IC_50_ of the free drug, meaning that although there is no enhancement in the therapeutic activity of the drug towards MCF‐7 cancer cells, there is again a remarkable increase in its selectivity.

**Figure 4:**
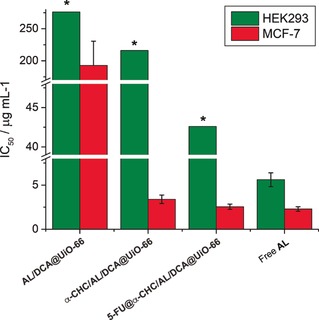
Comparison of IC_50_ values, normalised to **AL**, for various formulations against HEK293 and MCF‐7 cells. Measurements marked with an asterix (*) represent the maximum concentration assessed where cell proliferation remained >90 % and so IC_50_ values (and error bars) cannot be calculated. In all formulations, selectivity of anticancer cytotoxicity is enhanced when **AL** is incorporated in UiO‐66.

For the triple‐drug formulations, **α‐CHC/AL/DCA@UiO‐66** again increased the IC_50_ against MCF‐7 cells when normalised to **AL** content, to a dose around 1.5 times higher than that for the free drug (Figure 4). Incubation with 25 μg mL^−1^ of **α‐CHC/AL/DCA@UiO‐66** decreased MCF‐7 cell proliferation to 34±6 %, further decreasing to 18±11 % upon treatment with a concentration of 250 μg mL^−1^ of MOF. Evaluating the MOFs by the loading of their most cytotoxic drug component, **AL**, suggests **α‐CHC/AL/DCA@UiO‐66** exhibits around 57 times the cytotoxicity towards MCF‐7 compared to **AL/DCA@UiO‐66**, and is also significantly more cytotoxic than comparable doses of **α‐CHC/DCA@UiO‐66**.

The effect towards HEK293 cells, however was remarkably different. **α‐CHC/AL/DCA@UiO‐66** was found to be biocompatible up to 1 mg mL^−1^ of MOF (maximum **AL** delivered dose 38 times higher than the IC_50_ of the free drug); the selectivity of cytotoxicity against MCF‐7 versus HEK293 is again enhanced compared to **AL** alone.

Loading the MTVM MOF with a fourth drug, **5‐FU**, resulted in a further enhancement of the IC_50_ dose of **5‐FU@α‐CHC/AL/DCA@UiO‐66** towards MCF‐7 cells, with the IC_50_ normalised to **AL** content just 10 % higher than the free drug. Biocompatibility with HEK293 cells was maintained up to 0.2 mg mL^−1^ MOF incubation MOF (maximum **AL** delivered dose 7.5 times higher than the IC_50_ of the free drug), suggesting that multimodal drug delivery can synergistically enhance anticancer activity while conserving the biocompatibility towards HEK293 cells at the concentrations studied.

## Conclusion

On the whole, we have demonstrated that different drugs, containing either carboxylates or phosphonates as metal‐binding units, can be introduced to the synthesis of UiO‐66—and potentially any other Zr MOF of the UiO family—as simultaneous modulators. The drugs become attached to the Zr clusters of the resulting MOF, which has been found to be related to both the p*K*_a_ of the metal binding unit (the lower the p*K*_a_, the higher the incorporation) and its chemical functionality (phosphonates have a higher affinity for Zr than carboxylates and are incorporated more). As the drug‐modulators are attached as defects rather than pore‐loaded, the resultant MTVM MOFs are highly porous, and we have used their porosity to postsynthetically load **5‐FU**, ultimately resulting in four drugs incorporated in significant quantities into a single nanovector. We have shown that adding DCA to the drug modulated syntheses also offers size control, resulting in nanoparticles of ca. 100 nm that are well‐dispersed in PBS (10X), which enables the comparison of their cytotoxicity without concerns over size effects. The anticancer therapeutic activity of the double drug combinations towards MCF‐7 breast cancer cells is highly increased for **5‐FU@DCA@UiO‐66** and **α‐CHC/DCA@UiO‐66** compared to the free drugs, whereas the MOFs are biocompatible to HEK293 kidney cells even at high doses, enhancing selectivity. Although the therapeutic activity of **AL** when loaded into the MOFs is reduced in all cases compared to the free drug, likely as it is not fully released from the core of the MOF, increases in cytotoxicity towards MCF‐7 cells are noted for treble and quadruple drug formulations as the drug cocktails become more complex. Additionally, a drastic increase in selectivity towards cancer cells is achieved across the formulations; **5‐FU@α‐CHC/AL/DCA@UiO‐66** maintains the cytotoxicity of free **AL** towards MCF‐7 cells yet is considerably more biocompatible towards HEK293 cells than free **AL**, suggesting that drug delivery using MOFs as DDSs could overcome the unwanted cytotoxic issues of some drugs.

Our MTVM protocols are highly versatile and reproducible (both carboxylates and phosphonates can be introduced simultaneously into one single phase during synthesis), and could be applied to almost any drug containing potential metal‐binding units (e.g. doxorobucin or paclitaxel) and to other MOF systems, opening a broad range of possibilities and combinations to design novel drug loaded MOFs. While we have used biologically relevant molecules as modulators in this study, we are convinced that the MTVM protocol can be used to introduce modulators to enhance other applications through cooperative effects, such as preferential gas capture or heterogeneous catalysis, and also to tune the host‐guest interactions of the frameworks through pore environment control to favour the uptake of certain gases (gas uptake/separation), chelating units (water treatment/ pollutant removal) and cooperative units for catalysis, among many other possibilities.

## Conflict of interest

The authors declare no conflict of interest.

## Supporting information

As a service to our authors and readers, this journal provides supporting information supplied by the authors. Such materials are peer reviewed and may be re‐organized for online delivery, but are not copy‐edited or typeset. Technical support issues arising from supporting information (other than missing files) should be addressed to the authors.

Supplementary
