# Supplementary material for: Multivariate Modulation of the Zr MOF UiO‐66 for Defect‐Controlled Combination Anticancer Drug Delivery
Source: Angew Chem Int Ed Engl. 2020 Feb 4;59(13):5211–7. doi: 10.1002/anie.201915848 (PMC7154787; doi:10.1002/anie.201915848)
Supplement: Supplementary file 1 — Supplementary [file ANIE-59-5211-s001.pdf]

Angewandte Chemie
Eine Zeitschrift der Gesellschaft Deutscher Chemiker

Supporting Information

Multivariate Modulation of the Zr MOF UiO-66 for Defect-Controlled Combination Anticancer Drug Delivery

*Isabel Abánades Lázaro, Connor J. R. Wells, and Ross S. Forgan**

anie_201915848_sm_miscellaneous_information.pdf

SUPPORTING INFORMATION

TABLE OF CONTENTS:

**S1. General Experimental Remarks**	**S2.**
**S2. Experimental**	**S4.**
S2.1. Material Synthesis	**S4.**
S2.2. *In Vitro* Cytotoxicity Assays and Protocols	**S7.**
**S3. Characterisation of Materials**	**S8.**
S3.1. Characterisation of DCA@UiO-66 Postsynthetically Loaded with Drugs	**S8.**
S3.2. Characterisation of UiO-66 Modulated with Single Drugs	**S17.**
S3.3. Characterisation of UiO-66 Modulated with Two Drugs	**S25.**
S3.4. Characterisation of UiO-66 Modulated with Three Drugs	**S41.**
**S4. Cytotoxicity Studies**	**S48.**
S4.1. *In Vitro* Cytotoxicity of Free Drugs	**S48.**
S4.2. *In Vitro* Cytotoxicity of Drug-Loaded MOFs	**S55.**
**S5. References**	**S67.**

S1. General Experimental Remarks

Powder X-Ray Diffraction (PXRD): PXRD measurements were carried out at 298 K using a PANalytical X'Pert PRO diffractometer (λ(CuKα) = 1.4505 Å) on a mounted bracket sample stage. Data were collected over the range 5–45°.

Thermogravimetric Analysis (TGA): Measurements were carried out using a TA Instruments Q500 Thermogravimetric Analyser. Measurements were collected from room temperature to 800 °C with a heating rate of 10 °C / min under an air atmosphere.

Nuclear Magnetic Resonance Spectroscopy (NMR): NMR spectra were recorded on either a Bruker AVIII 400 MHz spectrometer or a Bruker AVI 500 MHz spectrometer and referenced to residual solvent peaks.

Gas Uptake: N_2 adsorption isotherms were carried out at 77 K on a Quantachrome Autosorb iQ gas sorption analyser. Samples were degassed under vacuum at 120 °C for 20 h using the internal turbo pump. BET surface areas were calculated from the isotherms using the Micropore BET Assistant in the Quantachrome ASiQwin operating software.

Pore-Size Distribution: Pore size distributions were calculated using the N_2 at 77 K on carbon (slit pore, QSDFT, equilibrium model) calculation model within the Quantachrome ASiQwin operating software.

UV-Vis Spectroscopy: UV-vis spectra were recorded using a Shimadzu UV-1800; analysis was carried out using the software UVProve.

Electrospray Ionisation Mass Spectrometry: ESI-MS was carried out on solution samples injected into a Bruker MicroTOFq spectrometer.

Scanning Electron Microscopy (SEM): The powder samples were coated with Pd for 50 seconds using Polaron SC7640 sputter coater and imagined using a Carl Zeiss Sigma Variable Pressure Analytical SEM with Oxford Microanalysis. Particle size distribution was analysed manually using ImageJ software and calculated from a minimum of 50 nanoparticles.

Fourier Transform Infrared Spectroscopy: IR spectra of solids were collected using a Shimadzu Fourier Transform Infrared Spectrometer, FTIR-8400S, fitted with a Diamond ATR unit.

Dynamic Light Scattering: Colloidal analysis was performed by Dynamic Light Scattering (DLS) with a Zetasizer Nano ZS potential analyser equipped with Non-Invasive Backscatter optics (NIBS) and a 50 mW laser at 633 nm. Samples were sonicated for 5 minutes in a temperature controlled sonicator bath prior to analysis, and were not filtered.

S2. Experimental

S2.1. Materials and Synthesis

All reagents unless otherwise stated were obtained from commercial sources and were used without further purification. All MOF syntheses were performed at 120 °C and the resultant powders were washed with ca. 30 mL of solvent during each dispersion / centrifugation cycle. The postsynthethically loaded samples were washed with 10 mL of solvent during each cycle.

Synthesis of Dichloroacetic Acid (DCA) Modulated UiO-66 (DCA@UiO-66). DCA@UiO-66 synthesis was performed following our previously reported[S1] coordination modulation protocol: 1,4-benzenedicarboxylic acid (150 mg, 0.9 mmol) was dissolved in 10 mL of DMF, and in a separate vial, the metal precursor, zirconium chloride (210 mg, 0.9 mmol) was dissolved in 10 mL of DMF. Both solutions were sonicated until complete dissolution and mixed together. Subsequently, 10 equivalents of DCA were added to the synthesis, together with 1 equivalent of HCl. Then, the reaction mixtures were placed in the oven at 120 °C. After 24 h, the reaction mixture was cooled to room temperature and the NMOFs were collected as white powders by centrifugation and washed with DMF (30 mL) and MeOH (3 x 30 mL).

Postsynthetic Alendronate (AL) Drug Loading. Postsynthetic alendronate loading was performed by dispersing by sonication (5 minutes) 60 mg of **DCA@UiO-66** in 10 mL of drug containing solution (10 $mgmL^{-1}$ of AL in water). The dispersion was stirred at room temperature for three days. The resultant white MOF, **AL@DCA@UiO-66** was collected by centrifugation and washed with water (10 mL) and with MeOH (2 x 10 mL). The sample was dried under vacuum for 24 h prior to analysis.

Postsynthetic α-Cyano-4-Hydroxycinnamic Acid (α-CHC) Drug Loading. Postsynthetic α-CHC loading was performed by dispersing by sonication (5 minutes) 60 mg of **DCA@UiO-66** in 10 mL of drug containing solution (50 $mgmL^{-1}$ of α-CHC in methanol). The dispersion was stirred at room temperature for three days. The resultant yellow MOF, **α-CHC@DCA@UiO-66** was collected by centrifugation and washed with MeOH (3 x 10 mL). The sample was dried under vacuum for 24 h prior to analysis.

Postsynthetic 5-Fluorouracil (5-FU) Drug Loading. Postsynthetic 5-FU loading was performed by dispersing by sonication (5 minutes) 60 mg of **DCA@UiO-66** in 10 mL of drug

containing solution (2 mgmL^{-1} of 5-FU in methanol). The dispersion was stirred at room temperature for one day. The resultant MOF, **5-FU@DCA@UiO-66** was collected by centrifugation and washed with MeOH (3 x 10 mL). The sample was dried under vacuum for 24 h prior to analysis.

Postsynthetic Ibuprofen (IBU) Drug Loading. Postsynthetic IBU loading was performed by dispersing by sonication (5 minutes) 60 mg of **DCA@UiO-66** in 20 mL of drug containing solution (10 mgmL^{-1} of IBU in methanol). The dispersion was stirred at room temperature for two days. The resultant MOF, **IBU@DCA@UiO-66** was collected by centrifugation and washed with MeOH (3 x 10 mL). The sample was dried under vacuum for 24 h prior to analysis.

Synthesis of α-CHC@UiO-66 and α-CHC/DCA@UiO-66

1,4-Benzenedicarboxylic acid (150 mg, 0.9 mmol) plus one equivalent of α-CHC (170 mg, 0.9 mmol) were dissolved in 10 mL of DMF, and in a separate vial, the metal precursor, zirconium chloride (210 mg, 0.9 mmol) was dissolved in 10 mL of DMF. Both solutions were sonicated until complete dissolution and mixed together. Subsequently, when necessary, 5 equivalents of DCA were added to the synthesis. After heating to 120 °C for 24 h, the reaction mixture was cooled to room temperature and the yellow NMOFs were collected with centrifugation and washed with DMF (30 mL) and MeOH (3 x 30 mL). The sample was dried under vacuum for 24 h prior to analysis.

Synthesis of AL@UiO-66 and AL/DCA@UiO-66

1,4-Benzenedicarboxylic acid (150 mg, 0.9 mmol) plus 0.5 equivalents of alendronate (146 mg, 0.45 mmol) were dissolved in 10 mL of DMF, and in a separate vial, the metal precursor, zirconium chloride (210 mg, 0.9 mmol) was dissolved in 10 mL of DMF. Note that alendronate does not dissolve at room temperature but does upon reaction mixture heating. Both solutions were sonicated and mixed together. Subsequently, when necessary, 5 equivalents of DCA were added to the synthesis. After heating to 120 °C for 24 h, the reaction mixture was cooled to room temperature and the NMOFs were collected with centrifugation and washed with DMF (30 mL) and MeOH (3 x 30 mL). The sample was dried under vacuum for 24 h prior to analysis.

Synthesis of IBU@UiO-66 and IBU/DCA@UiO-66

1,4-Benzenedicarboxylic acid (150 mg, 0.9 mmol) plus one equivalent of ibuprofen (185 mg, 0.9 mmol) were dissolved in 10 mL of DMF, and in a separate vial, the metal precursor, zirconium chloride (210 mg, 0.9 mmol) was dissolved in 10 mL of DMF. Both solutions were

sonicated until complete dissolution and mixed together. Subsequently, when necessary, 5 equivalents of DCA were added to the synthesis. After heating to 120 °C for 24 h, the reaction mixture was cooled to room temperature and the NMOFs were collected with centrifugation and washed with DMF (30 mL) and MeOH (3 x 30 mL). The sample was dried under vacuum for 24 h prior to analysis.

Synthesis of α-CHC/AL/DCA@UiO-66

1,4-Benzenedicarboxylic acid (75 mg, 0.45 mmol) plus one equivalent of α-CHC (85 mg, 0.45 mmol) and 0.5 equivalents of alendronate (73 mg, 0.225 mmol) were dissolved in 5 mL of DMF, and in a separate vial, the metal precursor, zirconium chloride (105 mg, 0.45 mmol) was dissolved in 10 mL of DMF. Both solutions were sonicated until complete dissolution and mixed together. Subsequently, 5 equivalents of DCA were added to the synthesis. After heating to 120 °C 24 h, the reaction mixture was cooled to room temperature and the yellow NMOFs were collected with centrifugation and washed with DMF (2 x 30 mL) and EtOH (3 x 30 mL). The sample was dried under vacuum for 24 h prior to analysis.

Postsynthetic 5-FU loading

Postsynthetic 5-FU loading of **α-CHC/AL/DCA@UiO-66** was performed by sonicating the sample in 20 mL of a solution of 5-FU in EtOH (1 $mgmL^{-1}$) during 5 minutes followed by stirring at room temperature during 24 h. The sample, **5FU@α-CHC/AL/DCA@UiO-66**, was collected by centrifugation and washed with ethanol (10 mL). The sample was dried under vacuum for 24 h prior to analysis.

Dynamic Light Scattering (DLS) Measurements. The particle size, aggregation and colloidal dispersion were measured by dynamic light scattering (DLS) in phosphate buffered saline (PBS). In a scintillation vial, dispersions of the samples with a concentration of 0.25 $mgmL^{-1}$ were prepared by sonication in a temperature controlled sonicator bath over 5 minutes prior to measurement. Each measurement consisted of 3 separate records, with a waiting time of 5 seconds between each. Each recording consisted of 14 runs, and no stirring was provided during the course of the experiment.

Drug loading calculations from ICP. Alendronate loadings were calculated based on the P content of the samples, 5-FU from the F content, DCA from the Cl content, in turn. In all cases loading values were calculated after correction with baseline P, F and Cl values obtained for a control sample of UiO-66 synthesised using AcOH as a modulator. This is particularly important for calibrating against residual Cl content from the $ZrCl_4$ starting

materials, although minimum residual chloride was observed in controls, which is consistent with our previous work on DCA-loaded MOFs.[S1]

α-CHC loading calculations from UV-Vis spectroscopy. An accurately known mass of MOF (ca. 3 mg) was dispersed by sonication (5 minutes) in 2 mL of PBS 10x (pH 7.4) and stirred at room temperature for 72 h to ensure total drug release through MOF degradation. The drug content of the supernatant was calculated by UV-Vis spectroscopy against a previously calculated calibration curve based on α-CHC absorbance at λ = 337 nm.

S2.2. *In Vitro* Cytotoxicity Assays and Protocols

MCF-7 breast cancer cells and **HEK293 human embryonic kidney cells** were maintained at 37 ºC with 5% CO_2 in high rich glucose (4500 mgL^{-1}) Dulbecco's modified Eagle's Medium (DMEM) with phenol red supplemented with 10% (*v/v*) Fetal Bovine Serum (FBS), 2 mM L-glutamine, 100 unitsmL^{-1} penicillin and 100 μgmL^{-1} streptomycin. This was named complete DMEM (cDMEM). The cells were passaged twice a week (at 75-80% of confluence) at a density of 2.8 x 10^4 cellcm^{-2}.

MTS Assay. To measure cell proliferation of MCF-7 and HEK293 the (MTS, Promega, UK) reduction assay, based on the cleavage of 3-(4,5-dimethylthiazol-2-yl)-5-(3-carboxymethoxyphenyl)-2-(4-sulfophenyl)-2H-tetrazolium salt was used. Prior to incubation with cells, the MOFs were further activated in the oven at 120 °C overnight in order to remove any residual solvent. The day before the experiment, cells were seeded into a 96 well plate at a density of 10 x 10^3 cells per well (100 μL). Prior to the treatments, cells were washed twice with PBS twice. The MOFs were suspended in cDMEM by sonication at different concentrations, added to the cells and incubated – with 5 replicates for each MOF concentration and 8 replicates for media without cells and for untreated cells – for 72 h at 37 ºC with 5% CO_2. To measure the toxicity, the cells were washed one time with phosphate buffered saline (PBS), the media was replaced with 100 μL of fresh culture media containing 20 μL of MTS/phenazinemethosulfate (in a proportion 20:1) solution, and the plate was incubated for 1 h at 37 ºC with 5% CO_2. The plates were read at 490 nm by UV/vis spectrophotometry. Each cytotoxic study was performed independently at least twice (see figure captions for specifics). Statistical analysis and IC_{50} dose calculations were performed using prism software.

S3. Characterisation of Materials

S3.1. Characterisation of DCA@UiO-66 Postsynthetically Loaded with Drugs

The postsynthetic loading of drugs into DCA@UiO-66 was assessed by PXRD, to examine structural integrity, while thermogravimetric analysis (TGA) and ^{1}H NMR spectra of acid digested samples were used to assess drug uptake.

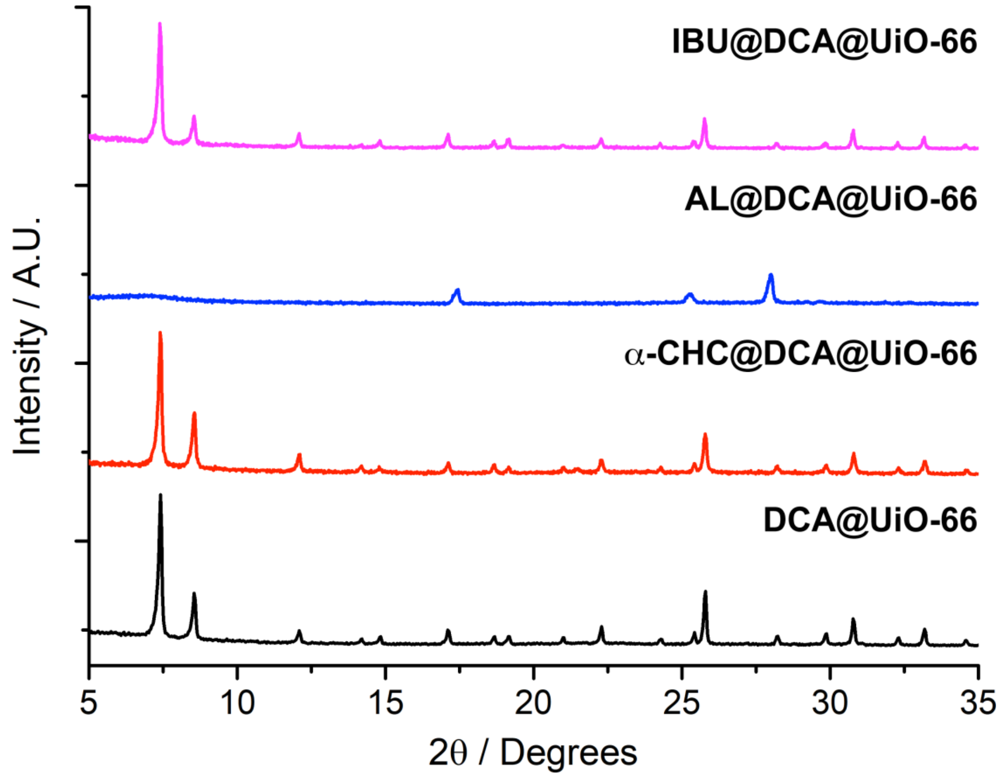

Figure S1. Stacked PXRD patterns of the postsynthetically drug-loaded **DCA@UiO-66** samples showing structural integrity for all samples apart from **AL@DCA@UiO-66**, which shows a new phase.

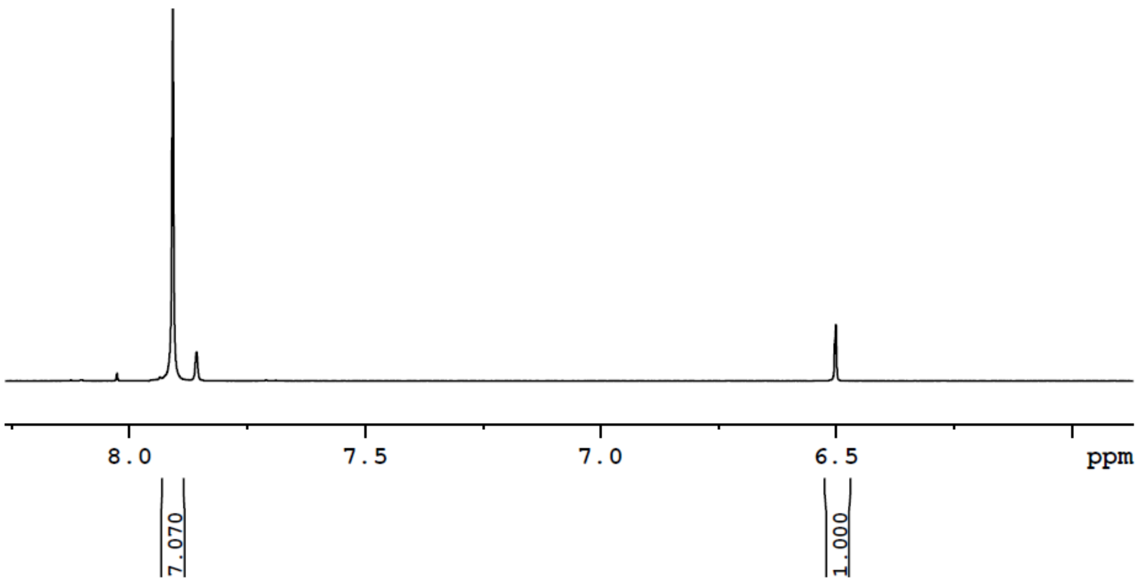

Figure S2. ^{1}H NMR spectrum in acidified DMSO-d_6 of digested **DCA@UiO-66** showing DCA presence (signal at δ = 6.5 ppm, 36 mol% compared to BDC, ca. 1 DCA per 3 BDC). The presence of formate δ = 7.7 ppm) from DMF hydrolysis during synthesis can also be observed.

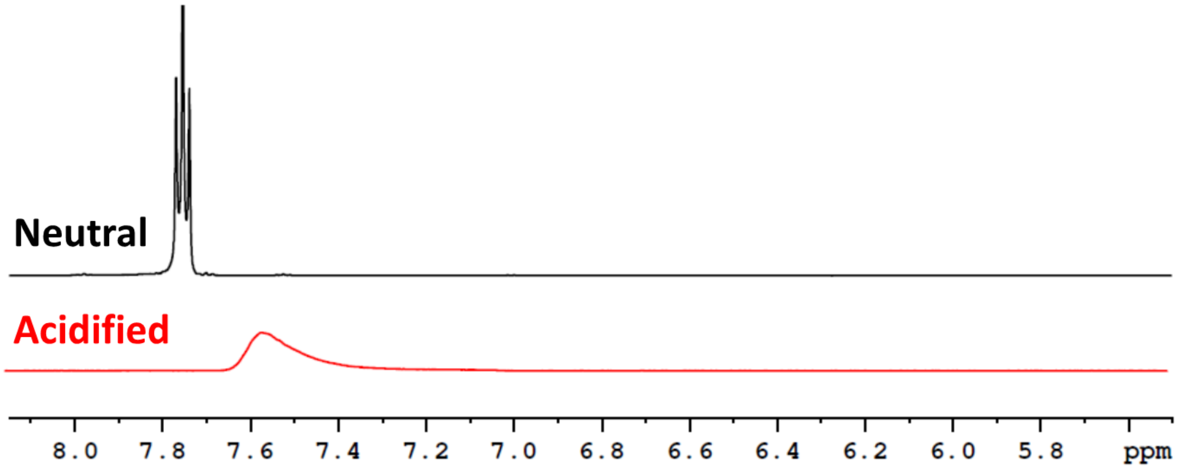

Figure S3. ^{1}H NMR spectra of 5-FU in DMSO-d_6 and acidified DMSO-d_6 showing shifting and changes in multiplicity of the resonance at ca. δ = 7.8 ppm).

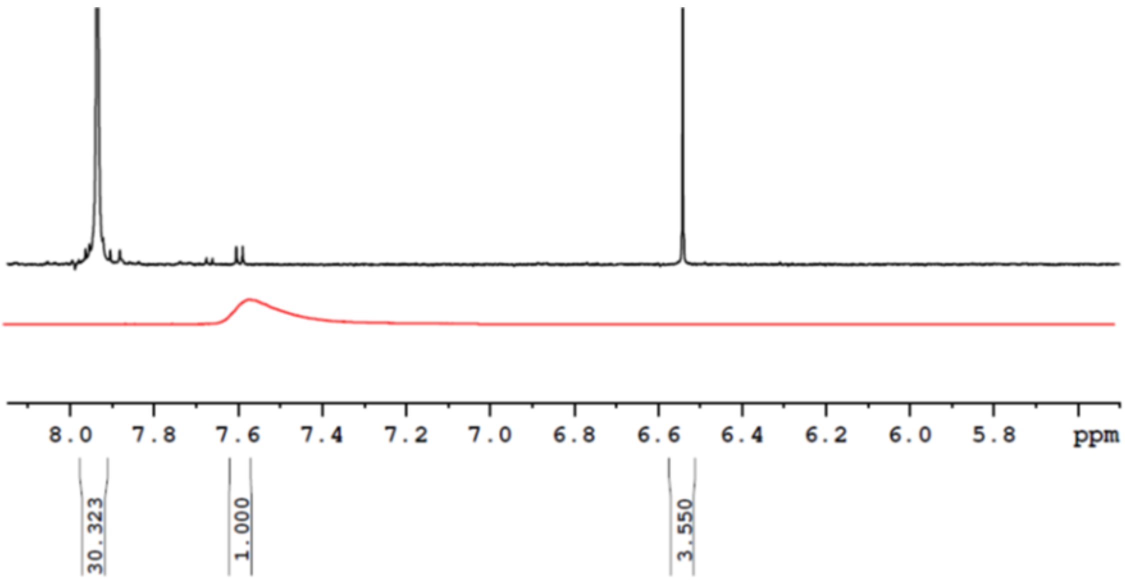

Figure S4. ^{1}H NMR spectrum in acidified DMSO-d_6 of **5-FU@DCA@UiO-66** (5-FU postsynthetically loaded into **DCA@UiO-66**) showing 5-FU incorporation (ca. 11.7 mol% compared to BDC) and DCA presence (ca. 32 mol%, ca. 1 DCA per 3 BDC), confirming DCA is not lost during 5-FU loading.

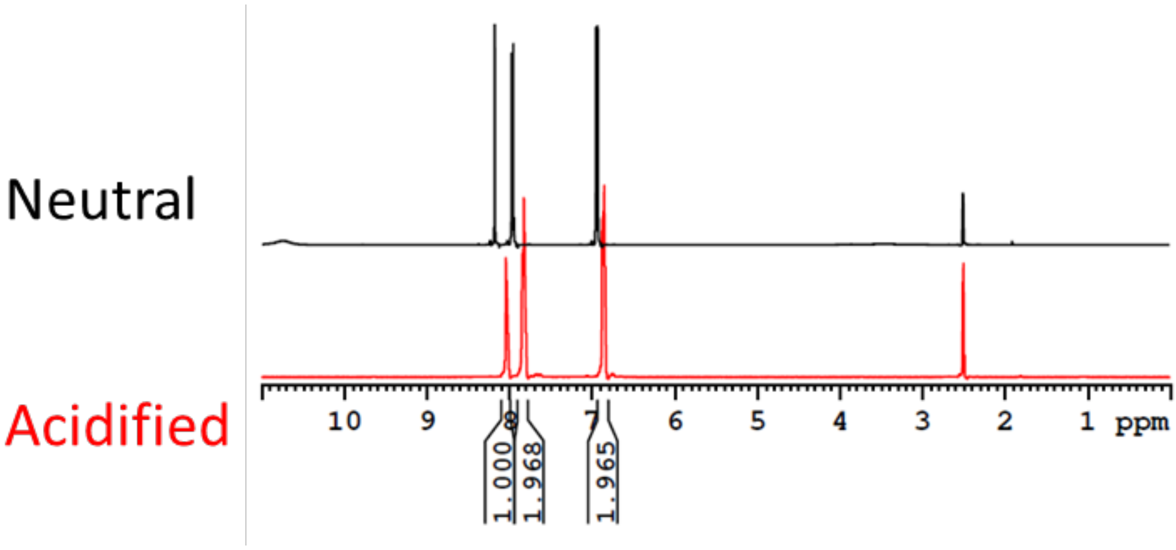

Figure S5. ^{1}H NMR spectra of α-CHC in DMSO-d_6 and acidified DMSO-d_6 showing minimal shifting of its resonances after acidification.

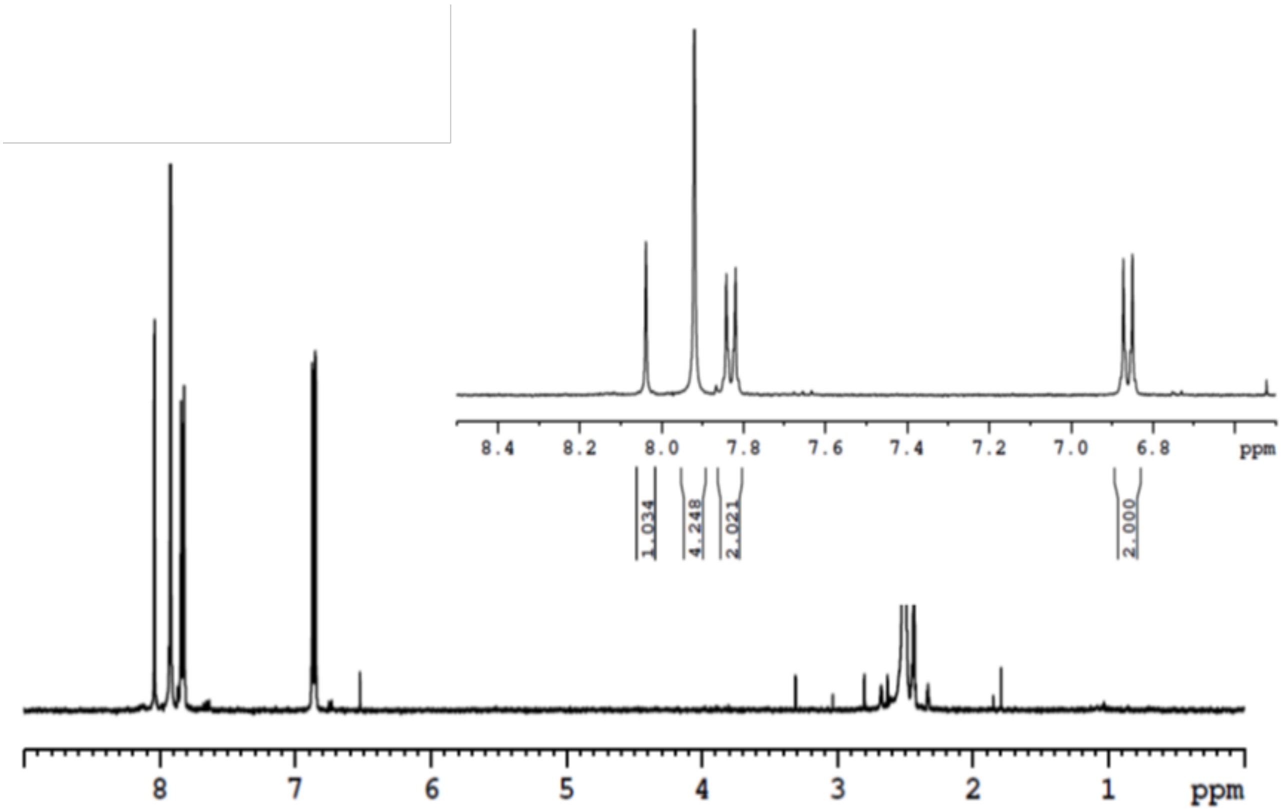

Figure S6. ^{1}H NMR spectrum of **α-CHC@DCA@UiO-66** in acidified DMSO-d_6, showing the presence of α-CHC (48.5 mol% compared to BDC, ca. 1 α-CHC per BDC) and partial displacement of DCA (from 36 mol% of precursor to 11.5 mol%) as a consequence of α-CHC attachment through its carboxylate group.

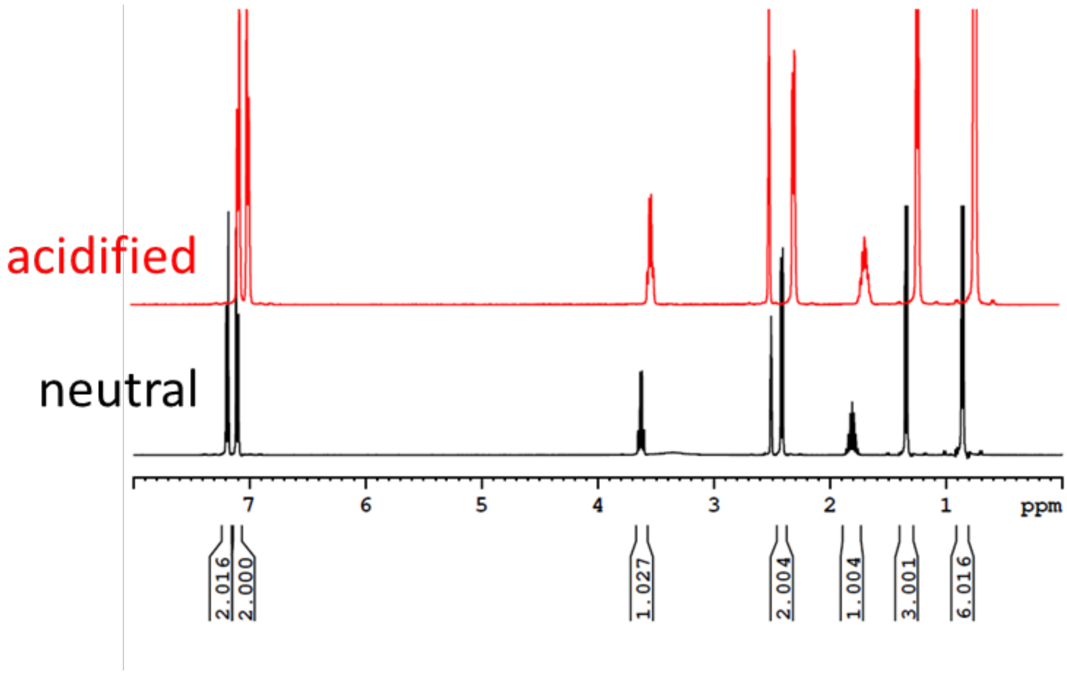

Figure S7. ^{1}H NMR spectra of IBU in DMSO-d_6 and acidified DMSO-d_6, showing the shifting of its resonances on acidification.

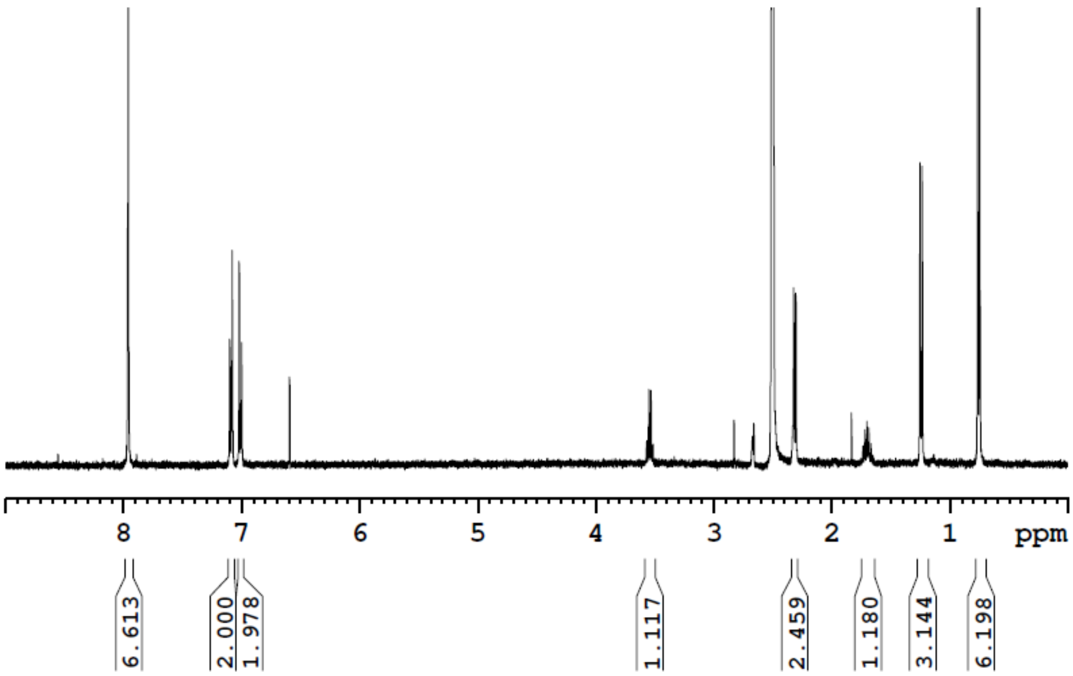

Figure S8. ^{1}H NMR of IBU@DCA@UiO-66 digested in acidified DMSO-d_6, showing the presence of IBU (37.7 mol% compared to BDC, ca. 1 IBU per 2.75 BDC) and partial displacement of DCA (from 36 mol% to 18 mol%) as a consequence of IBU attachment through its carboxylate group.

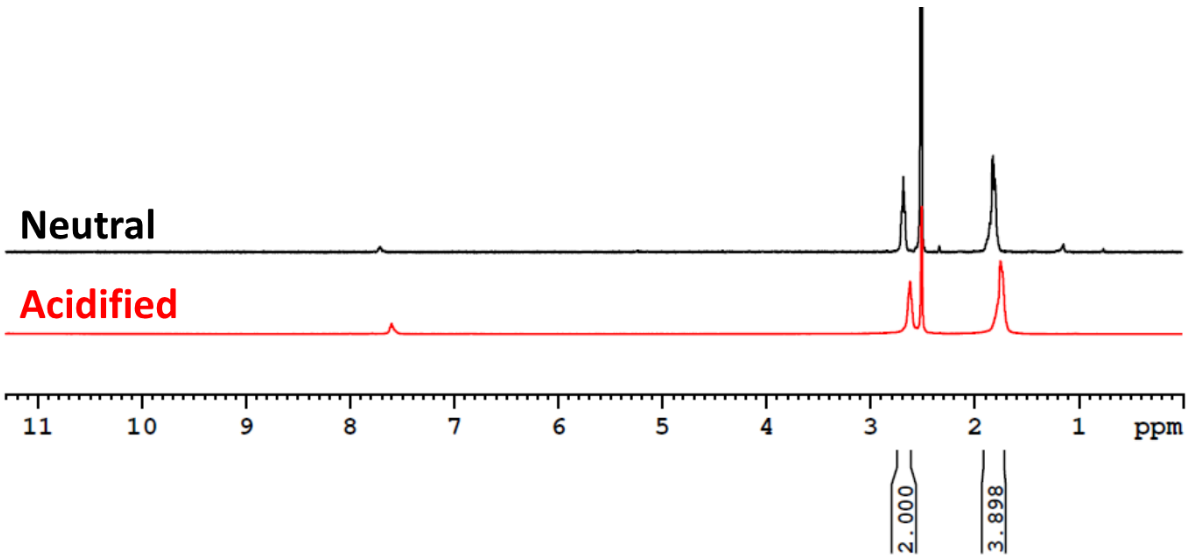

Figure S9. ^{1}H NMR spectra of AL in DMSO-d_6 and acidified DMSO-d_6, showing the shifting of its resonances on acidification.

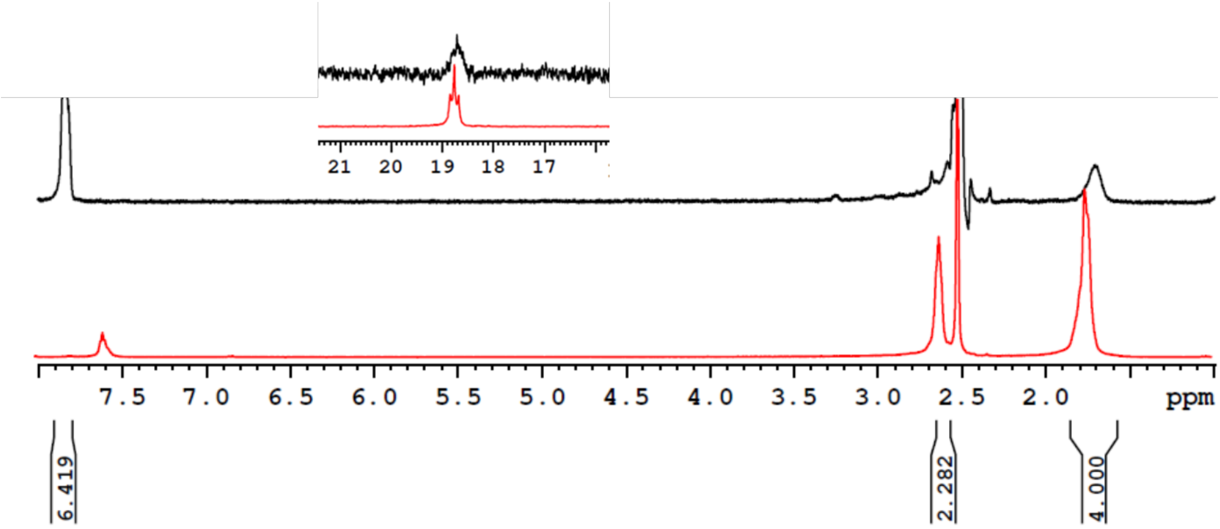

Figure S10. ^{1}H NMR spectrum (^{31}P NMR spectrum in the inset) in acidified DMSO-d_6 of **AL@DCA@UiO-66** showing the presence of AL (38 mol% compared to BDC, 1 AL per 1.5 BDC) and the total displacement of DCA as a consequence of AL attachment through its phosphate groups.

Table S1. Summary of drug loading in the postsynthetically loaded samples, assessed by ^{1}H NMR spectroscopy, compared to the **DCA@UiO-66** precursor.

**MOF**	**Drug Loading / mol % compared to BDC (^{1}H NMR)**				
	**DCA**	**5-FU**	**AL**	**α-CHC**	**IBU**
**DCA@UiO-66**	36.0%	–	–	–	–
**5-FU@DCA@UiO-66**	32.0%	11.7%	–	–	–
**AL@DCA@UiO-66**[a]	0%	–	38.0%	–	–
**α-CHC@DCA@UiO-66**	11.5%	–	–	48.5%	–
**IBU@DCA@UiO-66**	18.0%	–	–	–	37.7%

[a]PXRD confirms complete structural breakdown of this sample.

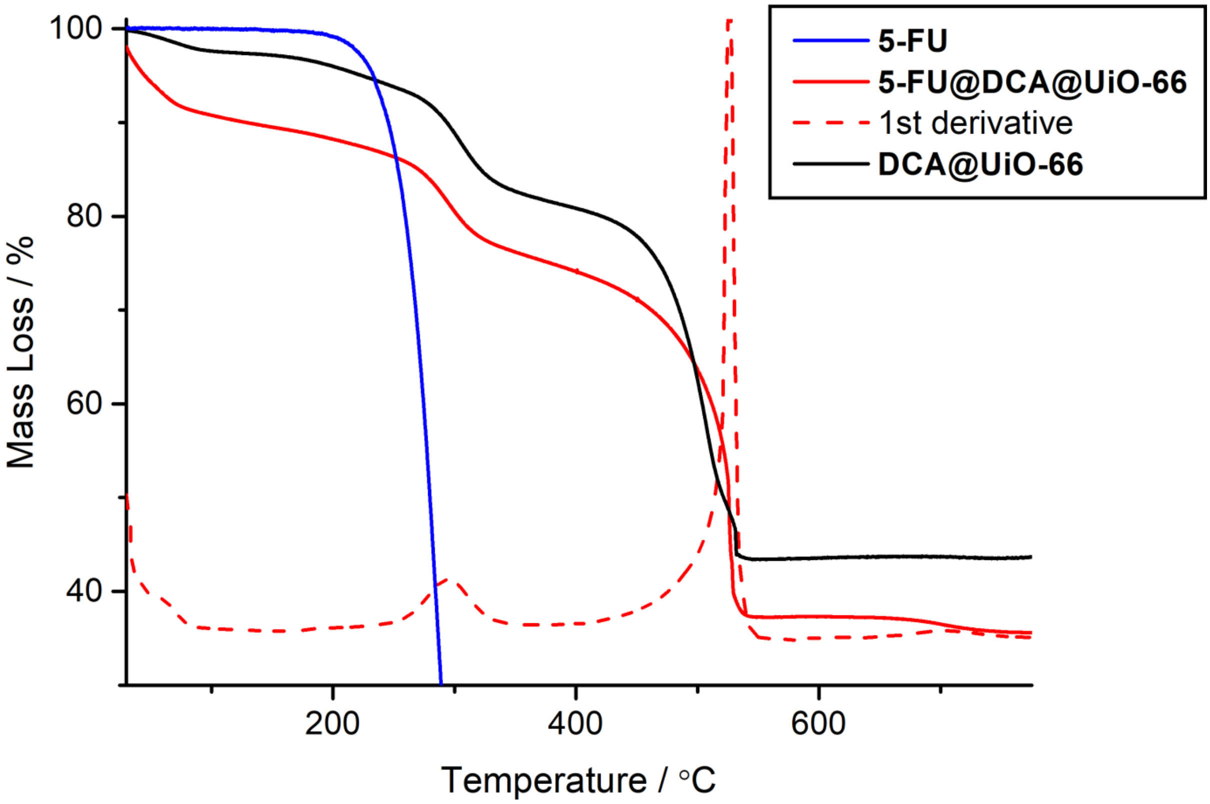

Figure S11. TGA of **5-FU@DCA@UiO-66** compared with that of the **DCA@UiO-66** precursor and free 5-FU. The 5-FU-loaded sample has a similar profile to the precursor. After initial solvent loss, the 5-FU mass loss events overlap with DCA mass loss events corresponding to a total of ca. 11% *w/w* of cargo, in good agreement with values calculated from ICP-OES analysis for DCA (8% *w/w*) and 5-FU (1.2% *w/w*).

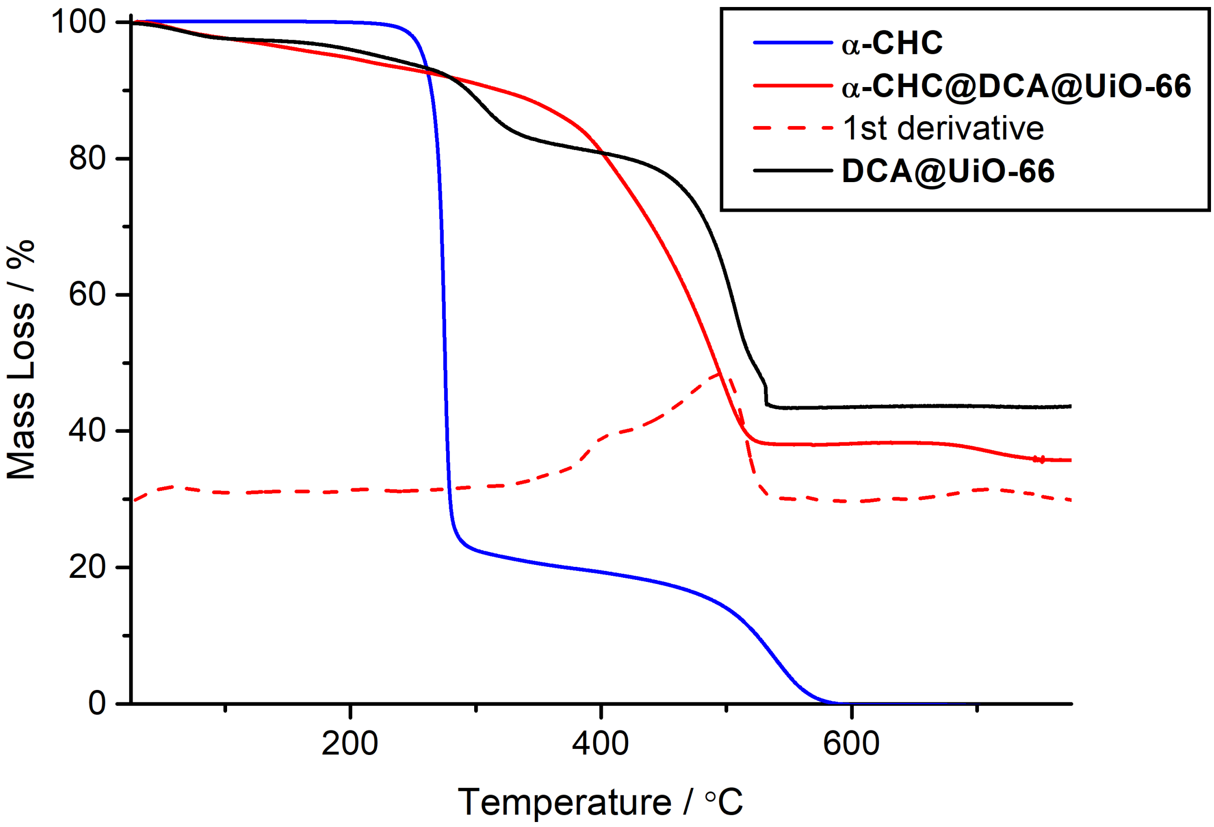

Figure S12. TGA of **α-CHC@DCA@UiO-66** compared to the precursor **DCA@UiO-66** and free α-CHC. The mass loss ascribed to DCA is lost for **α-CHC@DCA@UiO-66**, showing DCA has been mostly replaced by α-CHC, degradation of which occurs at a higher temperature than free α-CHC as a consequence of its attachment to the Zr_6 clusters.

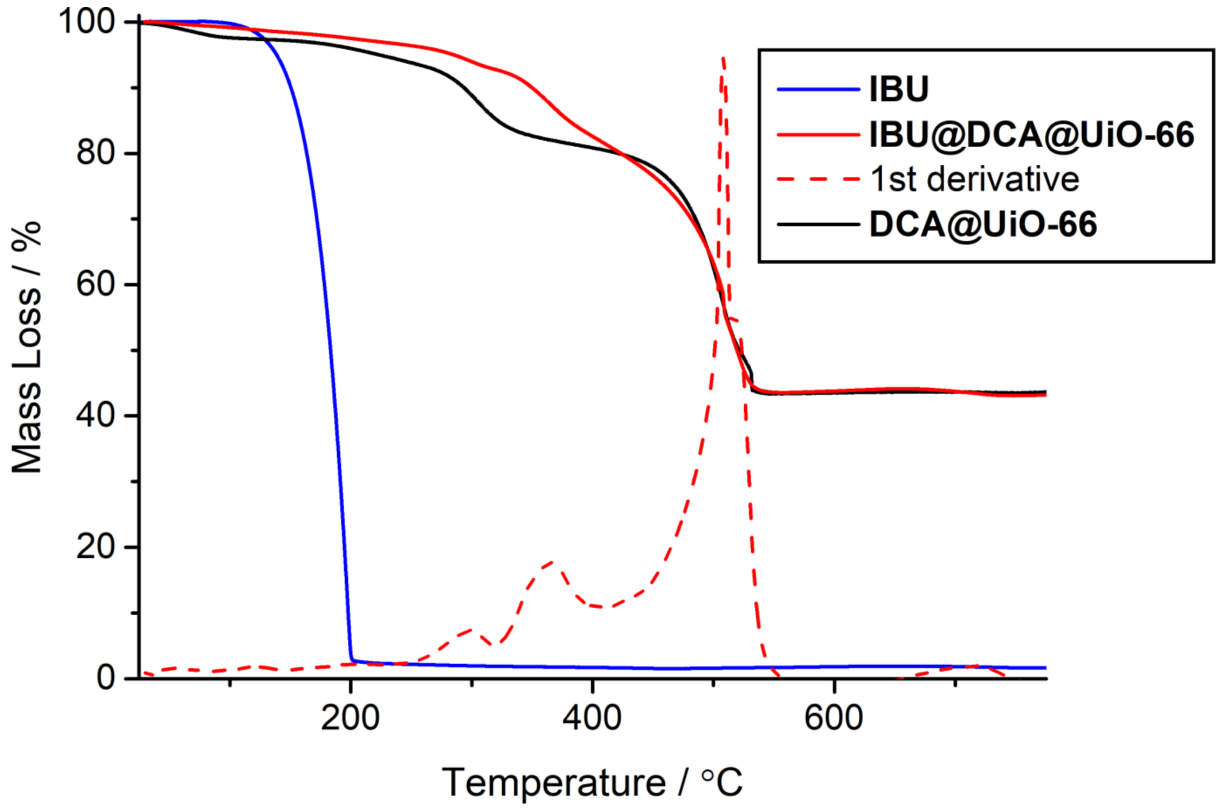

Figure S13. TGA of **IBU@DCA@UiO-66** compared to the precursor **DCA@UiO-66** and free IBU, showing IBU degradation to occur at a higher temperature than free IBU as a consequence of its attachment to the Zr_6 clusters, and partial displacement of DCA from the structure.

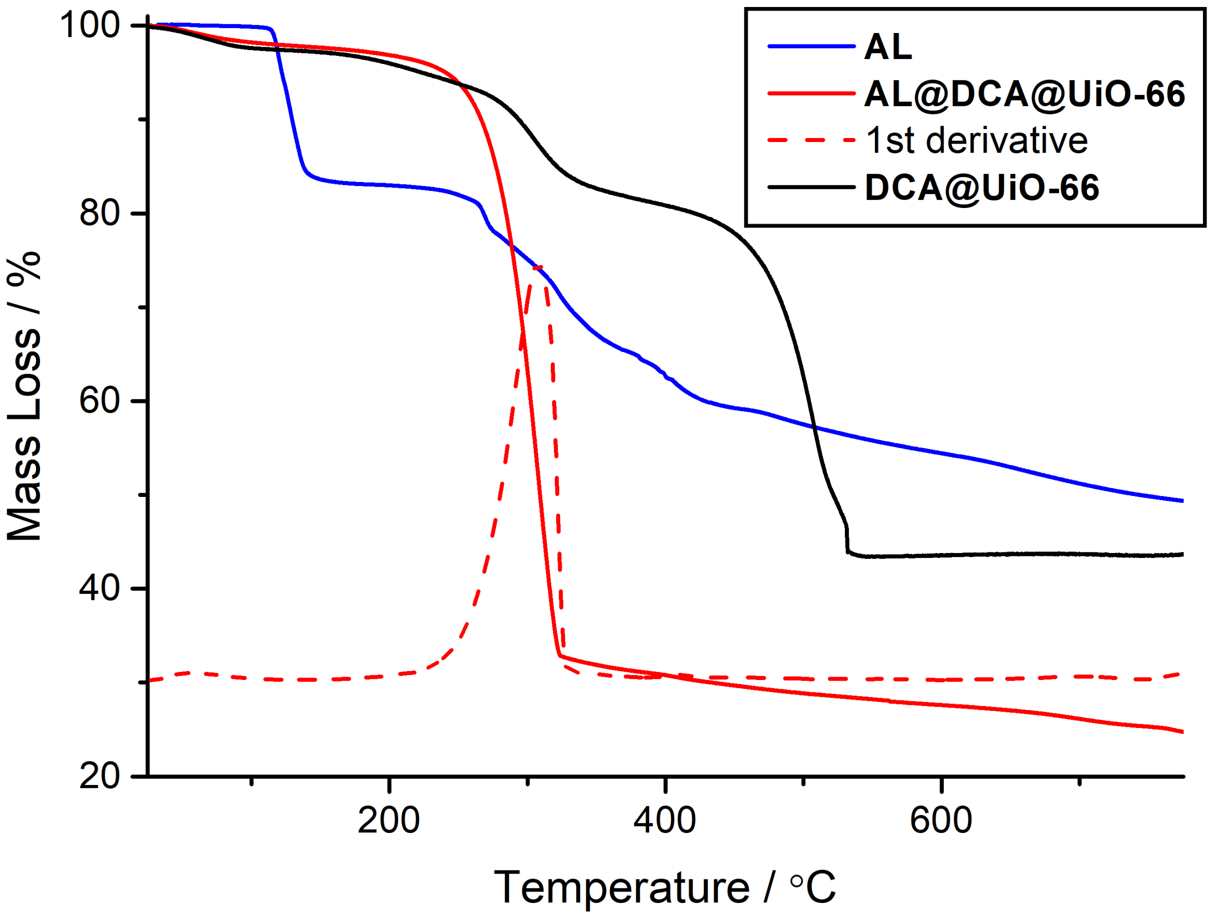

Figure S14. TGA of **AL@DCA@UiO-66** compared to the precursor **DCA@UiO-66** and free AL. showing AL degradation to occur at a higher temperature than free AL as a consequence of its attachment to the Zr_6 clusters, with degradation of the overall structure at a lower temperature, possibly as a consequence of formation of possibly a new structure as indicated by PXRD.

S3.2. Characterisation of UiO-66 Modulated with Single Drugs

The defect-loading of drugs into UiO-66 using the coordination modulation protocol was assessed by PXRD, to examine structure, while thermogravimetric analysis (TGA) and ^{1}H NMR spectra of acid digested samples were used to assess drug uptake and scanning electron microscopy (SEM) to visualise particle size.

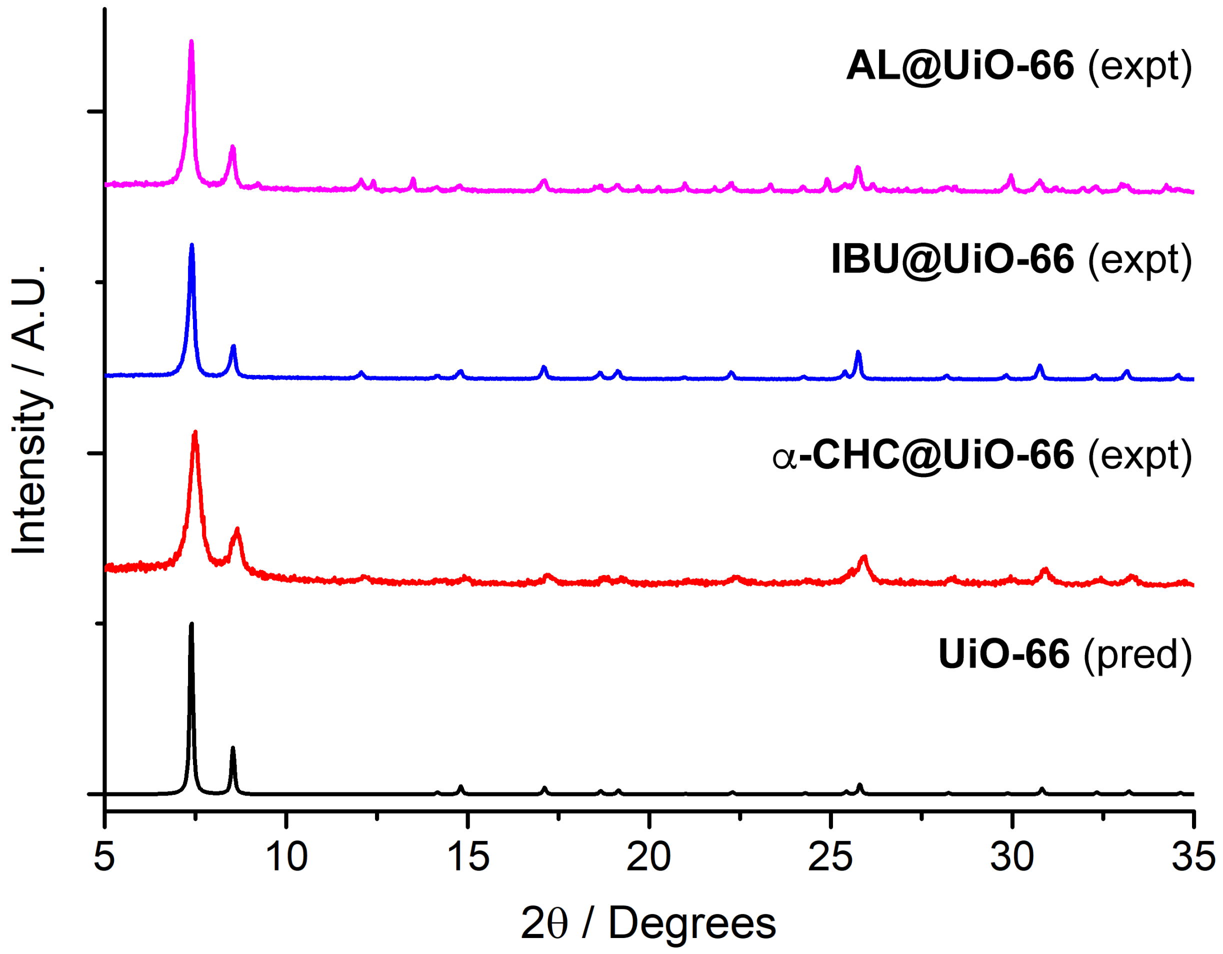

Figure S15. PXRD or the UiO-66 samples modulated with one single drug, showing remarkable high crystallinity in the case of IBU@UiO-66 and suggesting a smaller particle size in the case of α-CHC@UiO-66, both phase pure with the UiO-66 topology, whilst AL@UiO-66 shows the presence of a new phase or of a different structure.

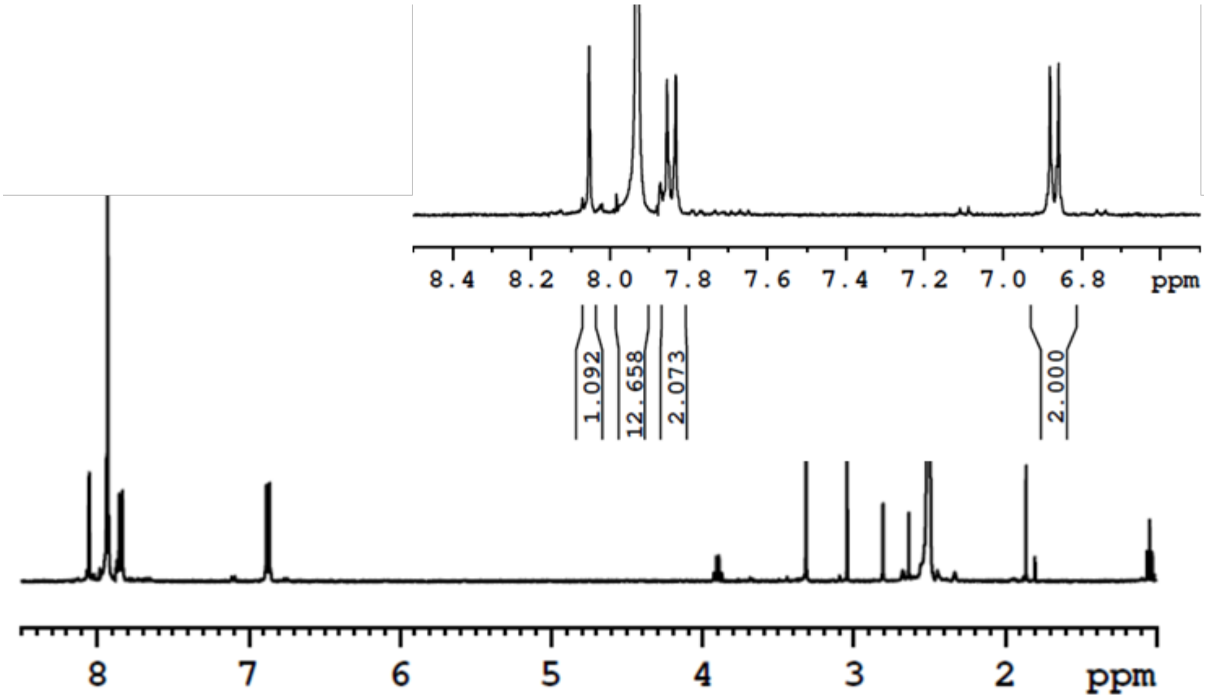

Figure S16. ^{1}H NMR spectrum of digested **α-CHC@UiO-66** in acidified DMSO-d_6, showing 24 mol% loading of α-CHC compared to BDC (ca.1 α-CHC per 4 BDC).

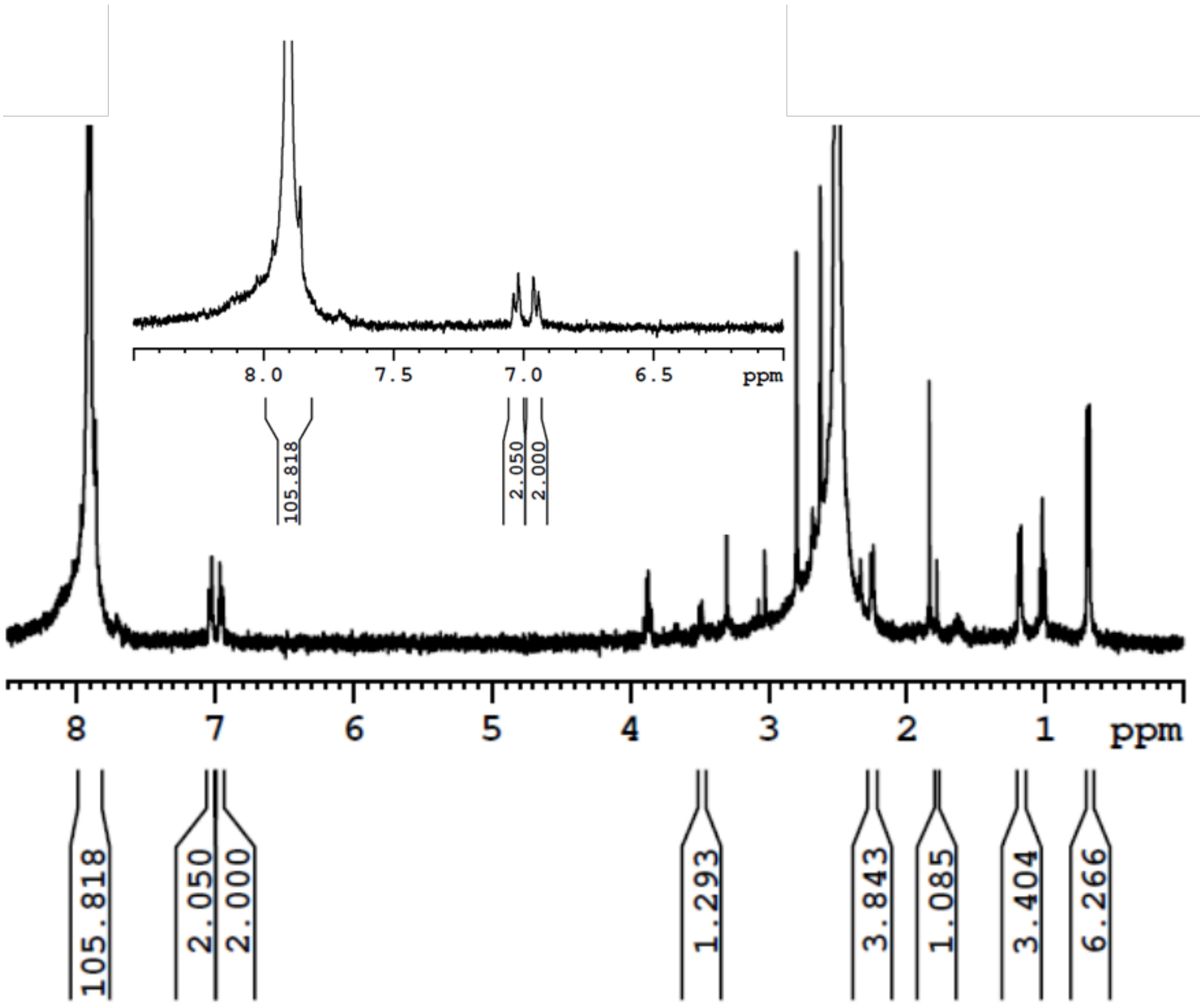

Figure S17. ^{1}H NMR spectrum of acid digested **IBU@UiO-66** in acidified DMSO-d_6, showing 3.6 mol% incorporation of IBU compared to BDC. This low incorporation might be a consequence of the higher pK_a of IBU (4.9), resulting in a low degree of deprotonation during synthesis and hence a low competition with the linker for the coordination to the Zr positions.

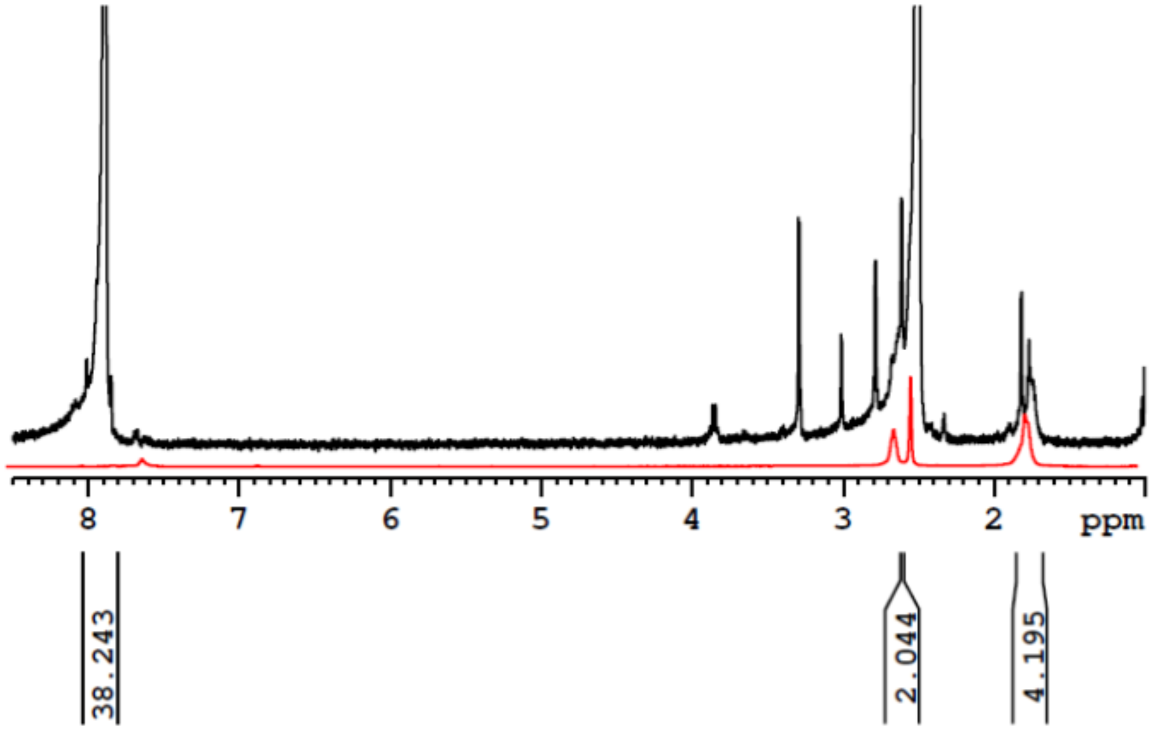

Figure S18. ^{1}H NMR spectrum of digested **AL@UiO-66** in acidified DMSO-d_6, showing 9.9 mol% incorporation of AL compared to BDC (ca. 1 alendronate per 10 BDC).

Table S2. Summary of relative molar drug loading values in single drug modulated MOFs correlated with the pK_a of the drug.

MOF	Drug Loading / mol % compared to BDC (^{1}H NMR)			1st pK_a of drug
	AL	α-CHC	IBU	
**AL@UiO-66**	9.9%	–	–	2.4
**α-CHC@UiO-66**	–	24.0%	–	2.2
**IBU@UiO-66**	–	–	3.6%	4.9

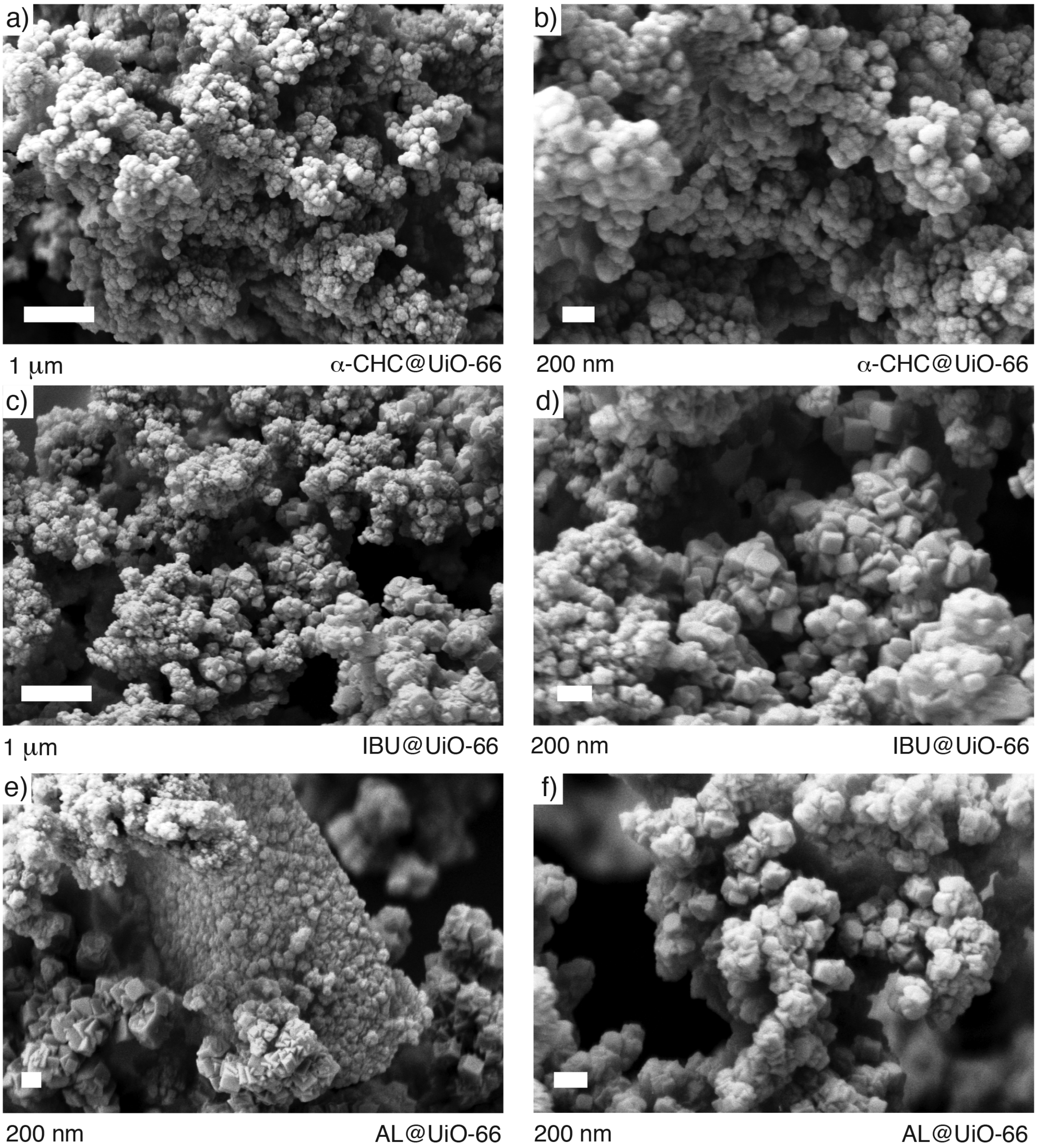

Figure S19. SEM images of the MOFs modulated with one single drug showing their aggregation and agglomeration, and therefore unsuitability for drug delivery applications. a) and b) **α-CHC@UiO-66**, c) and d) **IBU@UiO-66**, e) and f) **AL@UiO-66**.

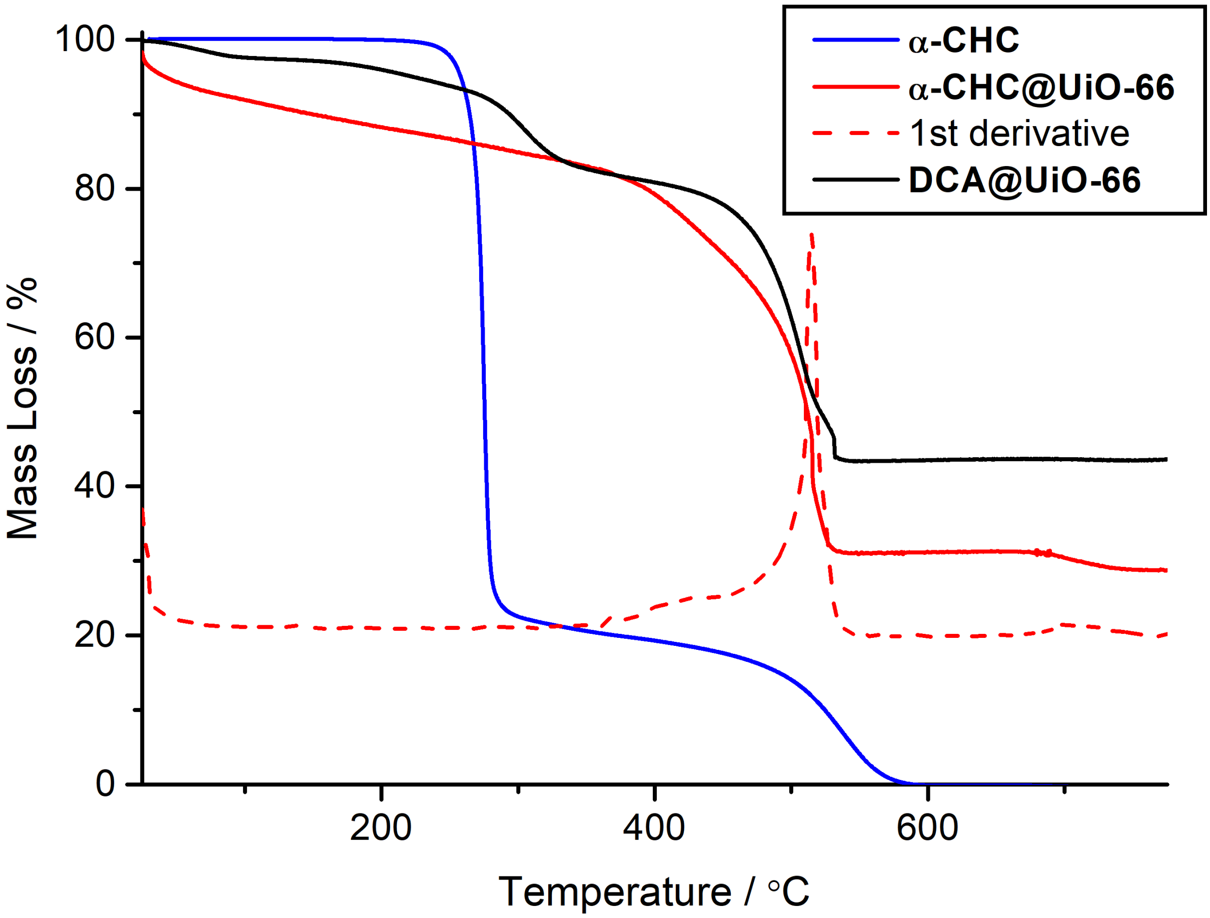

Figure S20. TGA profiles of free α-CHC, **α-CHC@UiO-66** and **DCA@UiO-66**, showing the higher temperature thermal decomposition of α-CHC when coordinated within **α-CHC@UiO-66**, and the earlier overall decomposition of its structure compared to **DCA@UiO-66**, all consequence of α-CHC attachment to the Zr clusters. The mass loss event as α-CHC thermally decomposes overlaps with overall decomposition, making it difficult to assess loading by TGA.

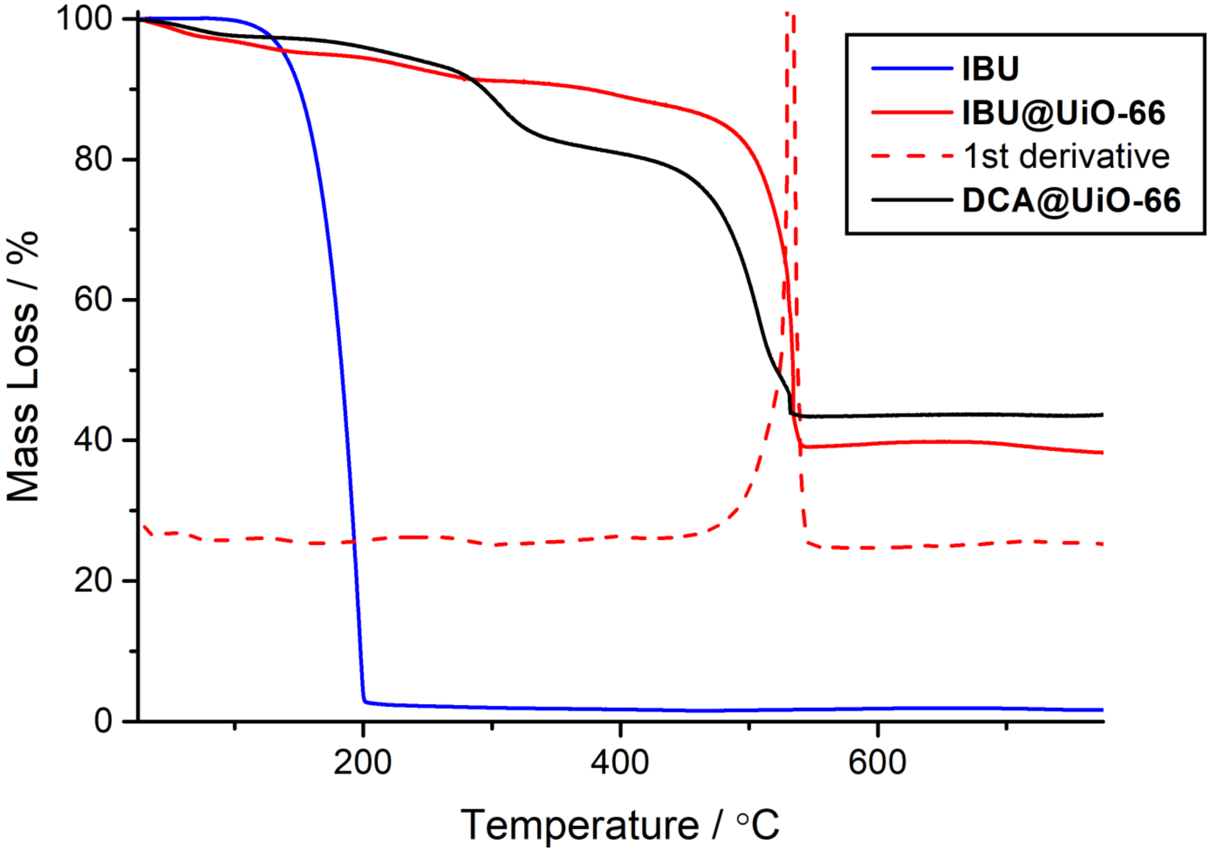

Figure S21. TGA profiles of free IBU, **IBU@UiO-66** and **DCA@UiO-66**, showing the higher temperature thermal decomposition of IBU (ca. 2% *w/w* around 350-400 ºC) within **IBU@UiO-66**, suggestive of attachment at Zr_6 clusters.

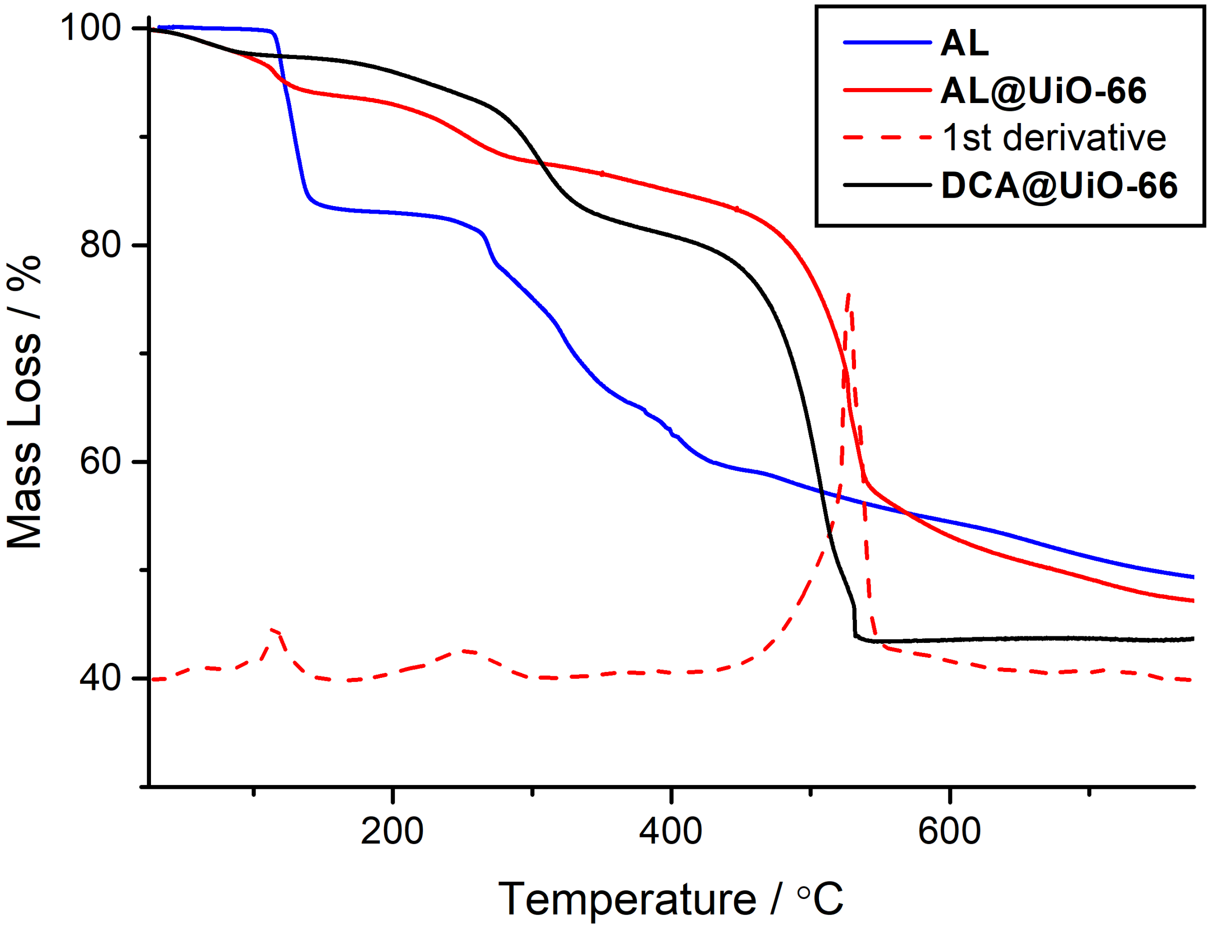

Figure S22. TGA profile of free AL, **AL@UiO-66** and **DCA@UiO-66**, showing the higher temperature thermal decomposition of AL. Due to the gradual thermal decomposition of AL within UiO-66, its determination by TGA is not possible, although the similar decomposition of free AL to AL within the structure suggests that some component could be absorbed in the surface.

S3.3. Characterisation of UiO-66 Modulated with Two Drugs

The defect-loading of two drugs into UiO-66 using the coordination modulation protocol (multivariate modulation) was assessed by PXRD (see Figure 2a in the manuscript) to examine structure, while thermogravimetric analysis (TGA) and ^{1}H NMR spectra of acid digested samples were used to assess drug uptake and scanning electron microscopy (SEM) to visualise particle size. N_2 adsorption/desorption isotherms were used to assess porosity and the location of the modulators.

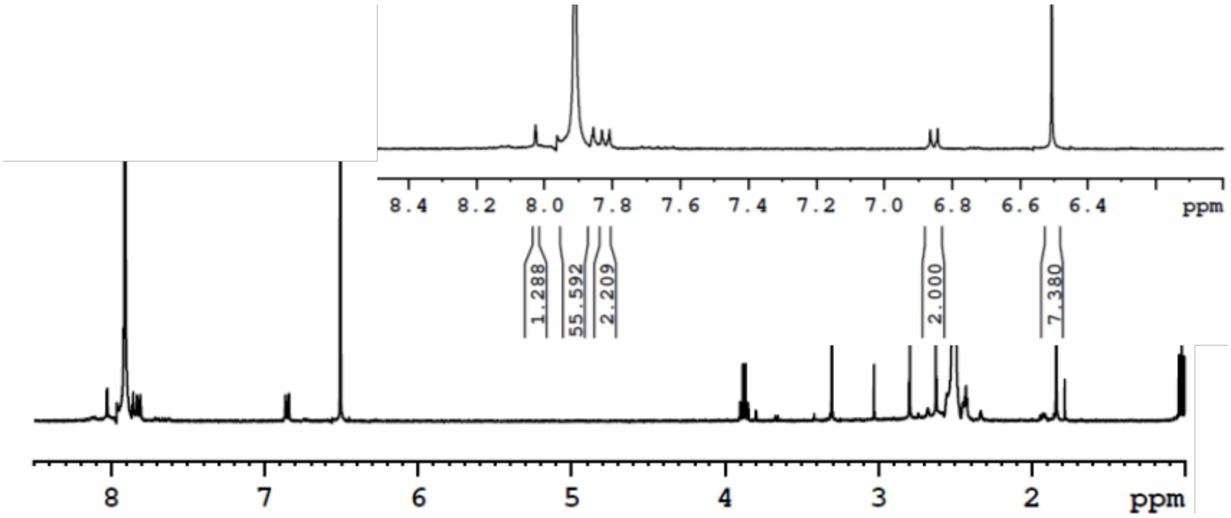

Figure S23. ^{1}H NMR spectrum of digested **α-CH/DCA@UiO-66** in acidified DMSO-d_6, showing α-CHC incorporation of 6.7 mol% compared to BDC, which is reduced compared to the single α-CHC modulated sample as a consequence of DCA incorporation at 35 mol% compared to BDC (ca. 1 DCA per 3 BDC).

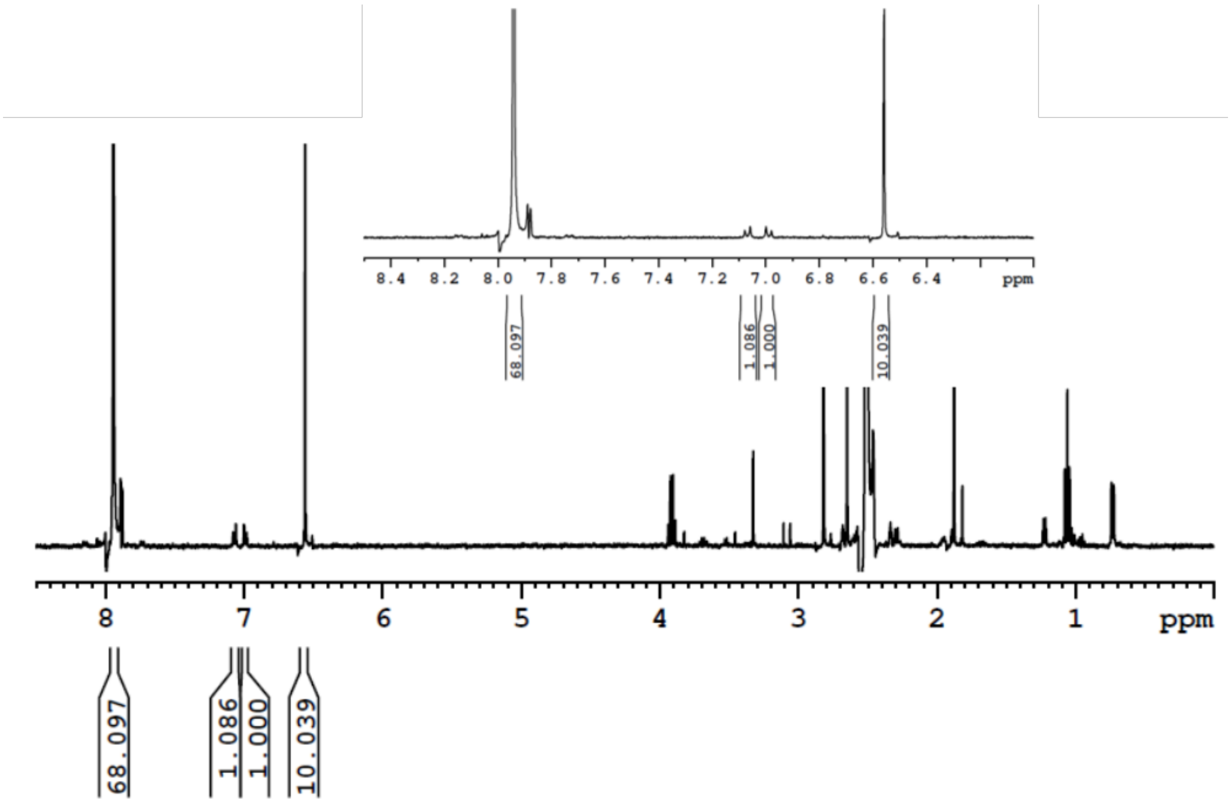

Figure S24. ^{1}H NMR spectrum of **IBU/DCA@UiO-66** in acidified DMSO-d_6, showing an IBU content (2.9 mol%) lower that the single IBU modulated sample, a consequence of the competition with DCA, which has an incorporation of 37.1 mol% (ca. 1 DCA per 2.5 BDC).

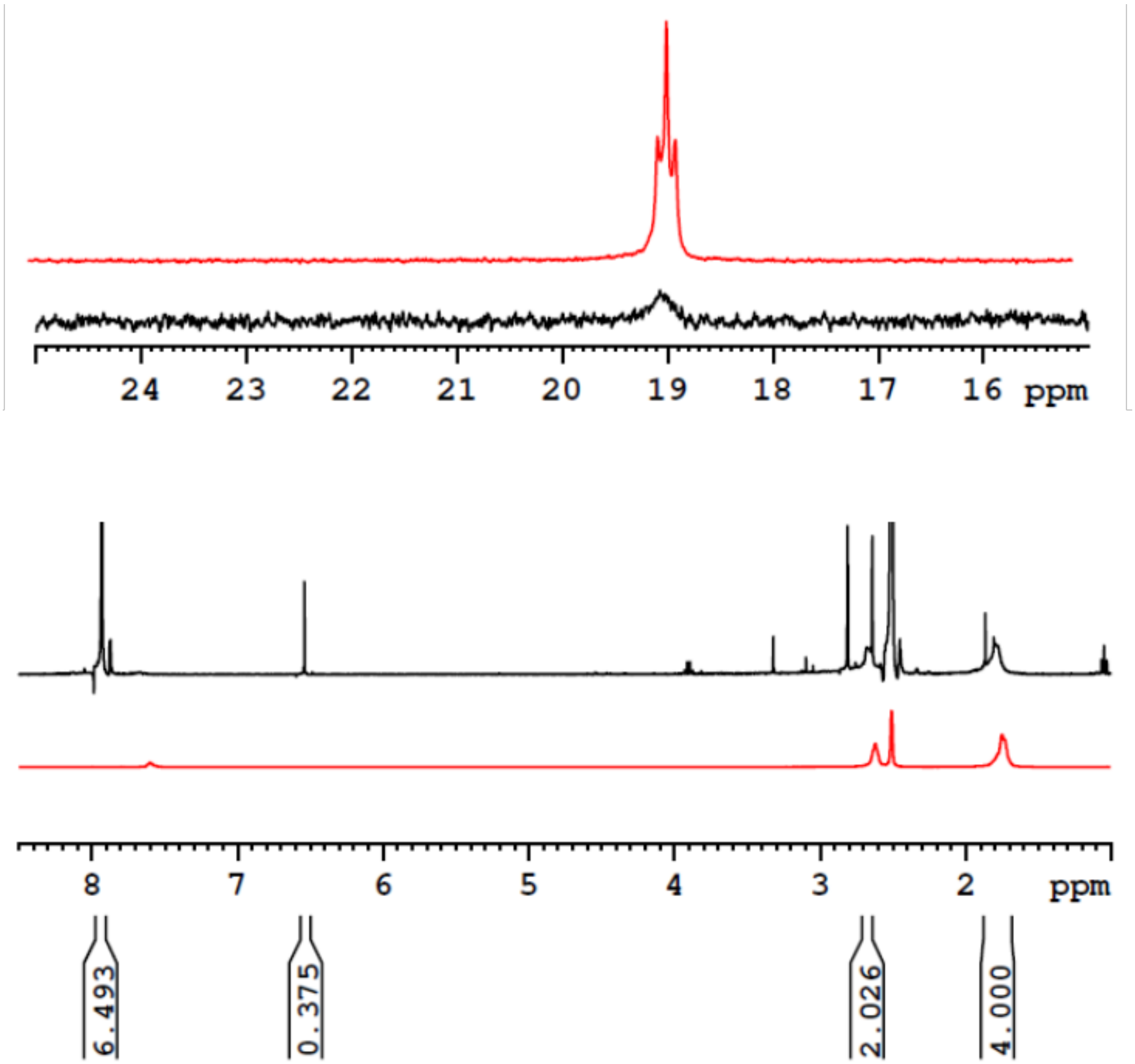

Figure S25. NMR spectra in acidified DMSO-d_6 of **AL/DCA@UiO-66** (black) and free AL (red) – ^{31}P (top) and ^{1}H (bottom) – showing a remarkably high incorporation of AL (38.1 mol% compared to BDC, 1 AL per 2.6 BDC) and a DCA incorporation of 18.8 mol% (ca. 1 DCA per 5 BDC).

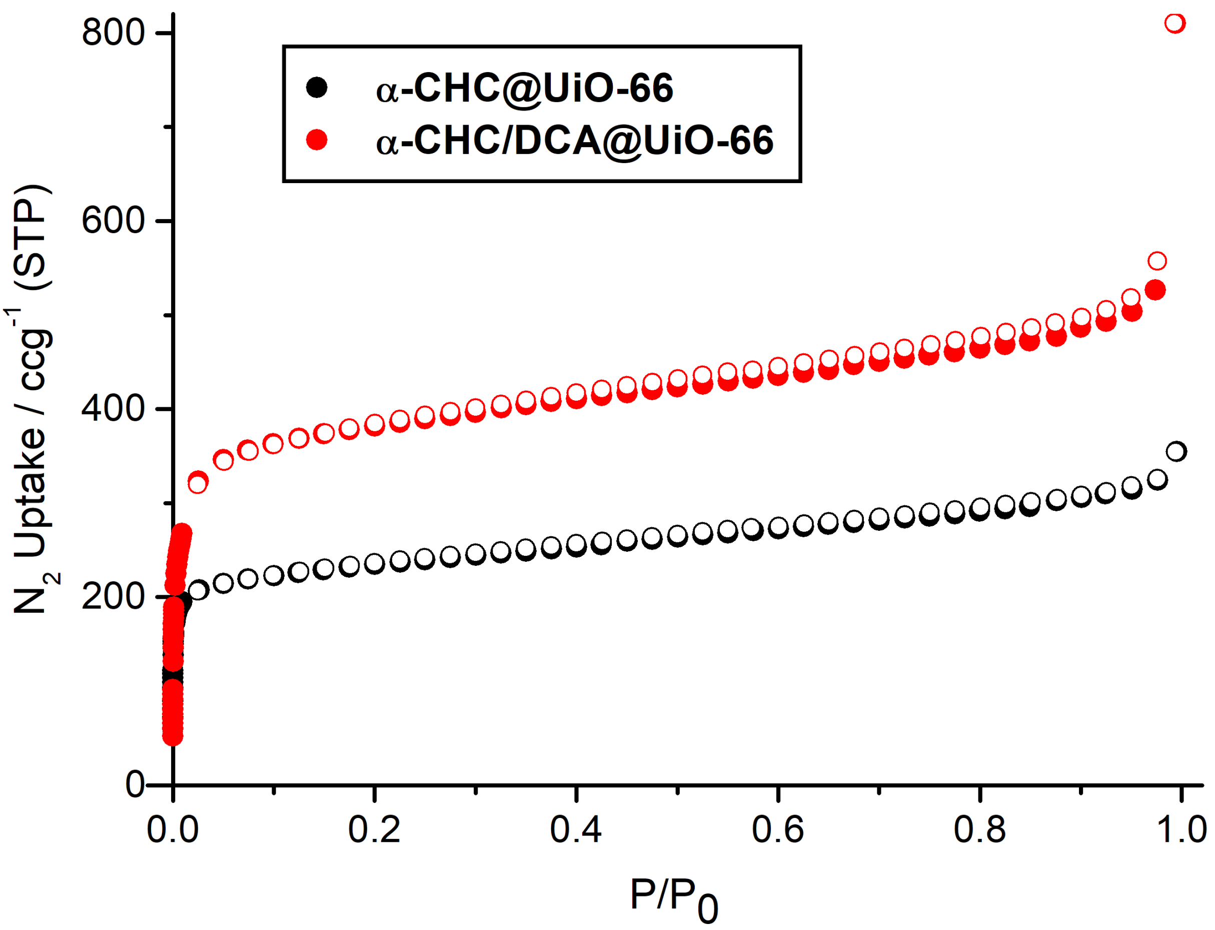

Figure S26. N_2 adsorption (filled symbols) and desorption (empty symbols) measurements at 77 K of **α-CHC@UiO-66** and **α-CHC/DCA@UiO-66**, showing the increase in porosity upon DCA addition:

α-CHC@UiO-66: S_{BET} = 989 m^2g^{-1}; pore volume= 0.46 ccg^{-1}.

α-CHC/DCA@UiO-66: S_{BET} = 1485 m^2g^{-1}; pore volume= 0.77 ccg^{-1}.

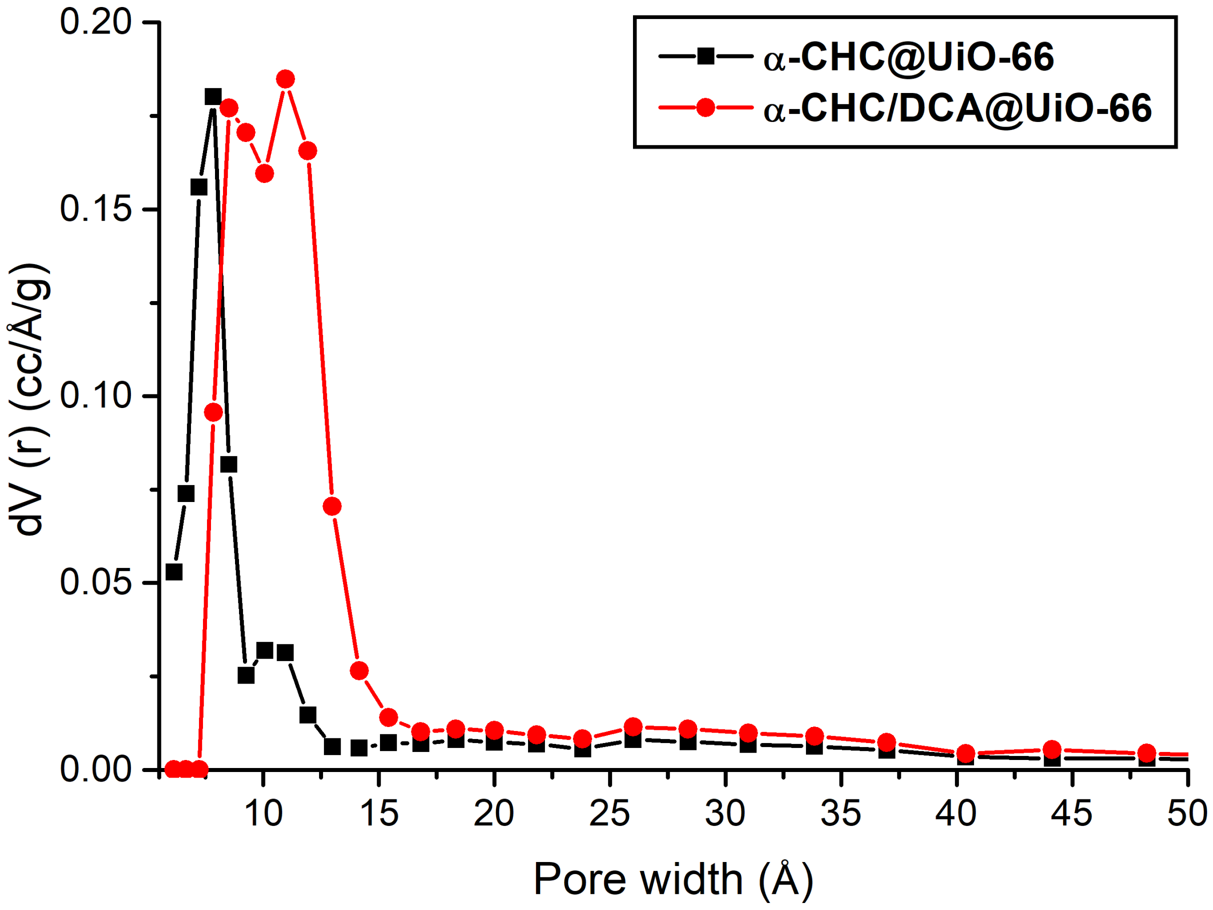

Figure S27. Pore size distribution of **α-CHC@UiO-66** and **α-CHC/DCA@UiO-66** showing well-defined pores of ca. 8 and 11 Å for **α-CHC@UiO-66**, in agreement with the characteristic pores of UiO-66,[S2] whilst **α-CHC/DCA@UiO-66** has bigger pores as a consequence of its significant DCA-induced defectivity.

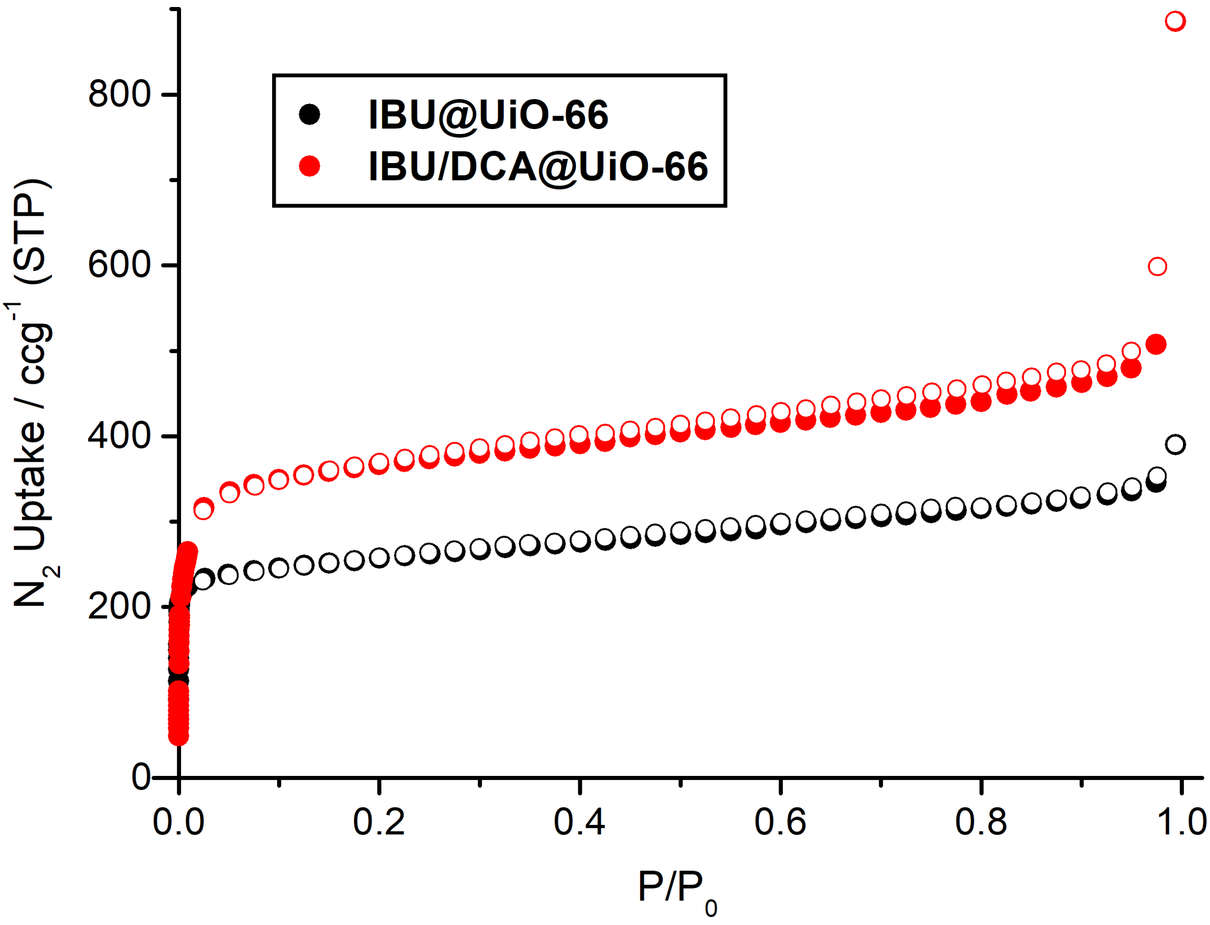

Figure S28. N_2 adsorption (filled symbols) and desorption (empty symbols) measurements at 77 K of **IBU@UiO-66** and **IBU/DCA@UiO-66**, showing the increase in surface area upon DCA addition:

IBU@UiO-66: S_{BET} = 1111 m^2g^{-1}; pore volume = 0.60 ccg^{-1}.

IBU/DCA@UiO-66: S_{BET} = 1440 m^2g^{-1}; pore volume =0.78 ccg^{-1}.

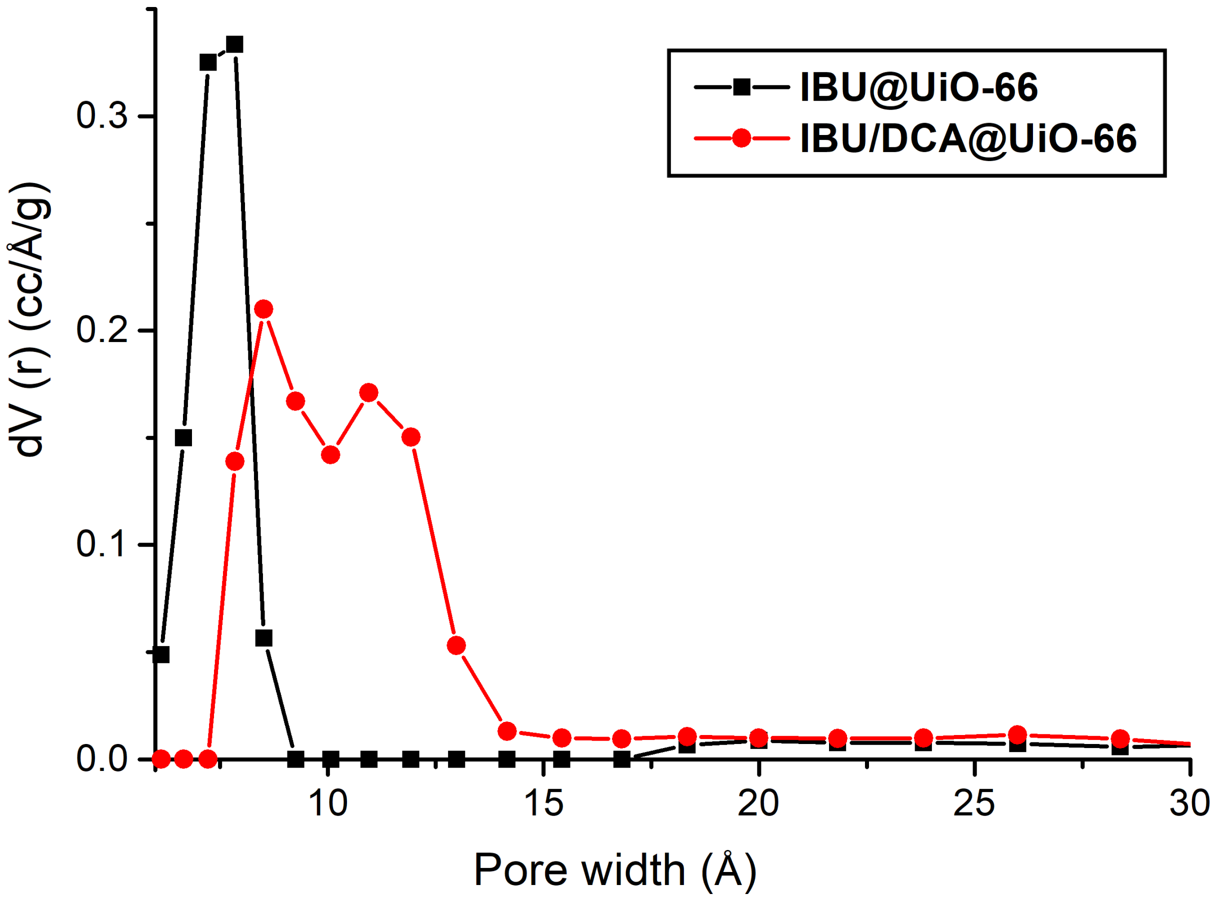

Figure S29. Pore size distribution of **IBU@UiO-66** and **IBU/DCA@UiO-66** showing that the latter has larger pores consequence of its DCA-induced defectivity.

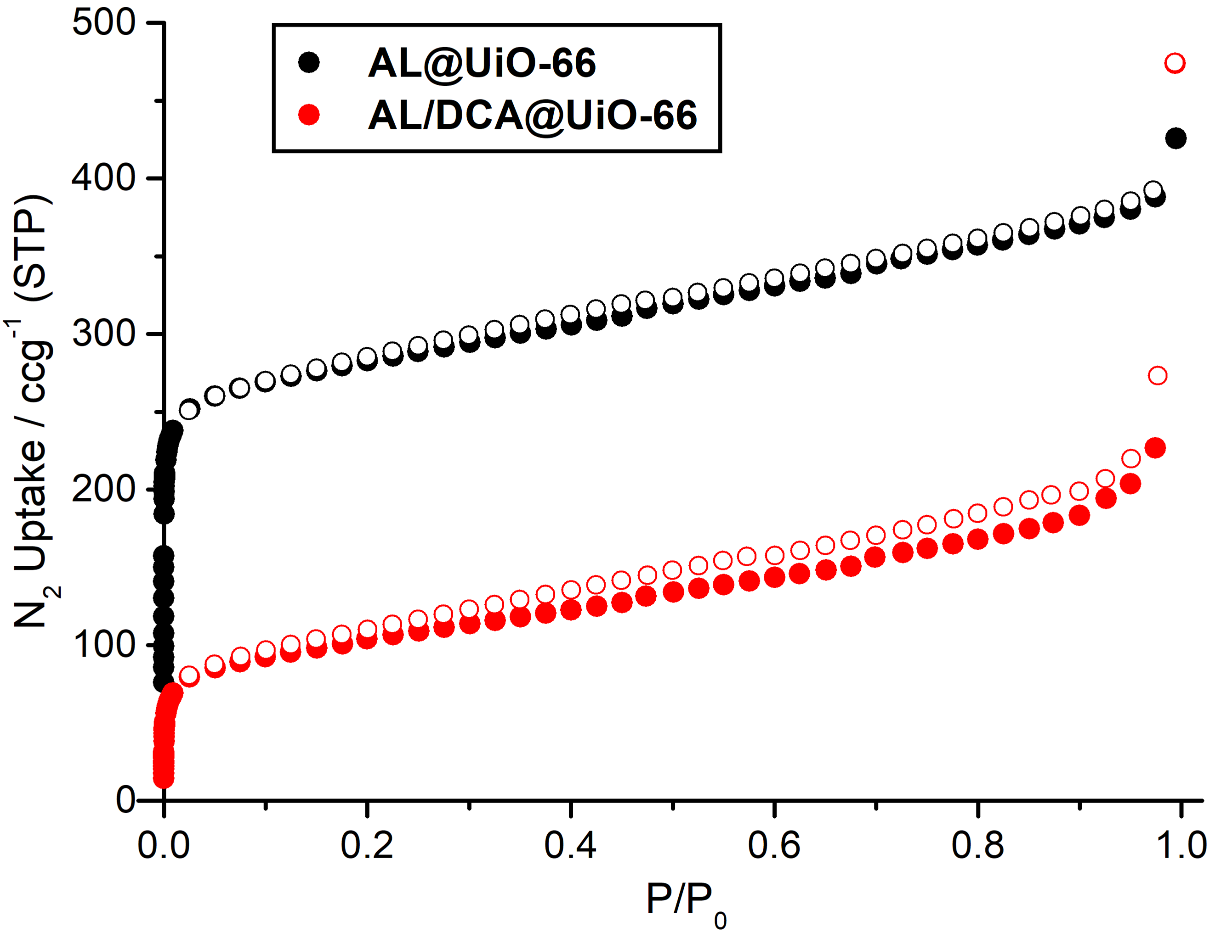

Figure S30. N_2 adsorption (filled symbols) and desorption (empty symbols) measurements at 77 K of **AL@UiO-66** (CM) and **AL/DCA@UiO-66** (CM), showing a significant decrease in surface area upon DCA addition, possibly due to increased uptake of AL and poorer crystallinity of the sample:

Al@UiO-66: S_{BET} = 1245 m^2g^{-1}; pore volume = 0.56 ccg^{-1}.
Al/DCA@UiO-66: S_{BET} = 369 m^2g^{-1}; pore volume = 0.35 ccg^{-1}.

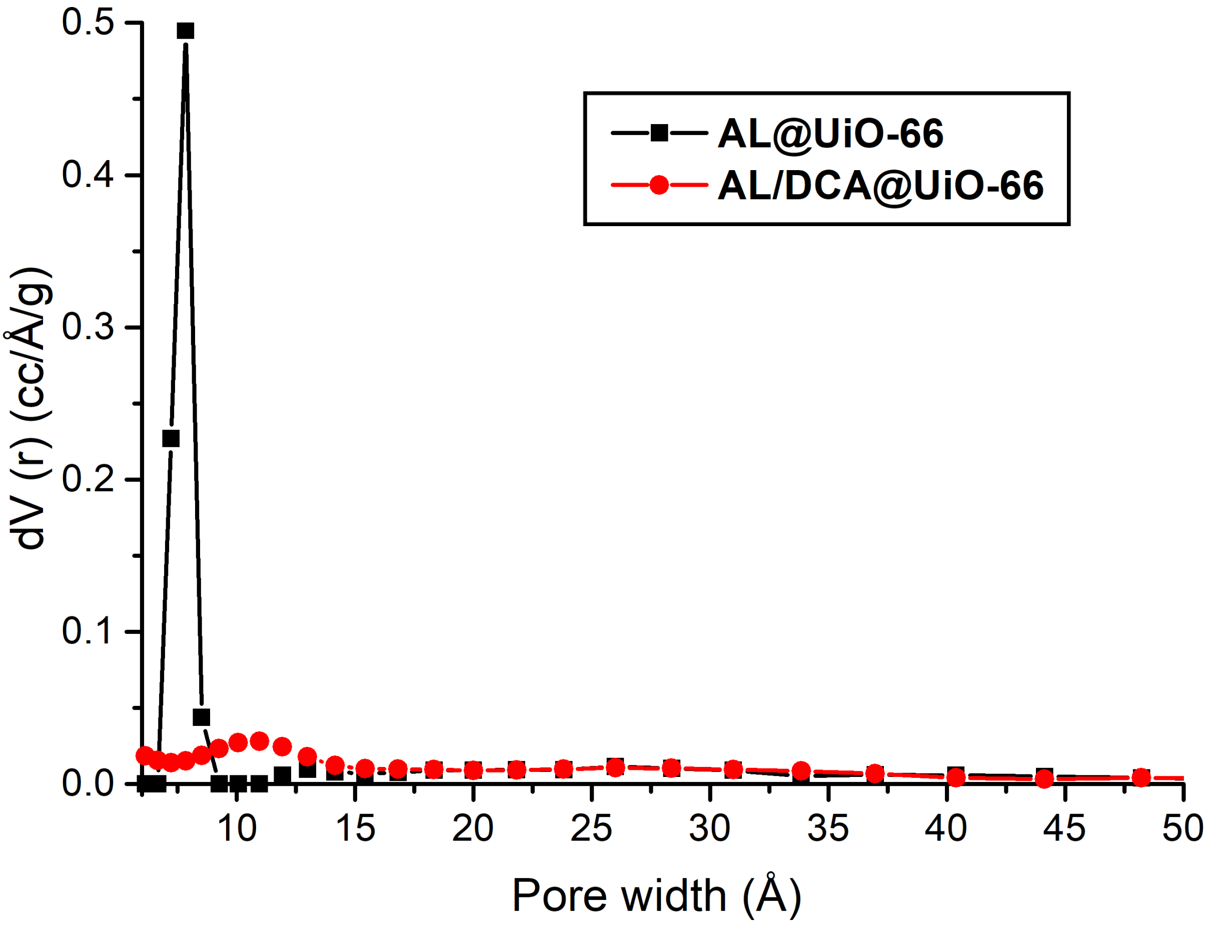

Figure S31. Pore size distribution of **AL@UiO-66** and **AL/DCA@UiO-66** showing well-defined pores of ca. 8 and 11 Å for **AL@UiO-66**, in good agreement with the characteristic pores of UiO-66,[S2] whilst **AL/DCA@UiO-66** has a poorly defined pore structure, correlating with the low crystallinity of the sample observed by PXRD.

Table S3. Comparison of relative molecular drug loadings and surface areas for the single- and dual-drug modulated MOF samples. Drug loading values by mass for the samples used in drug delivery experiments are given in Table 1 of the main manuscript.

MOF	Drug Loading / mol % compared to BDC (^{1}H NMR)				BET Surface Area / m^2g^{-1}
	DCA	AL	α-CHC	IBU	
**DCA@UiO-66**	36.0%	–	–	–	1488[a]
**AL@UiO-66**	–	9.9%	–	–	1245
**AL/DCA@UiO-66**	18.8%	38.1%	–	–	369
**α-CHC@UiO-66**	–	–	24.0%	–	989
**α-CHC/DCA@UiO-66**	35.0%	–	6.6%	–	1485
**IBU@UiO-66**	–	–	–	3.6%	1111
**IBU/DCA@UiO-66**	37.1%	–	–	2.9%	1440

[a]Value taken from reference [S1]. Values for different batches are typically ~1500 m^2g^{-1}.

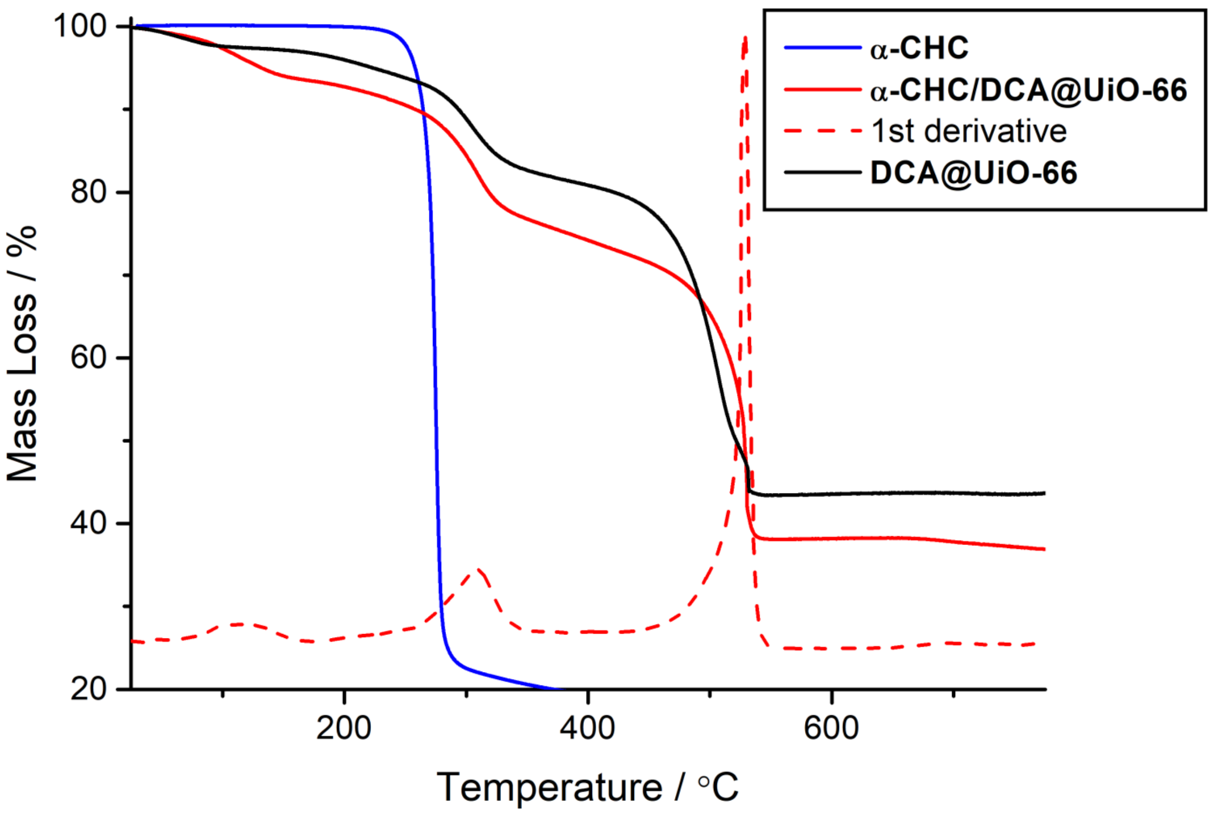

Figure S32. TGA profile of **α-CHC/DCA@UiO-66** compared to α-CHC and **DCA@UiO-66**. Due to the α-CHC and DCA mass losses overlapping, quantification of either component is not possible by this technique. Mass loss events occur at higher temperatures than for either free drug, confirming attachment as defects.

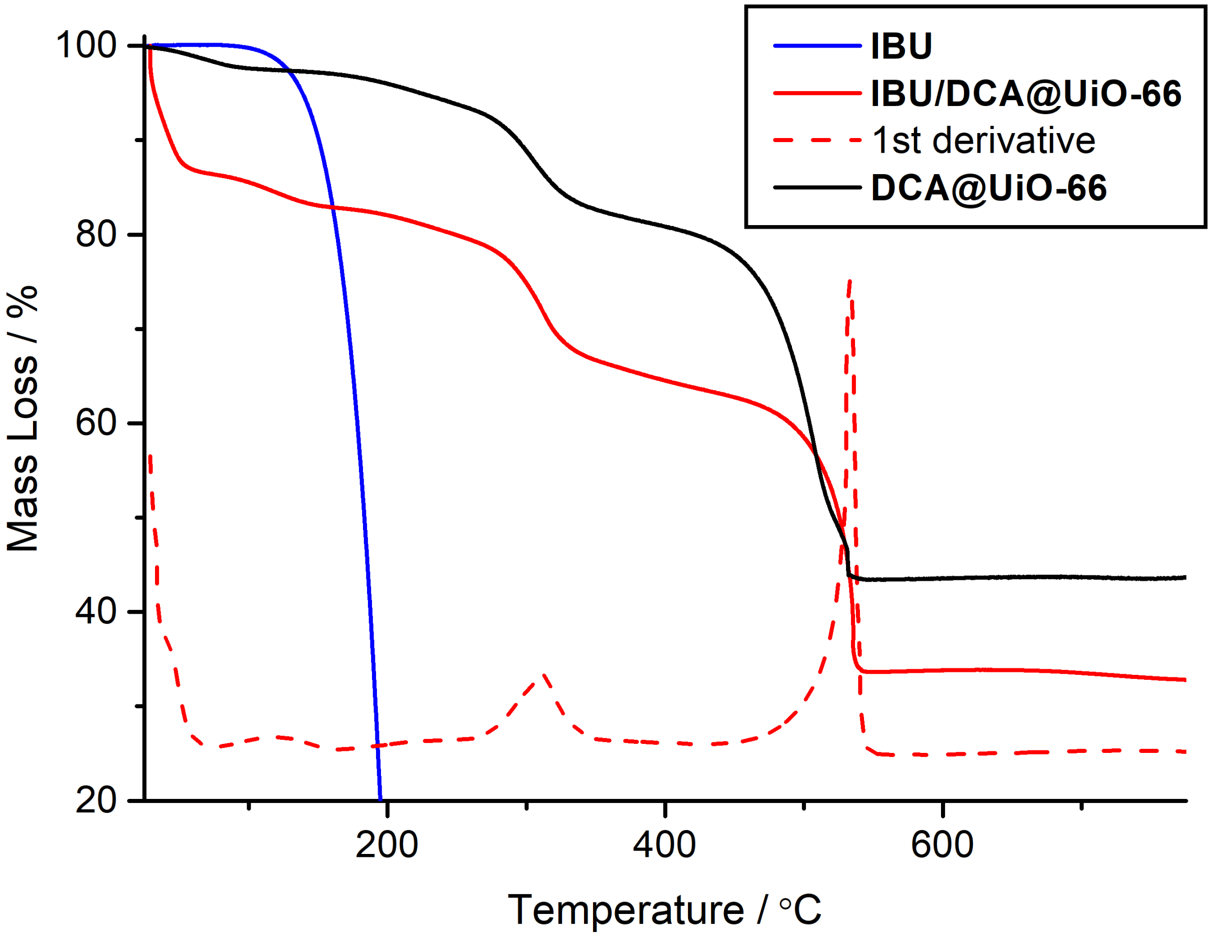

Figure S33. TGA profiles of free IBU, **IBU/DCA@UiO-66** and **DCA@UiO-66**, showing mass loss events occurring at a higher temperature than the free drugs, confirming their attachment.

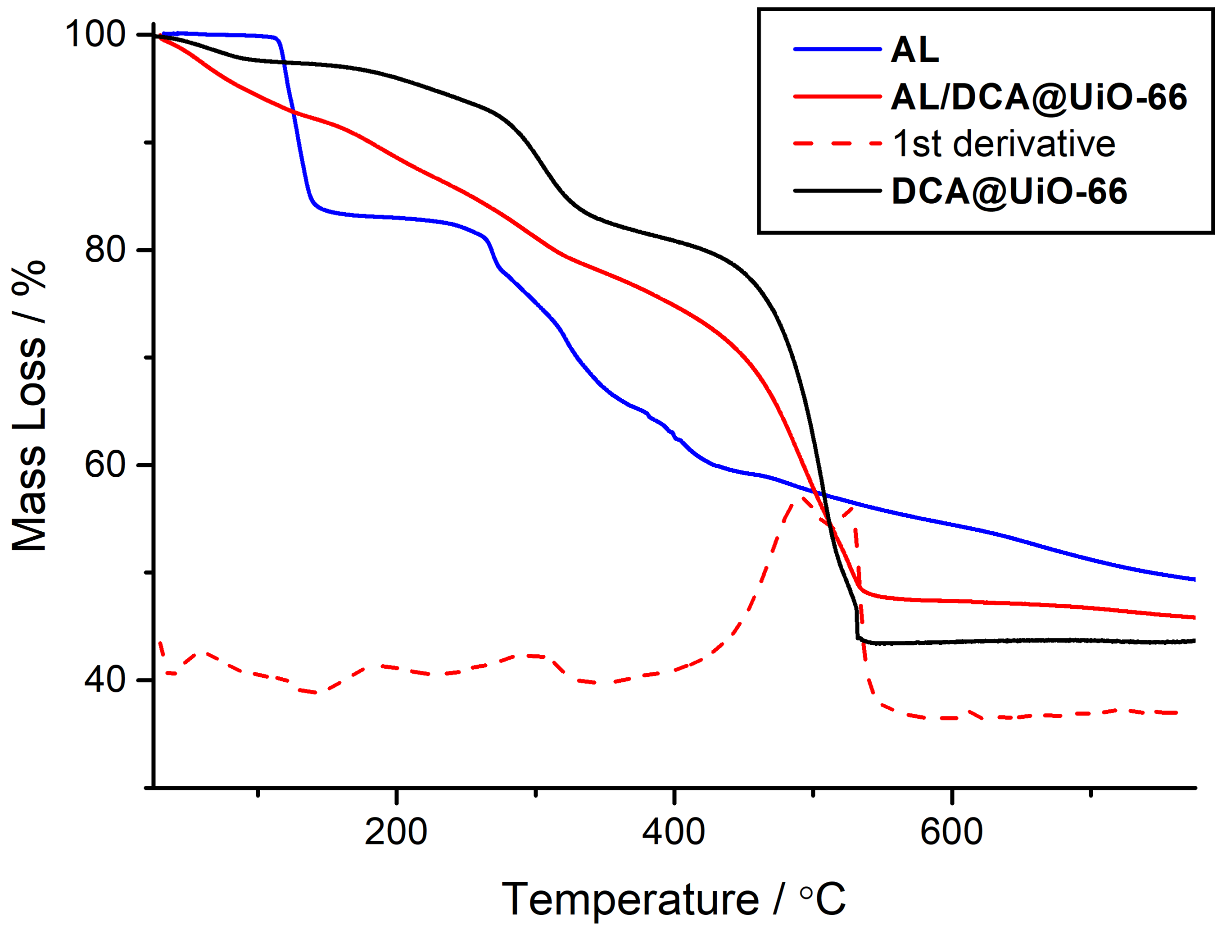

Figure S34. TGA profiles of free AL, **AL/DCA@UiO-66** and **DCA@UiO-66**, showing the more gradual decomposition of the **AL/DCA@UiO-66** structure which is suggestive of possible structural differences compared to UiO-66 and significant incorporation of AL.

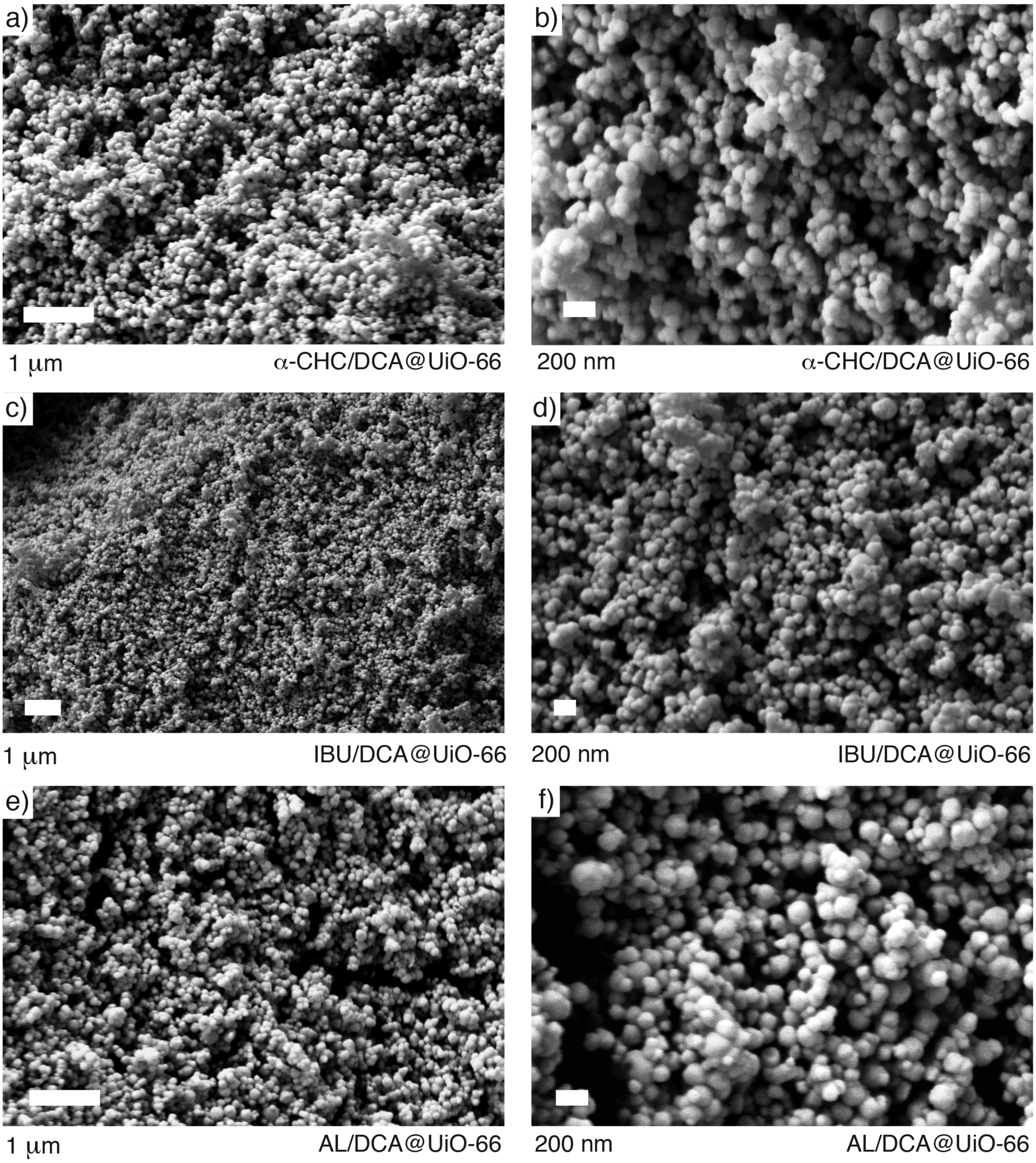

Figure S35. SEM images of the dual drug-loaded MOFs showing monodisperse spherical nanoparticles of ca. 100 nm and indicating the size-control effect of DCA. a) and b) **α-CHC/DCA@UiO-66**, c) and d) **IBU/DCA@UiO-66**, e) and f) **AL/DCA@UiO-66**.

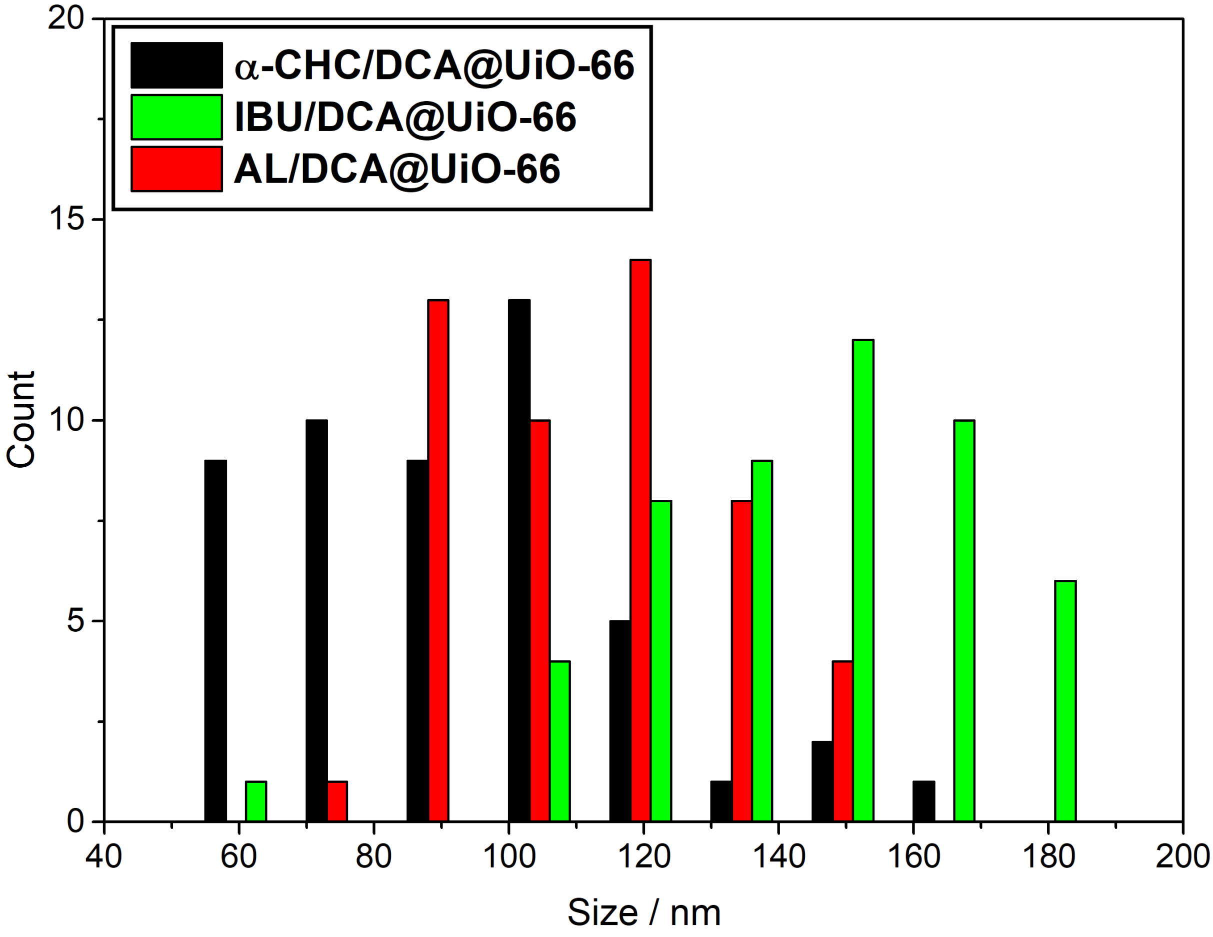

Figure S36. Particle size histogram derived from SEM analysis of dual drug loaded MOFs, calculated from a minimum of 50 particles per MOF.

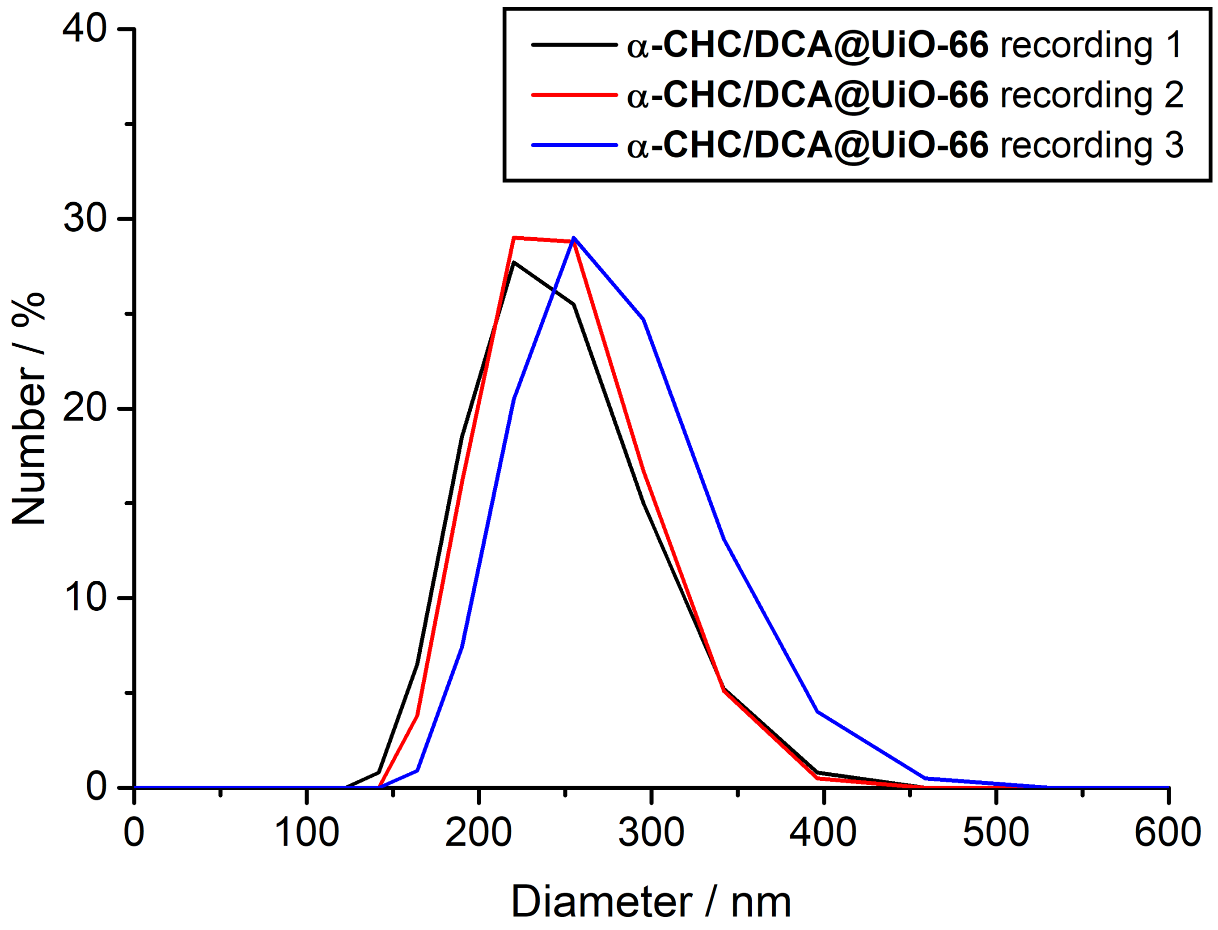

Figure S37. DLS measurements (0.1 mgmL^{-1} in PBS 10X) of **α-CHC/DCA@UiO-66** showing hydrodynamic diameter (ca. 250 nm) close to the one determined by SEM (ca. 100 nm), but indicating minor aggregation.

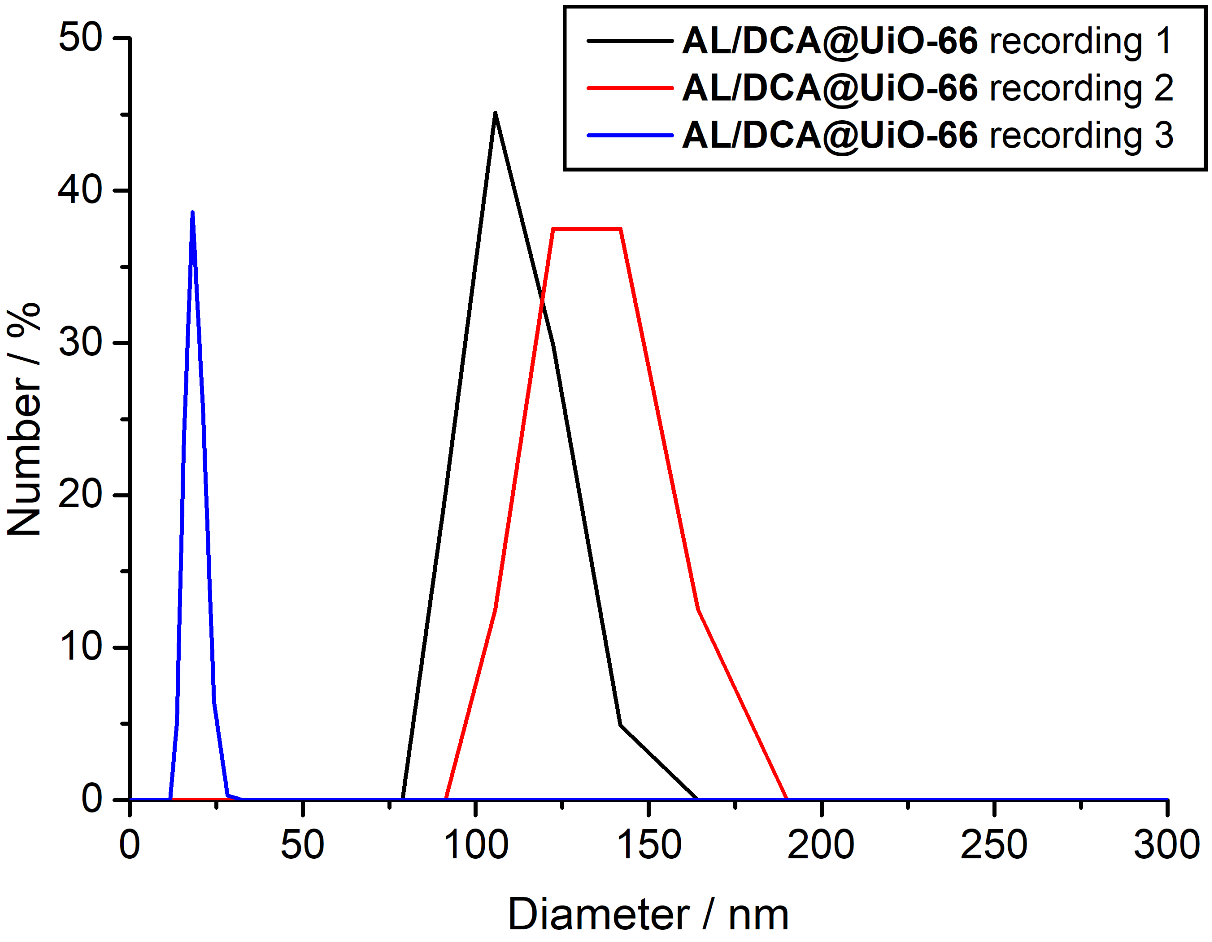

Figure S38. DLS measurements (0.1 mgmL^{-1} in PBS 10X) of **AL/DCA@UiO-66** showing hydrodynamic diameter (ca. 125 nm) very close to the one determined by SEM (ca. 100 nm). The last measurement shows a decrease in hydrodynamic diameter, which could be a consequence of the sedimentation of larger particles or due to structure degradation. DLS measurements for IBU/DCA@UiO-66 were not collected, as the sample was not considered for drug delivery experiments.

S3.4. Characterisation of UiO-66 Modulated with Three Drugs

The defect-loading of three drugs into UiO-66 using the coordination modulation protocol (multivariate modulation) to form **α-CHC/AL/DCA@UiO-66** was assessed by PXRD (see Figure 2a in the manuscript) to examine structure, while thermogravimetric analysis (TGA) and ^{1}H NMR spectra of acid digested samples were used to assess drug uptake and scanning electron microscopy (SEM) to visualise particle size. N_2 adsorption/desorption isotherms were used to assess porosity and the location of the modulators.

.

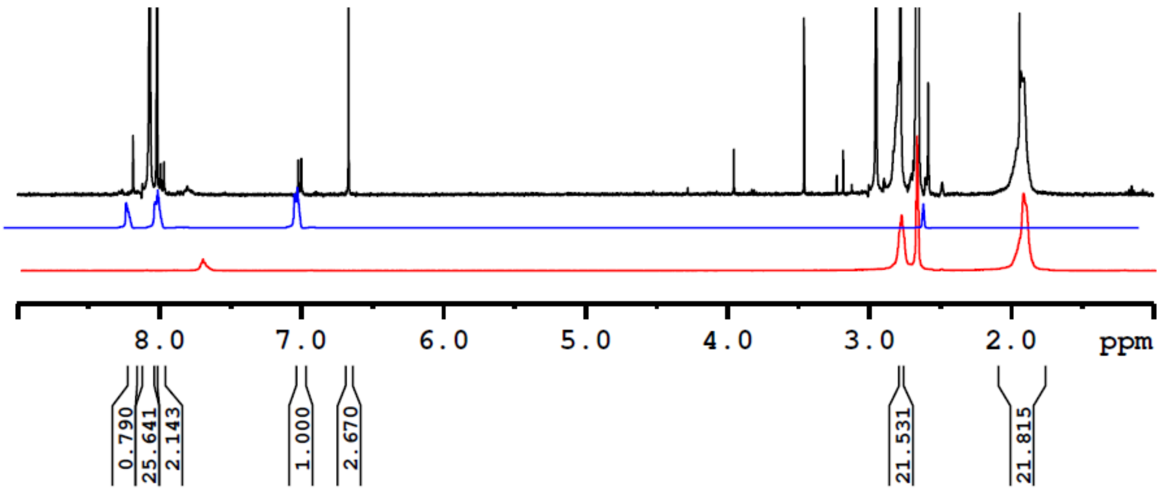

Figure S39. ^{1}H NMR spectrum of **α-CHC/AL/DCA@UiO-66** in acidified DMSO-d_6 (top), compared to α-CHC (middle) and AL (bottom), showing ca. 30 mol% of DCA (ca. 1 DCA per 3 BDC), 7.5 mol% of α-CHC (ca. 1 α-CHC per 12 BDC) and ca. 46 mol% of alendronate (ca.1 AL per BDC). The relative molar loading values are compared to those from the dual drug loaded samples in Table S4.

Table S4. Comparison of relative molecular drug loadings for the dual- and triple-drug modulated MOF samples. Drug loading values by mass for the samples used in drug delivery experiments are given in Table 1 of the main manuscript.

**MOF**	**Drug Loading / mol % compared to BDC (^{1}H NMR)**			
	**DCA**	**AL**	**α-CHC**	**IBU**
**AL/DCA@UiO-66**	18.8%	38.1%	–	–
**α-CHC/DCA@UiO-66**	35.0%	–	6.6%	–
**IBU/DCA@UiO-66**	37.1%	–	–	2.9%
**α-CHC/AL/DCA@UiO-66**	30.0%	46%	7.5%	–

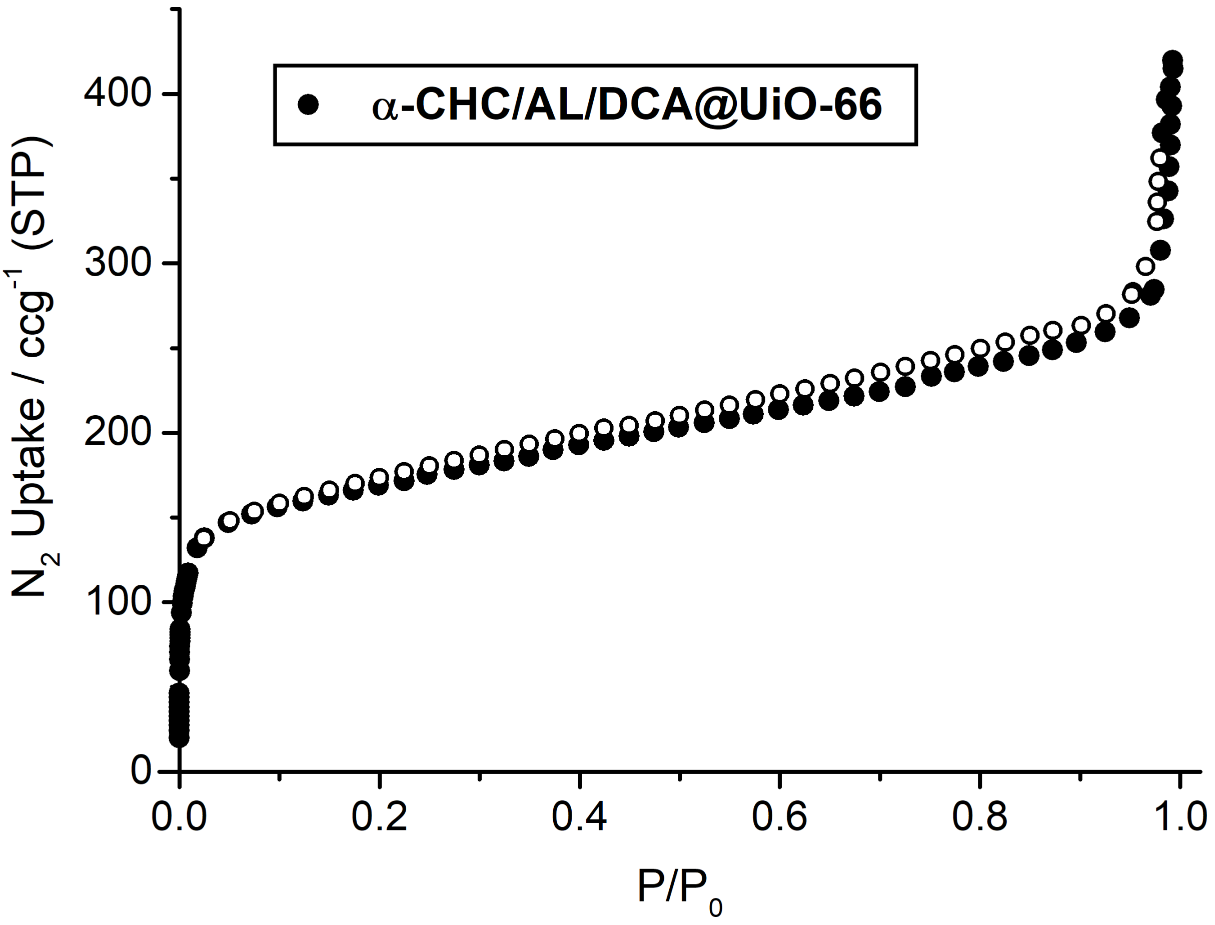

Figure S40. N_2 adsorption (filled symbols) and desorption (empty symbols) measurements at 77 K of **α-CHC/AL/DCA@UiO-66**, showing a reasonable porosity despite the incorporation of significant amounts of drugs as modulators and possible poor crystallinity induced by AL incorporation:

α-CHC/Al/DCA@UiO-66: S_{BET} = 634 m^2g^{-1}; pore volume = 0.42 ccg^{-1}.

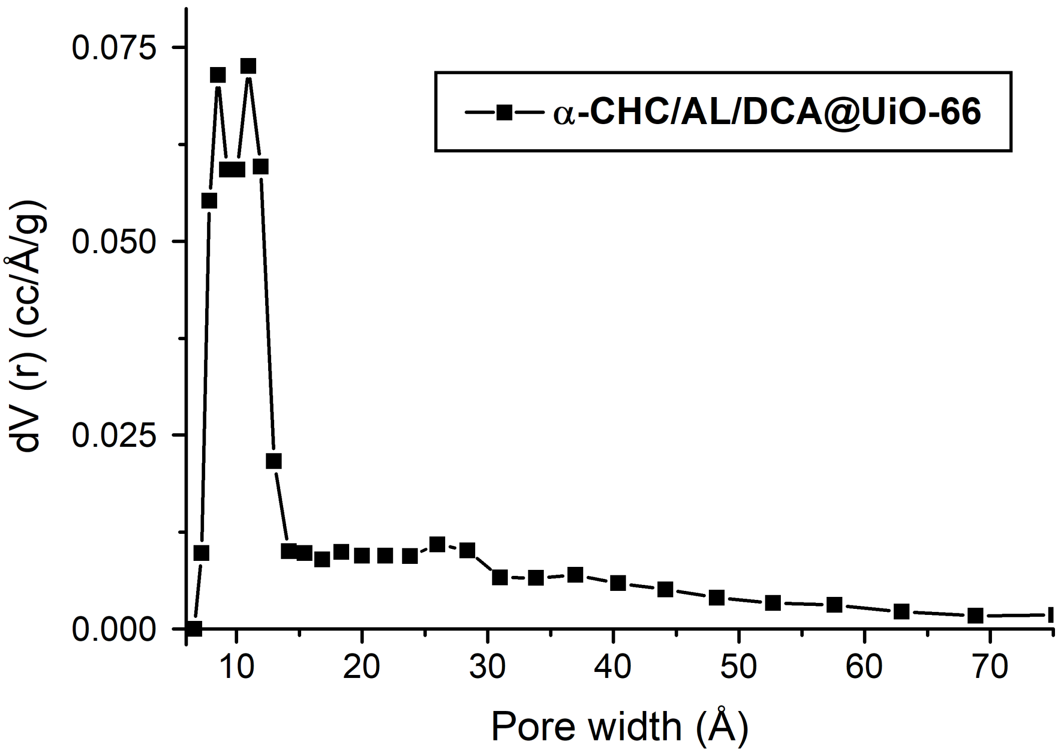

Figure S41. Pore size distribution of **α-CHC/AL/DCA@UiO-66**, showing attenuated pore volume but maintaining two pores of approximate diameter of 8 Å and 11 Å, characteristic of UiO-66.[S2]

Table S5. Comparison of relative drug loadings (molar % compared to BDC) with BET surface area and pore volume calculated from N_2 adsorption isotherms (77 K). Generally, increased **DCA** contents improve porosity through defectivity, but significant **AL** loading lower porosity, likely due to causing structural change evident in PXRD. Typical values for pristine UiO-66 are S_{BET} = 1200 m^2g^{-1} and pore volume = 0.42 cc/g^{-1}.[S2]

**MOF**	**Drug Loading / mol % compared to BDC (^{1}H NMR)**					**S_{BET} / m^2g^{-1}**	**Pore Vol / ccg^{-1}**
	**DCA**	**5-FU**	**AL**	**α-CHC**	**IBU**		
**DCA@UiO-66**	36.0	–	–	–	–	1488[a]	0.68
**α-CHC@UiO-66**	24.0	–	–	–	–	989	0.46
**IBU@UiO-66**	–	–	–	–	3.6	1111	0.60
**AL@UiO-66**	–	–	9.9	–	–	1245	0.56
**AL@DCA@UiO-66**	18.8	–	38.1	–	–	389	0.35
**α-CHC@DCA@UiO-66**	35.0	–	–	6.6	–	1485	0.77
**IBU@DCA@UiO-66**	37.1	–	–	–	2.9	1440	0.78
**α-CHC/AL/DCA@UiO-66**	30.0	–	46.0	7.5	–	634	0.42

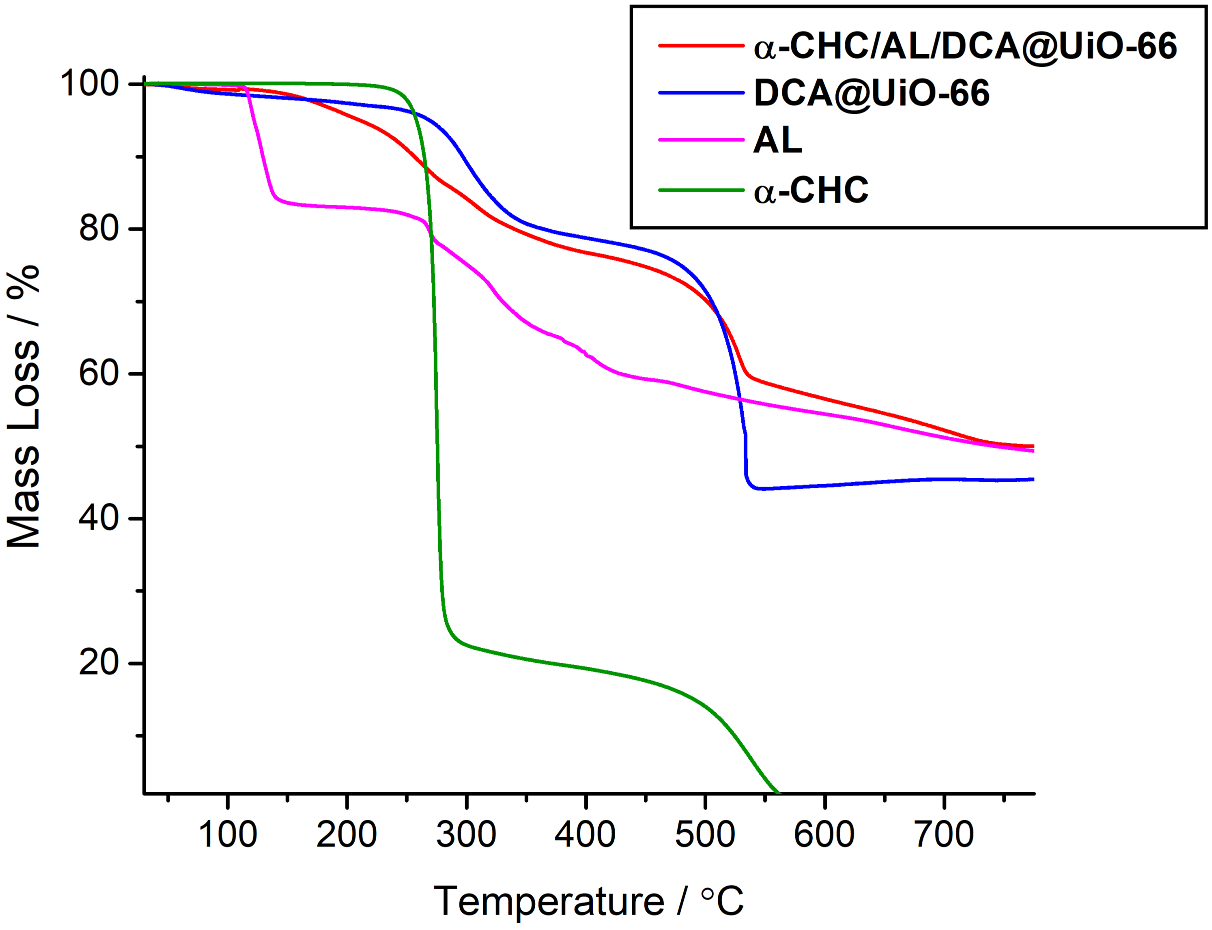

Figure S42. TGA profile of **α-CHC/AL/DCA@UiO-66** compared to **DCA@UiO-66**, AL, and α-CHC showing a broad gradual decomposition starting at lower temperatures than pristine UiO-66, indicating drug-loaded defective samples. Quantification of individual drug loading is not possible using this technique, but the mass loss associated with the decomposition of the bdc ligand around 500 °C is significantly attenuated, indicating high loading of drug modulators.

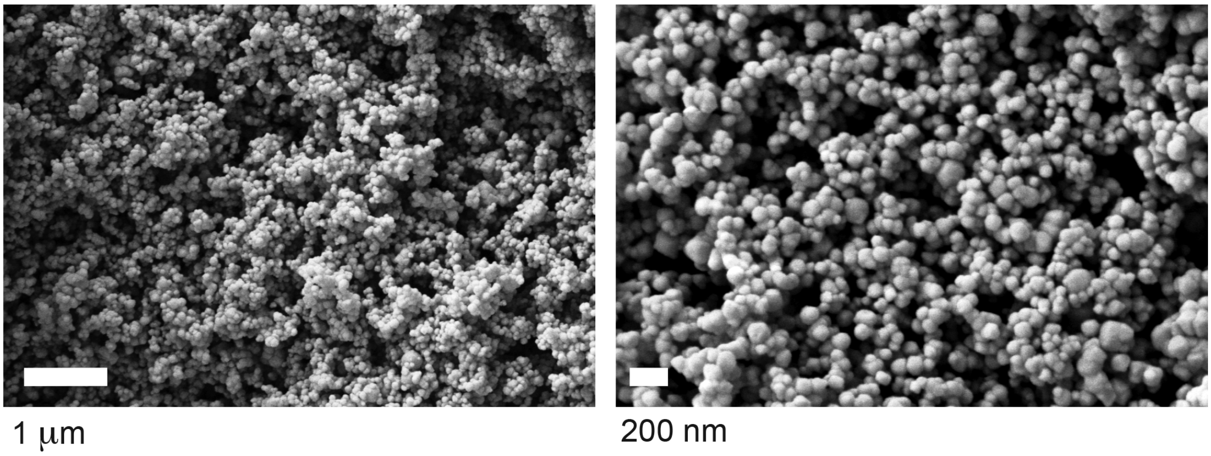

Figure S43. SEM images of **α-CHC/AL/DCA@UiO-66**, showing monodisperse nanoparticles of ca. 100 nm.

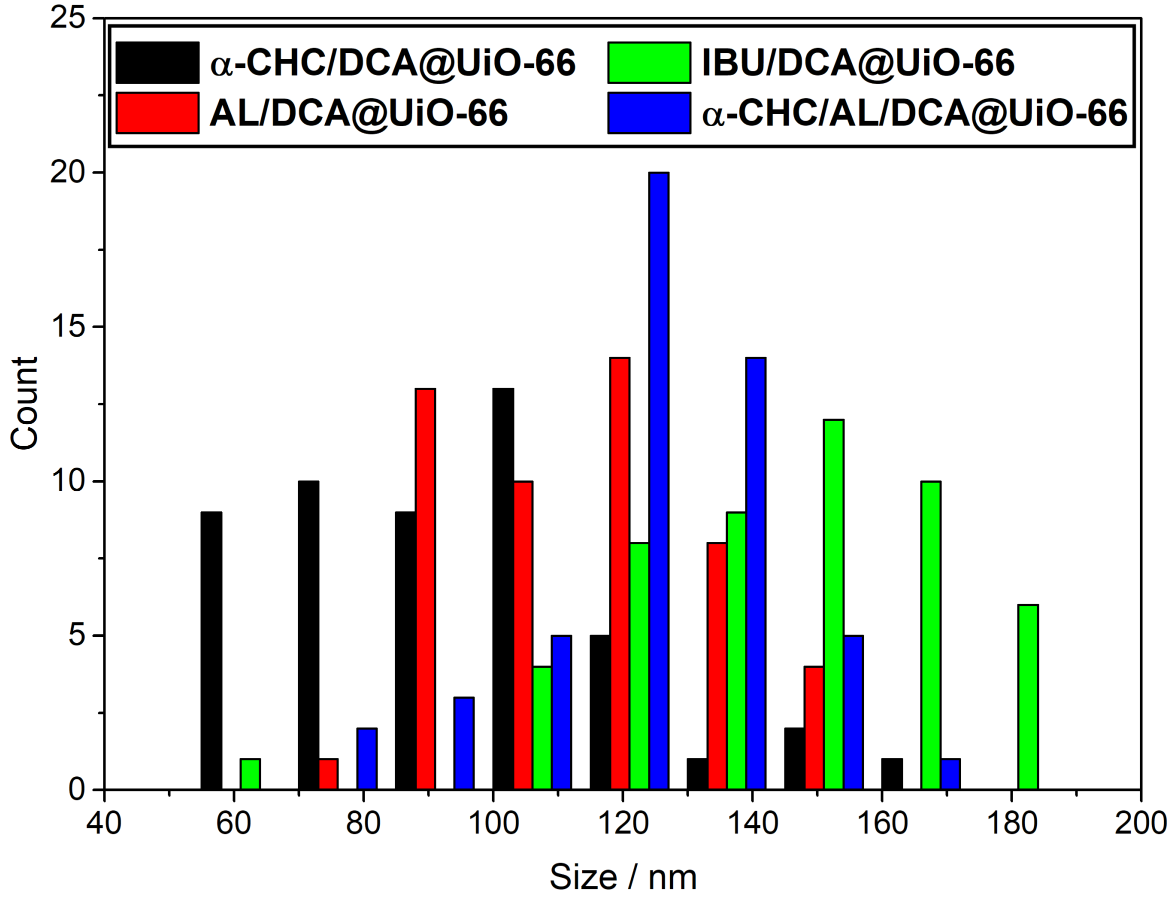

Figure S44. Particle size histogram of **α-CHC/AL/DCA@UiO-66** compared to the dual drug loaded MOFs, calculated from a minimum of 50 particles per MOF, showing that it has average particle size within the middle of the range displayed by the dual drug loaded MOFs. Barring minor differences, size control is retained in all cases by using DCA as a modulator.

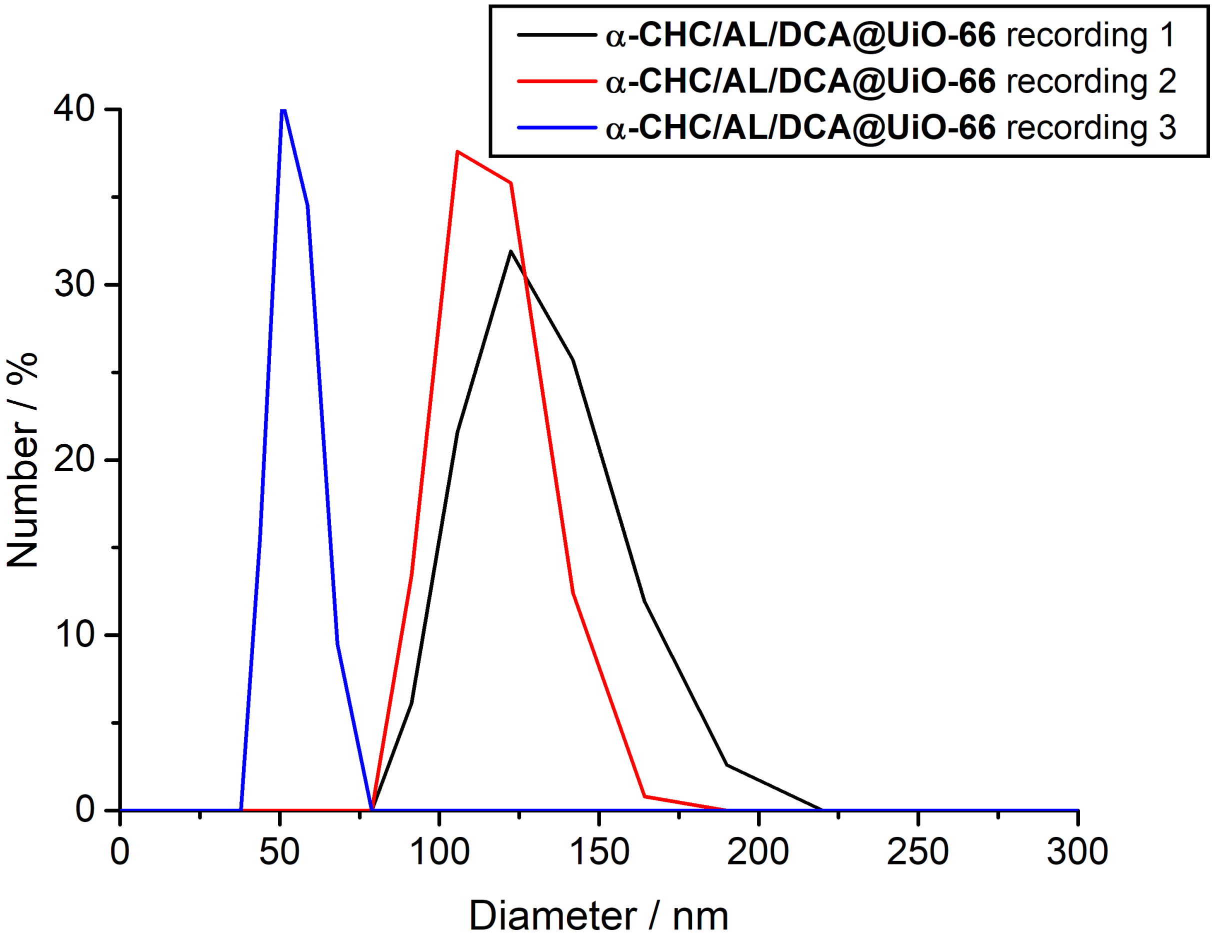

Figure S45. DLS measurements (0.1 $mgmL^{-1}$ in PBS 10X) of **α-CHC/AL/DCA@UiO-66** showing hydrodynamic diameter (ca. 125 nm) close to the one determined by SEM (ca. 125 nm).

S4.Cytotoxicity Studies

S4.1 *In Vitro* Cytotoxicity of Free Drugs

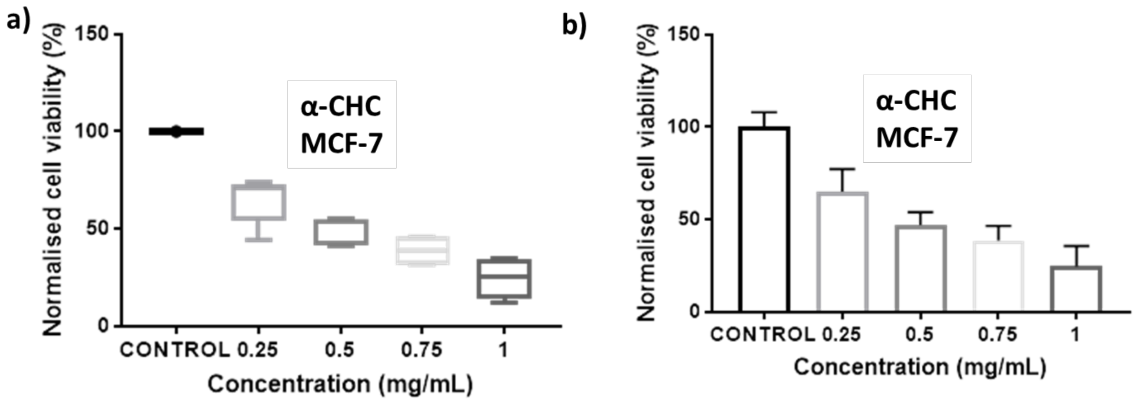

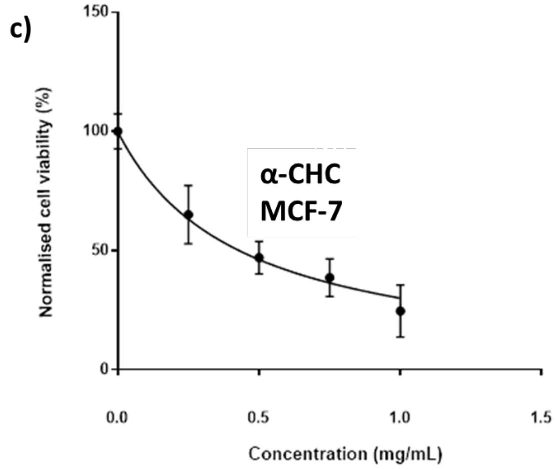

d)

Concentration α-CHC (mg/mL)	Cell viability (%) MCF-7	SD %
0	100	7.40
0.25	65.07	12.18
0.5	47.06	6.82
0.75	38.65	7.90
1	24.68	10.97

Figure S46. MTS cell viability measurements of MCF-7 cells after 72 hours of incubation with α-CHC. a) Representation of the statistical analysis of five independent experiments, each of them with n = 5. b) Representation of the average mean ± standard deviation. c) Inhibitor (drug) concentration versus normalised response (mean ± error) curve used to calculate IC_{50} values given in Table S6. d) Tabulated mean cell proliferation and standard deviations of the three independent experiments.

a)

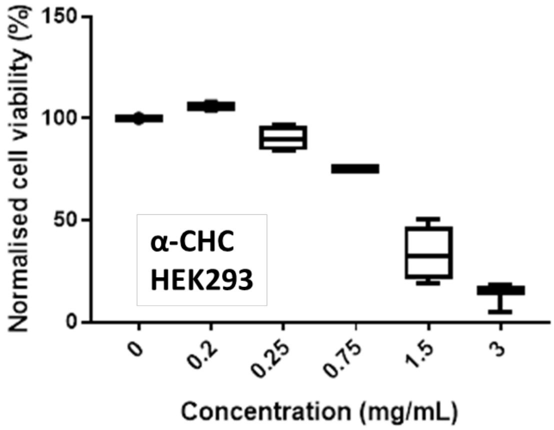

b)

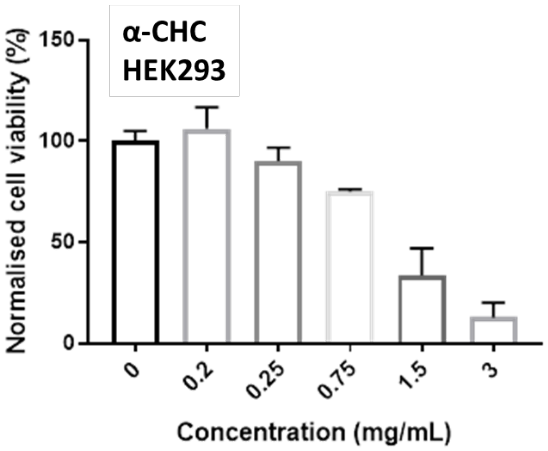

c)

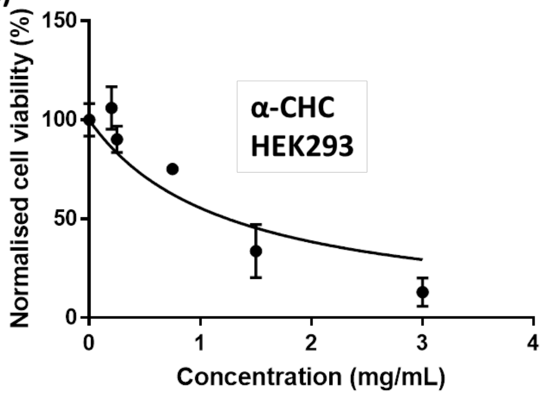

d)

Concentration α-CHC (mg/mL)	Cell viability (%) HEK293	SD %
0	100	8.328117
0.2	106.1	10.73896
0.25	90.2	6.642
0.75	75.2	0.9835
1.5	33.7	13.37
3	12.93	7.153

Figure S47. MTS cell viability measurements of HEK293 cells after 72 hours of incubation with α-CHC. a) Representation of the statistical analysis of four independent experiments, each of them with n = 5. b) Representation of the average mean ± standard deviation. c) Inhibitor (drug) concentration versus normalised response (mean ± error) curve used to calculate IC_{50} values given in Table S6. d) Tabulated mean cell proliferation and standard deviations of the three independent experiments.

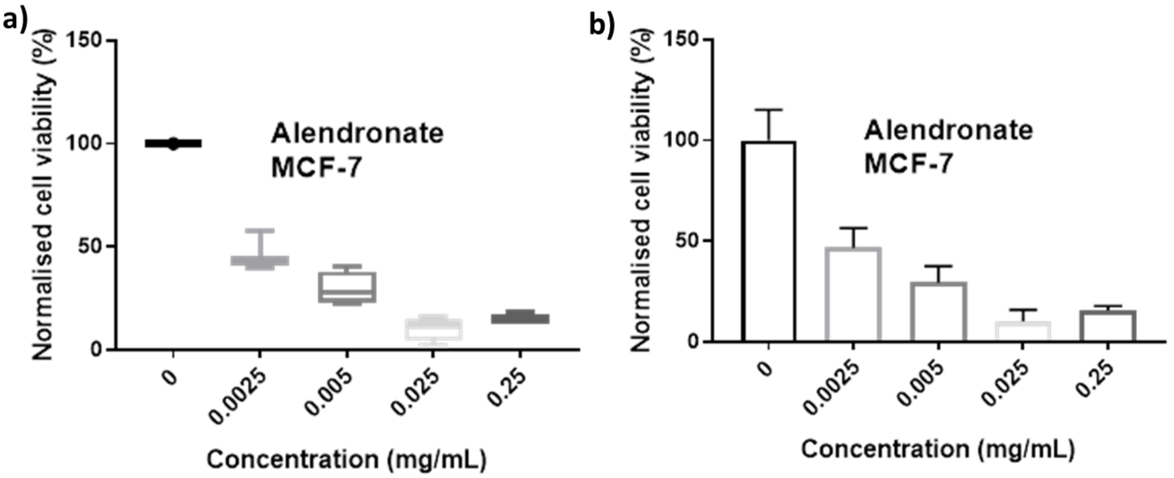

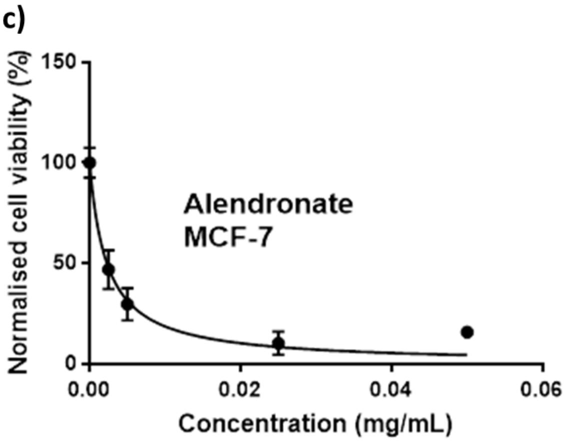

d)

Concentration Alendronate (mg/mL)	Cell viability (%) MCF-7	SD %
0	100	7.49
0.0025	46.92	9.66
0.005	29.7	7.99
0.025	10.29	5.75
0.05	15.81	2.11

Figure S48. MTS cell viability measurements of MCF-7 cells after 72 hours of incubation with AL. a) Representation of the statistical analysis of four independent experiments, each of them with n = 5. b) Representation of the average mean ± standard deviation. c) Inhibitor (drug) concentration versus normalised response (mean ± error) curve used to calculate IC_{50} values given in Table S6. d) Tabulated mean cell proliferation and standard deviations of the four independent experiments.

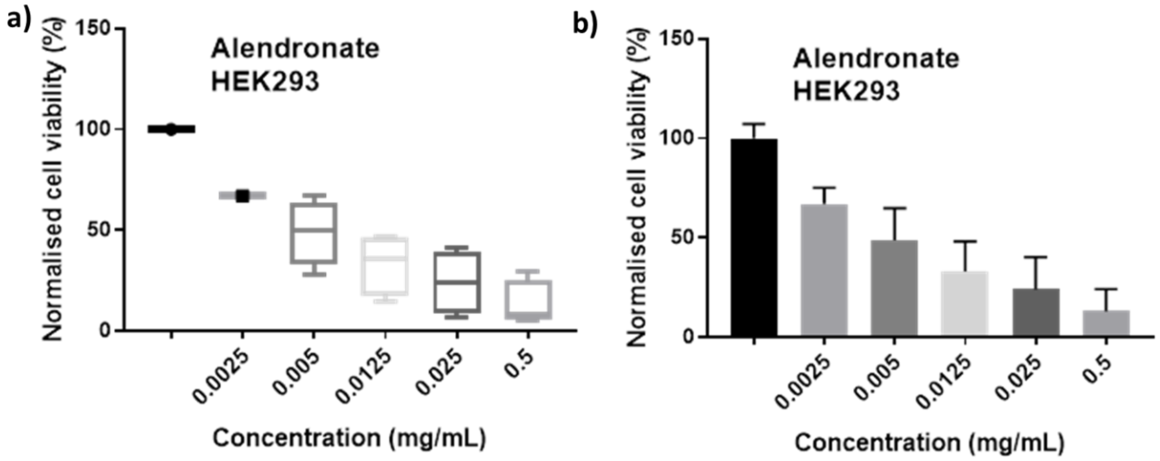

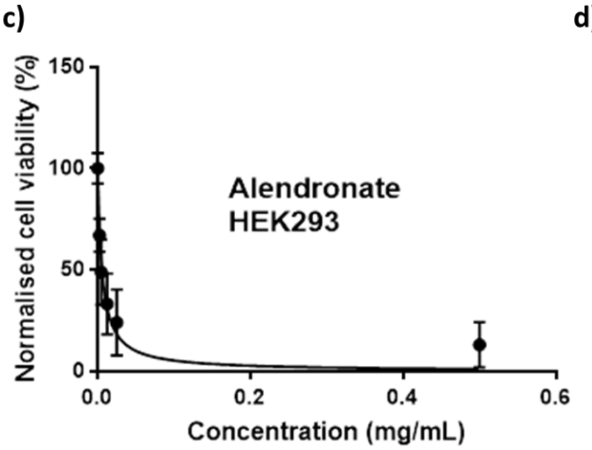

d)

Concentration Alendronate (mg/mL)	Cell viability (%) HEK-293	SD %
0	100	7.62
0.0025	67.08	8.1
0.005	48.75	16.15
0.0125	33.14	15.1
0.025	24.12	16.25
0.5	13.02	11.19

Figure S49. MTS cell viability measurements of HEK293 cells after 72 hours of incubation with AL. a) Representation of the statistical analysis of four independent experiments, each of them with n = 5. b) Representation of the average mean ± standard deviation. c) Inhibitor (drug) concentration versus normalised response (mean ± error) curve used to calculate IC_{50} values given in Table S6. d) Tabulated mean cell proliferation and standard deviations of the four independent experiments.

a)

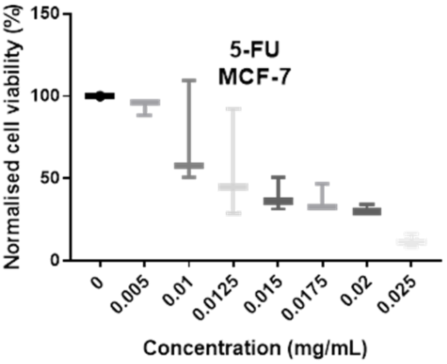

b)

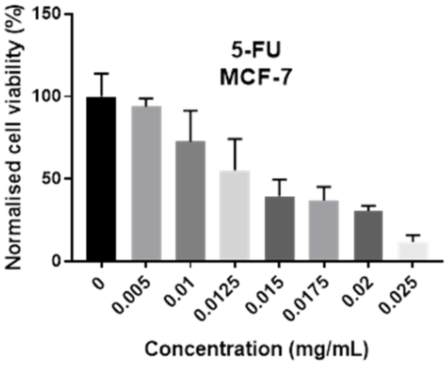

c)

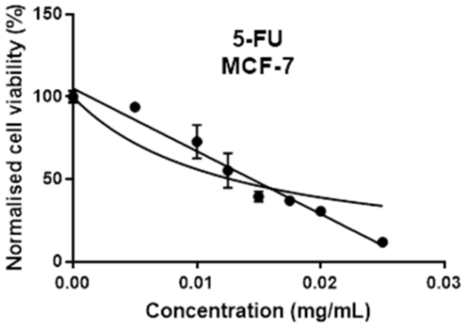

d)

Concentration 5-FU (mg/mL)	Cell viability (%) MCF-7	SD %
0	100	8
0.005	93.83	5.00
0.01	72.82	32.21
0.0125	55.3	32.94
0.015	39.53	10.16
0.0175	37.07	8.302
0.02	30.73	3.11
0.025	11.89	4.01

Figure S50. MTS cell viability measurements of MCF-7 cells after 72 hours of incubation with 5-FU. a) Representation of the statistical analysis of three independent experiments, each of them with n = 5. b) Representation of the average mean ± standard deviation. c) Inhibitor (drug) concentration versus normalised response (mean ± error) curve used to calculate IC_{50} values given in Table S6. d) Tabulated mean cell proliferation and standard deviations of the three independent experiments.

a) b)

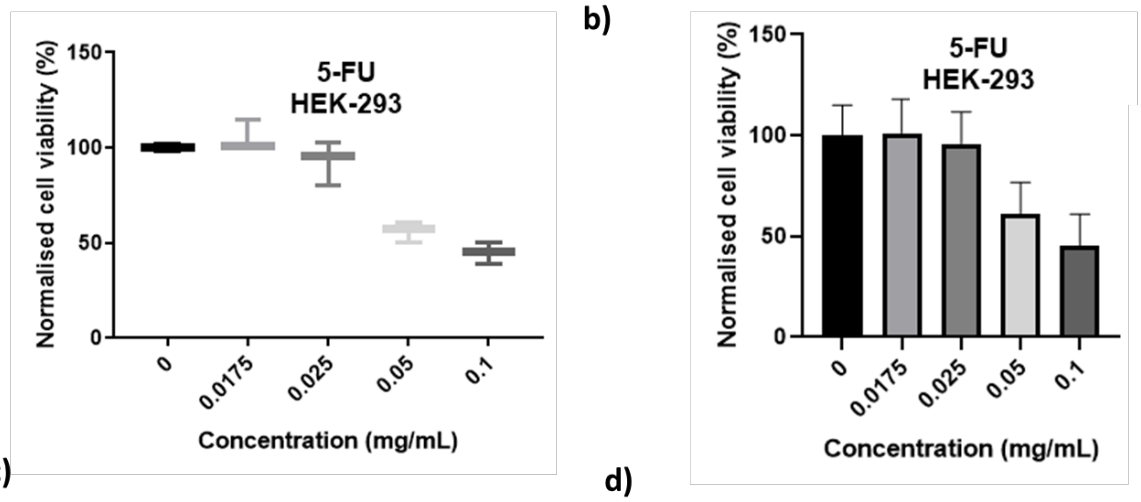

c)

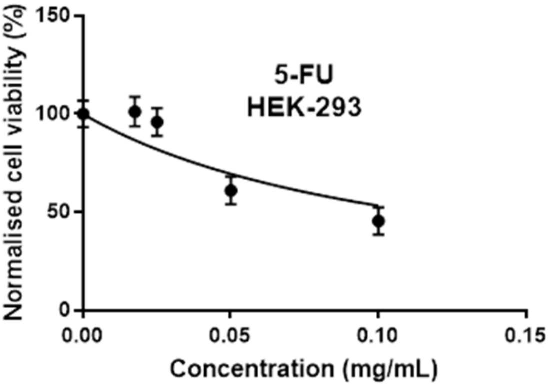

d)

Concentration 5-FU (mg/mL)	HEK293 Cell normalised viability	SD %
0	100	15.10
0.0175	101.19	16.89
0.025	95.89	15.80
0.05	69.06	15.78
0.1	45.54	15.51

Figure S51. MTS cell viability measurements of HEK293 cells after 72 hours of incubation with 5-FU. a) Representation of the statistical analysis of three independent experiments, each of them with n = 5. b) Representation of the average mean ± standard deviation. c) Inhibitor (drug) concentration versus normalised response (mean ± error) curve used to calculate IC_{50} values given in Table S6. d) Tabulated mean cell proliferation and standard deviations of the three independent experiments.

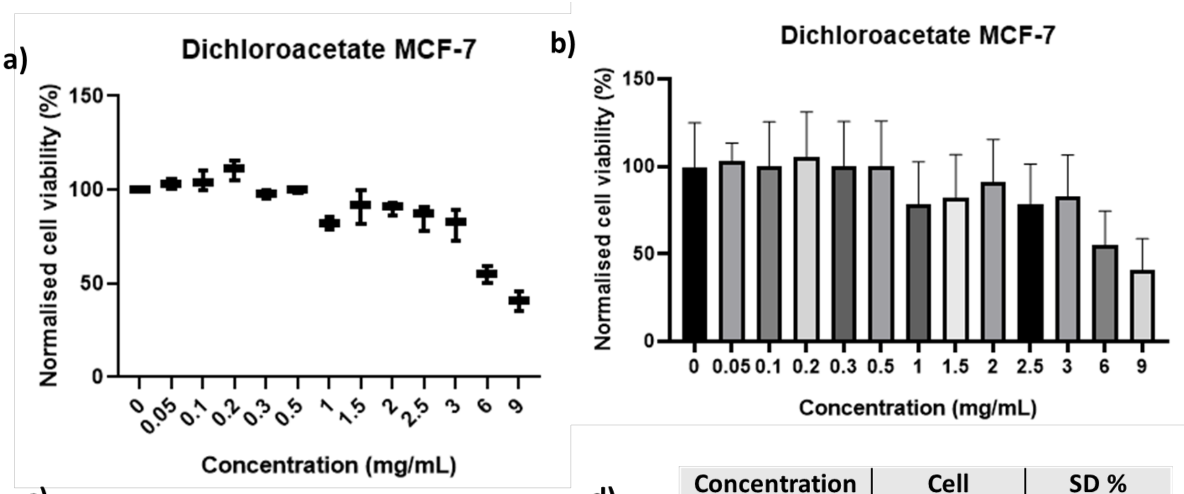

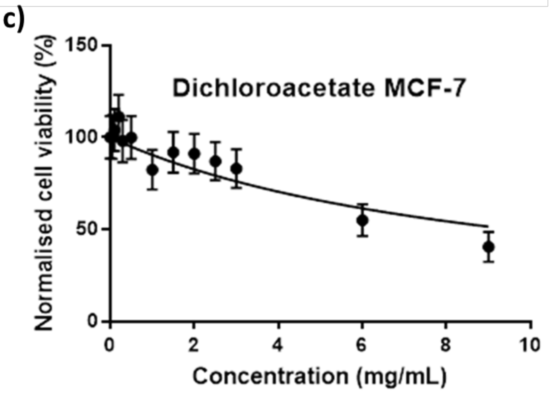

d)

Concentration DCA (mg/mL)	Cell viability (%) MCF-7	SD %
0	100	11.76
0.05	100,35	26,57
0.1	104,06	25,32
0.2	111,37	26,71
0.3	98,00	25,71
0.5	99,88	26,02
1	82,44	24,06
1.5	91,94	24,75
2	91,16	24,22
2.5	87,088	23,27
3	83,06	23,54
6	55,026	19,39
9	40,56	18,165

Figure S52. MTS cell viability measurements of MCF-7 cells after 72 hours of incubation with DCA. a) Representation of the statistical analysis of two independent experiments, each of them with n = 5. b) Representation of the average mean ± standard deviation. c) Inhibitor (drug) concentration versus normalised response (mean ± error) curve used to calculate IC_{50} values given in Table S6. d) Tabulated mean cell proliferation and standard deviations of the two independent experiments.

Table S6. Calculated IC_{50} values for free drugs against both cell lines.

Drug	IC_{50} Value / $mgmL^{-1}$	
	MCF-7	HEK293
AL	0.002297 ± 0.000252	0.00561 ± 0.000786
5-FU	0.01278 ± 0.001175	0.144 ± 0.024
α-CHC	0.4302 ± 0.03721	1.248 ± 0.1959
DCA	9.577 ± 2.807	–

S4.2. *In Vitro* Cytotoxicity of Drug-Loaded MOFs

a) b)

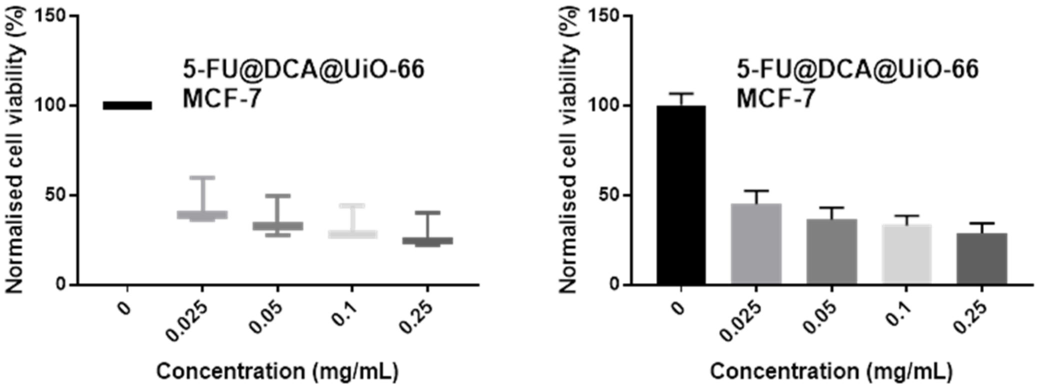

c)

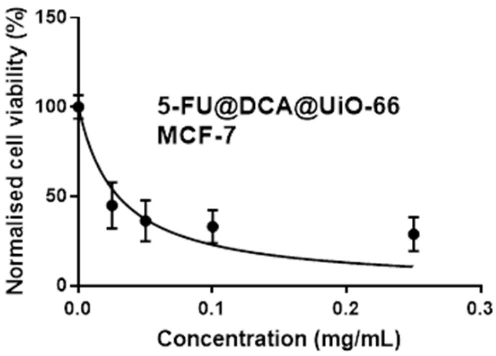

d)

MOF Concentration (mg/mL)	Cell viability (%) MCF-7	SD %
0	100	6.60
0.025	44.93	12.87
0.05	36.27	11.58
0.1	33	9.365
0.25	28.77	9.492

Figure S53. MTS cell viability measurements of MCF-7 cells after 72 hours of incubation with **5-FU@DCA@UiO-66**. a) Representation of the statistical analysis of three independent experiments, each of them with n = 5. b) Representation of the average mean ± standard deviation. c) Inhibitor (MOF) concentration versus normalised response (mean ± error) curve used to calculate IC_{50} values given in Table S7. d) Tabulated mean cell proliferation and standard deviations of the three independent experiments.

a) b)

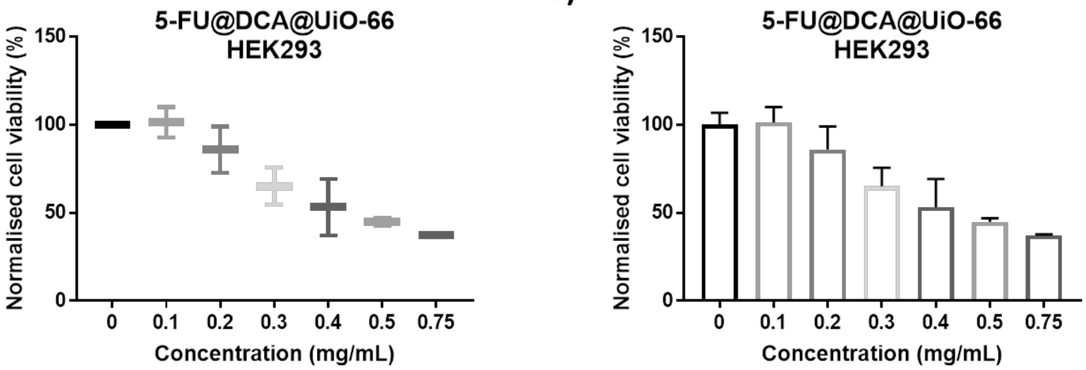

c)

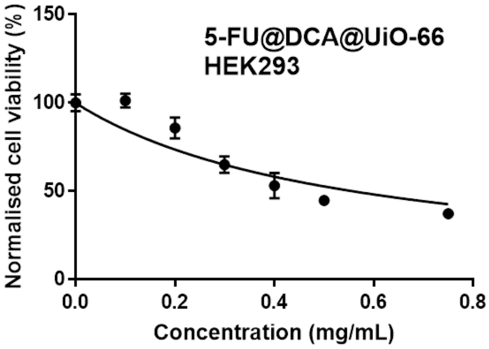

d)

MOF Concentration (mg/mL)	Cell viability (%) HEK-293	SD
0	100	15.10
0.1	101.3	12.42
0.2	85.83	18.68
0.3	65.09	14.73
0.4	53.19	22.56
0.5	44.83	2.992
0.75	37.33	0.6187

Figure S54. MTS cell viability measurements of HEK293 cells after 72 hours of incubation with **5-FU@DCA@UiO-66**. a) Representation of the statistical analysis of three independent experiments, each of them with n = 5. b) Representation of the average mean ± standard deviation. c) Inhibitor (MOF) concentration versus normalised response (mean ± error) curve used to calculate an IC_{50} values of 558.1 ± 54.0 µgmL^{-1}. d) Tabulated mean cell proliferation and standard deviations of the three independent experiments.

a) b)

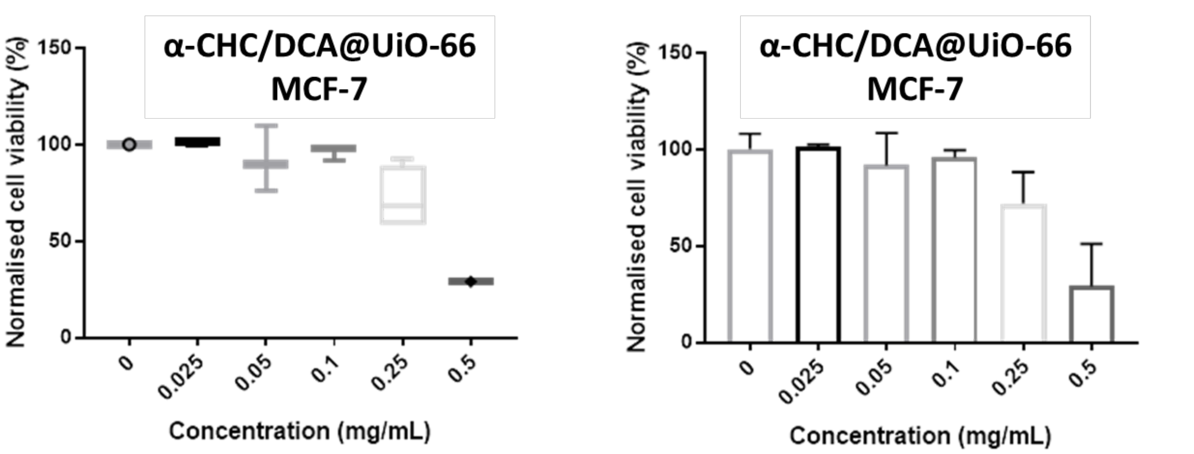

c)

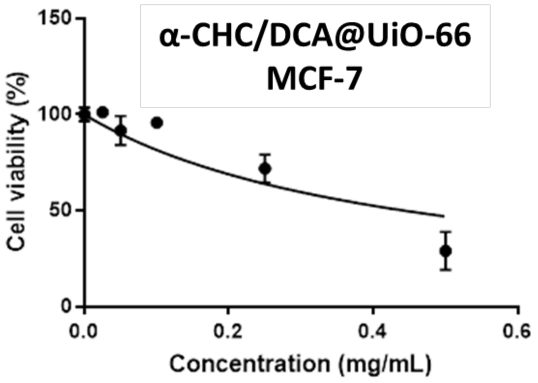

d)

MOF Concentration (mg/mL)	Cell viability (%) MCF-7	SD %
0	100	8
0.025	101.2	1.32
0.05	91.64	16.77
0.1	95.82	3.79
0.25	71.92	16.30
0.5	29.05	22.01

Figure S55. MTS cell viability measurements of MCF-7 cells after 72 hours of incubation with **α-CHC/DCA@UiO-66**. a) Representation of the statistical analysis of three independent experiments, each of them with n = 5. b) Representation of the average mean ± standard deviation. c) Inhibitor (MOF) concentration versus normalised response (mean ± error) curve used to calculate IC_{50} values given in Table S7. d) Tabulated mean cell proliferation and standard deviations of the three independent experiments.

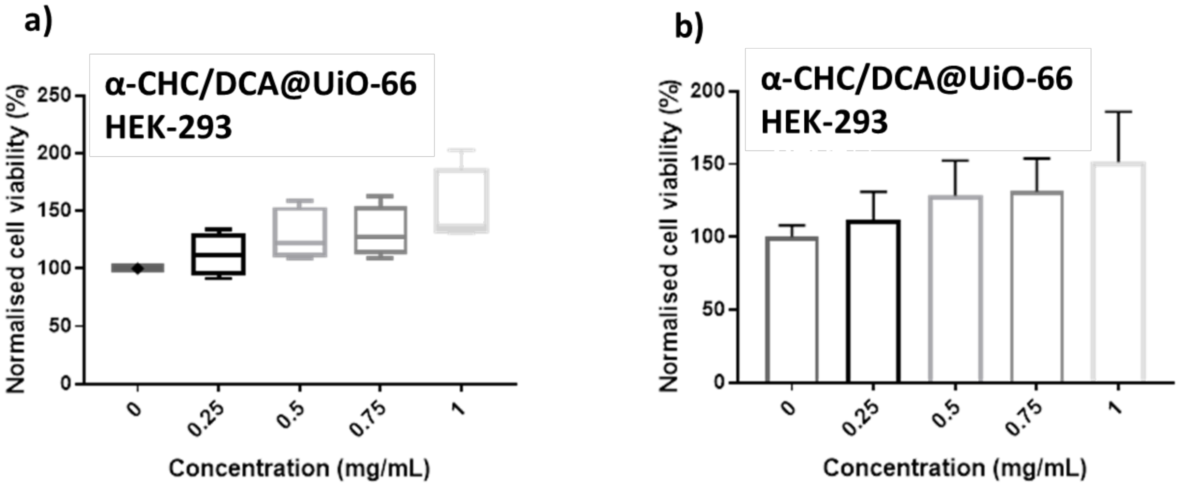

c)

MOF Concentration (mg/mL)	Cell viability (%) HEK-293	SD
0	100	10.48
0.25	111.83	11.18
0.5	128.41	10.25
0.75	131.40	15.62
1	151.436	17.55

Figure S56. MTS cell viability measurements of HEK293 cells after 72 hours of incubation with **α-CHC/DCA@UiO-66**. a) Representation of the statistical analysis of four independent experiments, each of them with n = 5. b) Representation of the average mean ± standard deviation. c) Tabulated mean cell proliferation and standard deviations of the three independent experiments. An IC_{50} value could not be calculated as proliferation did not drop at the concentrations measured.

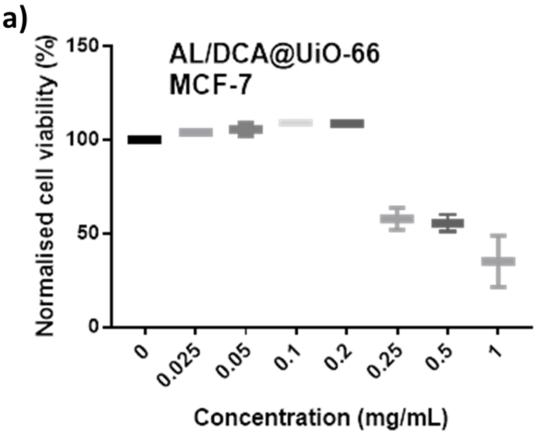

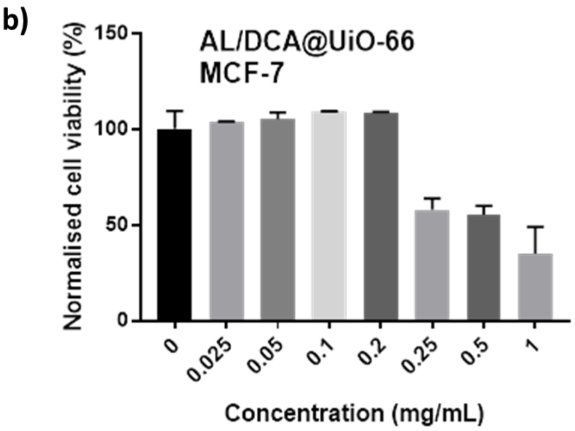

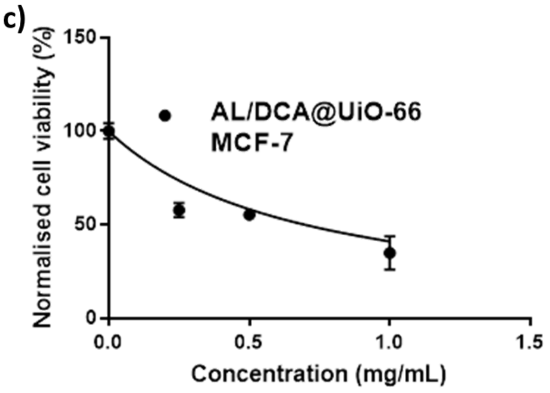

d)

MOF Concentration (mg/mL)	Cell viability (%) MCF-7	SD %
0	100	9.41
0.2	108.3	1.046
0.25	57.79	8.425
0.5	55.31	6.54
1	34.97	19.79

Figure S57. MTS cell viability measurements of MCF-7 cells after 72 hours of incubation with **AL/DCA@UiO-66**. a) Representation of the statistical analysis of two independent experiments, each of them with n = 5. b) Representation of the average mean ± standard deviation. c) Inhibitor (MOF) concentration versus normalised response (mean ± error) curve used to calculate IC_{50} values given in Table S7. d) Tabulated mean cell proliferation and standard deviations of the two independent experiments.

a) b)

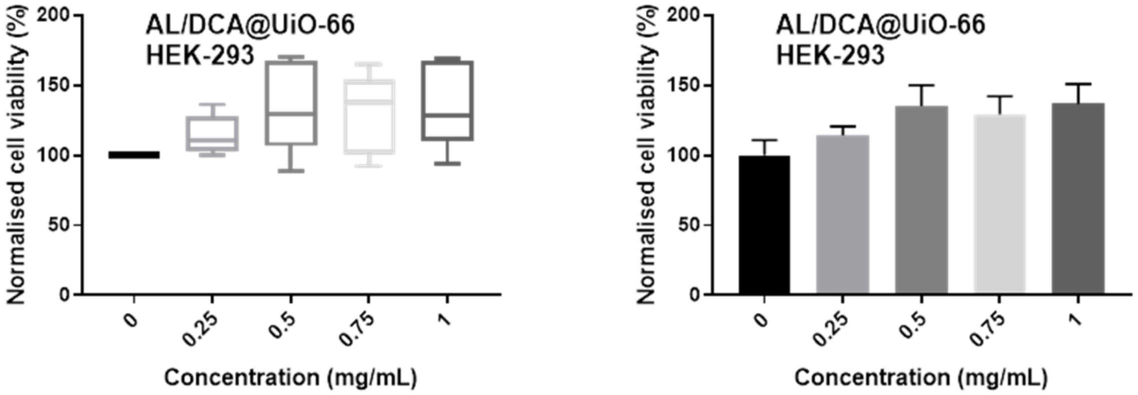

c)

MOF Concentration (mg/mL)	Cell viability (%) HEK293	SD
0	100	8
0.25	114.5	14.18
0.5	135.3	33.03
0.75	129.5	28.71
1	137	31.3

Figure S58. MTS cell viability measurements of HEK293 cells after 72 hours of incubation with **AL/DCA@UiO-66**. a) Representation of the statistical analysis of five independent experiments, each of them with n = 5. b) Representation of the average mean ± standard deviation. c) Tabulated mean cell proliferation and standard deviations of the three independent experiments. An IC_{50} value could not be calculated as proliferation did not drop at the concentrations measured.

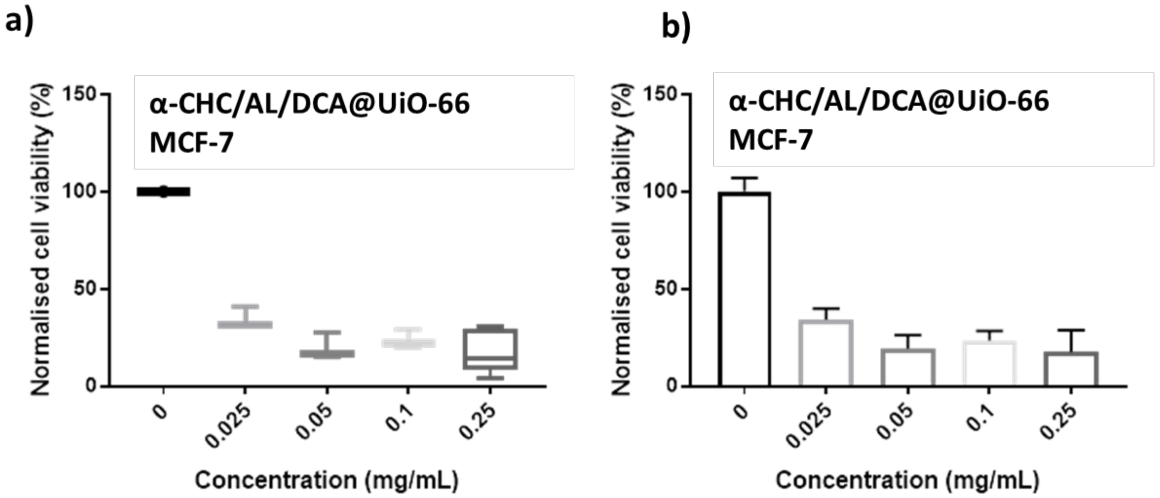

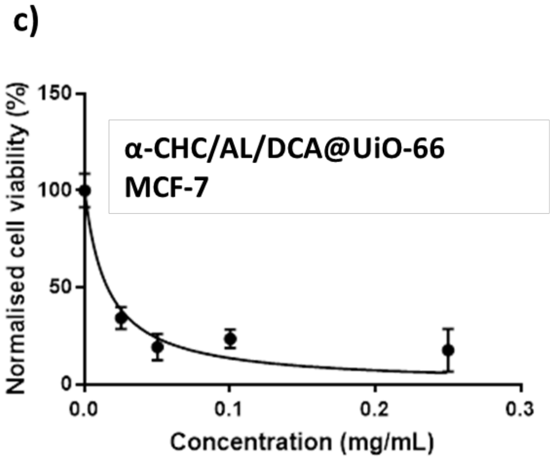

d)

MOF Concentration (mg/mL)	Cell viability (%) MCF-7	SD %
0	100	8.66
0.025	34.37	5.63
0.05	19.35	6.81
0.1	23.57	4.71
0.25	17.72	11.03

Figure S59. MTS cell viability measurements of MCF-7 cells after 72 hours of incubation with **α-CHC/AL/DCA@UiO-66**. a) Representation of the statistical analysis of two independent experiments, each of them with n = 5. b) Representation of the average mean ± standard deviation. c) Inhibitor (MOF) concentration versus normalised response (mean ± error) curve used to calculate IC_{50} values given in Table S7. d) Tabulated mean cell proliferation and standard deviations of the two independent experiments.

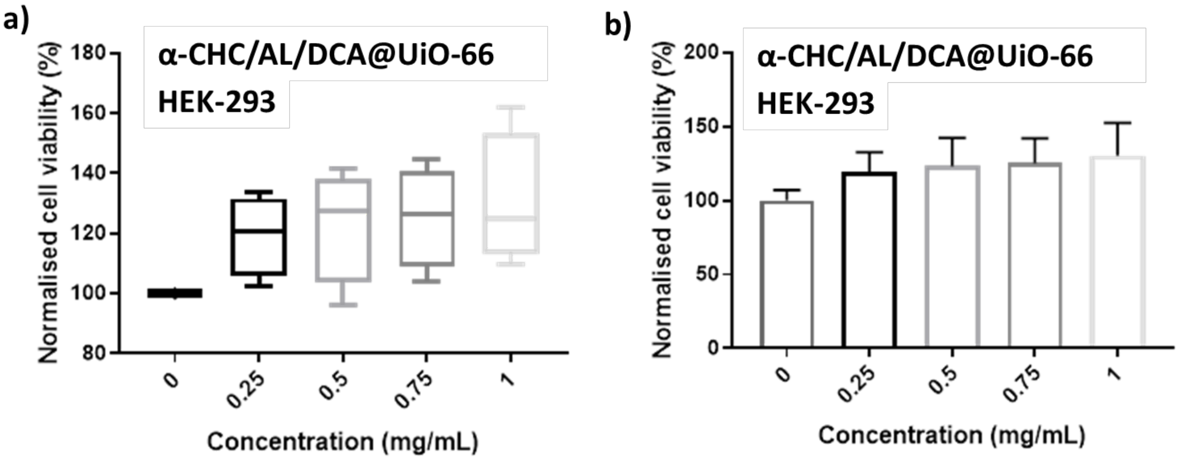

c)

MOF Concentration (mg/mL)	Cell viability (%) HEK293	SD
0	100	7.51
0.25	119.3	13.45
0.5	123.2	19.14
0.75	125.3	16.71
1	130.3	22.22

Figure S60. MTS cell viability measurements of HEK293 cells after 72 hours of incubation with **α-CHC/AL/DCA@UiO-66**. a) Representation of the statistical analysis of four independent experiments, each of them with n = 5. b) Representation of the average mean ± standard deviation. c) Tabulated mean cell proliferation and standard deviations of the five independent experiments. An IC_{50} value could not be calculated as proliferation did not drop at the concentrations measured.

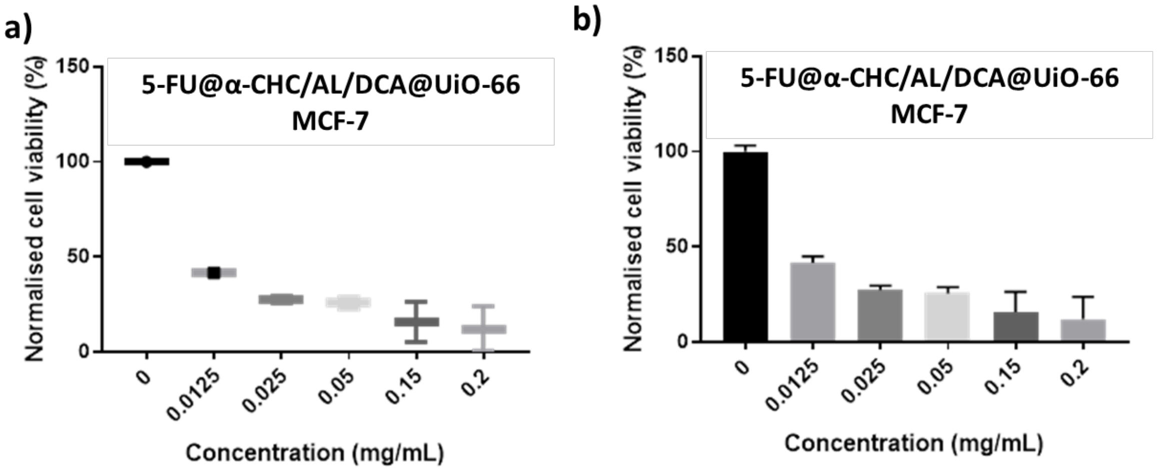

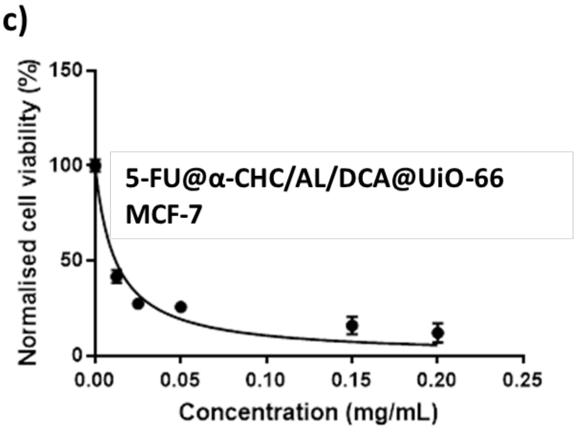

d)

MOF Concentration (mg/mL)	Cell viability (%) MCF-7	SD %
0	100	7
0.0125	41.73	7.56
0.025	27.49	3.15
0.05	25.63	4.67
0.15	15.88	14.84
0.2	12.1	16.56

Figure S61. MTS cell viability measurements of MCF-7 cells after 72 hours of incubation with **5-FU@α-CHC/AL/DCA@UiO-66**. a) Representation of the statistical analysis of three independent experiments, each of them with n = 5. b) Representation of the average mean ± standard deviation. c) Inhibitor (MOF) concentration versus normalised response (mean ± error) curve used to calculate IC_{50} values given in Table S7. d) Tabulated mean cell proliferation and standard deviations of the two independent experiments.

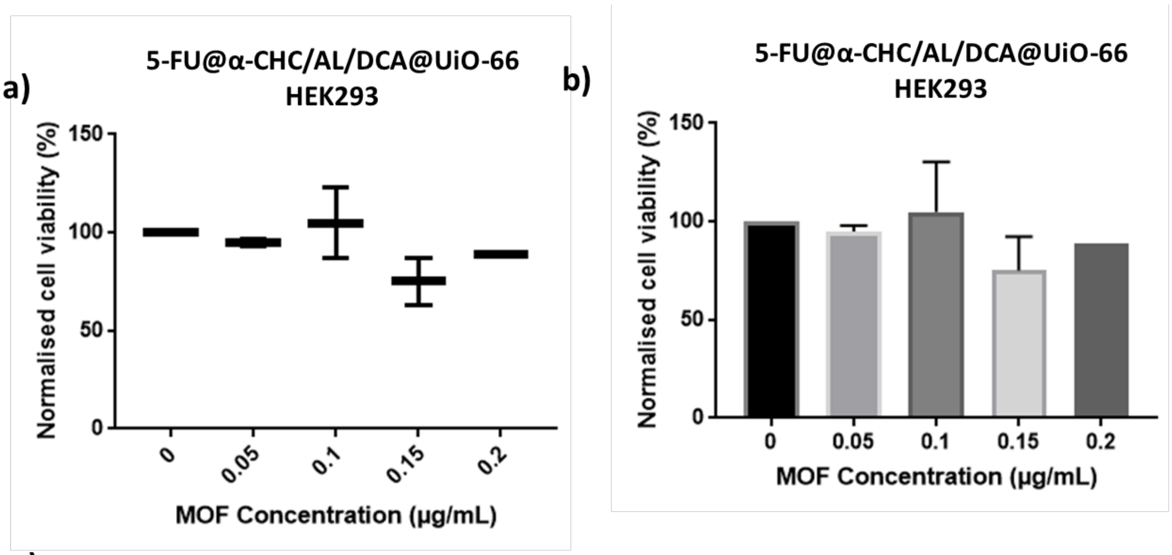

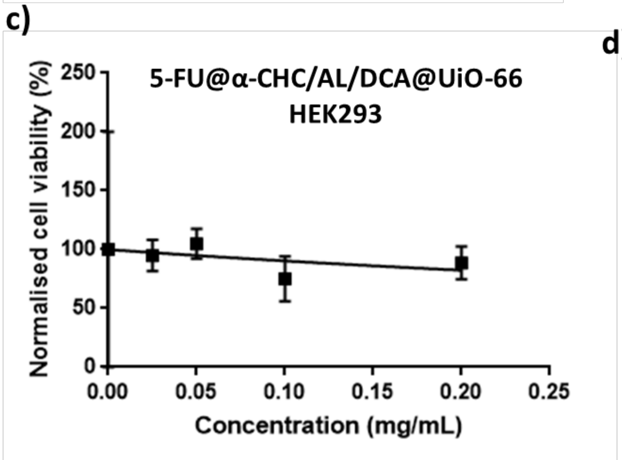

d)

MOF Concentration (mg/mL)	Cell viability (%) HEK293	SD error
0	100	5.89
0.05	94.96	3.018
0.1	104.9	25.44
0.15	75.04	17.24
0.2	88.68	0.0583

Figure S62. MTS cell viability measurements of HEK293 cells after 72 hours of incubation with **5-FU@α-CHC/AL/DCA@UiO-66**. a) Representation of the statistical analysis of two independent experiments, each of them with n = 5. b) Representation of the average mean ± standard deviation. c) Tabulated mean cell proliferation and standard deviations of the five independent experiments. An IC_{50} value could not be calculated as proliferation did not drop at the concentrations measured.

Table S7. IC_{50} values towards MCF-7 cells for the different MTVM drug-loaded MOF formulations towards MCF-7 cells, expressed first by concentration of MOF, and also by the maximum concentrations of each drug that could be delivered by that DDS.

MOF	IC_{50} / $ugmL^{-1}$ MOF	Maximum Delivered Drug Concentration / $ugmL^{-1}$			
		α-CHC	5-FU	AL	DCA
**α-CHC/DCA@UiO-66**	788.3 ± 177.9	15.67 ± 3.558			86.16 ± 19.57
**AL/DCA@UiO-66**	694.8 ± 163.9			192.8 ± 37.78	25.84 ± 5.065
**5-FU@DCA@UiO-66**	29.65 ± 4.252		0.356 ± 0.051		2.372 ± 0.34
**α-CHC/AL/DCA@UiO-66**	15.71 ± 2.235	0.314 ± 0.0447		3.393 ± 0.483	0.503 ± 0.0715
**5-FU@α-CHC/AL/DCA@UiO-66**	11.94 ± 1.389	0.191 ± 0.022	0.191 ± 0.022	2.543 ± 0.296	0.370 ± 0.0431

The fact that multiple drugs of varying cytotoxicity are present in one MOF makes it difficult to assess the individual factors that govern overall cytotoxicity. To attempt to delineate the effect of each drug, the maximum delivered concentration of drug at each IC_{50} value for MCF-7 cells is expressed as a percentage of the IC_{50} value measured for the free drug in Table S8.

Table S8. The maximum delivered dose of each drug from the MTVM formulations, expressed as a percentage of the IC_{50} of the free drug (MCF-7 cells).

MOF	Maximum Delivered Drug Concentration as Percentage of IC_{50} of Corresponding Free Drug			
	α-CHC	5-FU	AL	DCA
**α-CHC/DCA@UiO-66**	3.64%			0.90%
**AL/DCA@UiO-66**			8392%	0.27%
**5-FU@DCA@UiO-66**		2.78%		0.025%
**α-CHC/AL/DCA@UiO-66**	0.073%		147.7%	0.0052%
**5-FU@α-CHC/AL/DCA@UiO-66**	0.044%	1.49%	110.7%	0.0039%

Values that are <100% mean that the treatment is more cytotoxic than administration of the free drug alone. It is clear that for all drugs other than AL, cytotoxicity has been greatly enhanced. This is expected, as AL is the most cytotoxic of the four (see Table S6). It is suggestive that all drugs have some involvement in cytotoxicity towards MCF-7 cells, and that cytotoxicity increases as the formulations become more complex, delivering two, three,

and then four drugs concurrently. The selectivity is also enhanced, as HEK293 cells were biocompatible with all formulations up to 1 $mgmL^{-1}$ incubations other than **5-FU@DCA@UiO-66**, which resulted in an IC_{50} value of 558.1 ± 54.0 μgmL^{-1} (c.f. 29.65 ± 4.252 μgmL^{-1} towards MCF-7) expressed as MOF dosage.

S5. References

[S1] I. Abánades Lázaro, S. Haddad, J. M. Rodrigo-Muñoz, C. Orellana-Tavra, V. del Pozo, D. Fairen-Jimenez, R. S. Forgan, *ACS Appl. Mater. Interfaces* **2018**, *10*, 5255-5268.

[S2] J. H. Cavka, S. Jakobsen, U. Olsbye, N. Guillou, C. Lamberti, S. Bordiga, K. P. Lillerud, *J. Am. Chem. Soc.* **2008**, *130*, 13850-13851.
